# Halogen-Bond-Mediated ^13^C Overhauser Dynamic Nuclear Polarization at 9.4 T

**DOI:** 10.1021/acs.jpclett.5c00798

**Published:** 2025-04-28

**Authors:** Luming Yang, Tomas Orlando, Marina Bennati

**Affiliations:** † Research Group ESR Spectroscopy, Max Planck Institute for Multidisciplinary Sciences, Am Fassberg 11, 37077 Göttingen, Germany; ‡ Institute of Physical Chemistry, Department of Chemistry, Georg-August-University, Tammanstr. 6, 37077 Göttingen, Germany

## Abstract

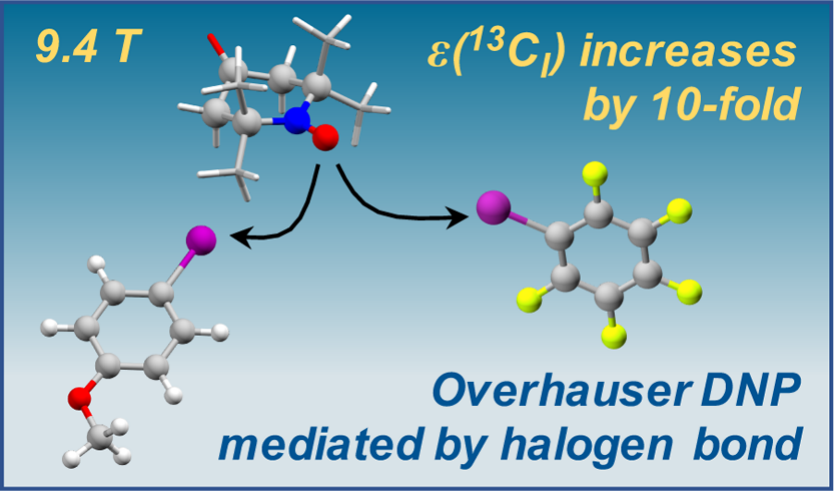

Overhauser dynamic nuclear polarization (OE-DNP) is capable of enhancing solution ^13^C NMR signals of analytes by 1–2 orders of magnitude through spin polarization transfer from paramagnetic polarizing agents, usually nitroxide radicals, at magnetic fields relevant for high-resolution NMR spectroscopy (≳9 T). While some halogen atoms have been revealed to mediate OE-DNP on adjacent ^13^C, methods of promoting OE-DNP through halogen bond (XB) design were not well-understood. Here we investigate OE-DNP of selected halogenated compounds by tuning their XB strengths to the nitroxide radicals through molecular design. Up to 10-fold boosts in OE-DNP enhancements were achieved by increasing the analyte XB donor strength in selected iodinated and brominated derivatives. Furthermore, we observed strong correlation between OE-DNP performance and XB properties for compounds sharing similar XB binding sites. Our results suggest new possibilities for designing hyperpolarized probes and labels for biosensors and the study of biomolecular processes.

High-resolution NMR spectroscopy is an indispensable analytical method for molecular structure elucidation, quantification, and studying of host–guest interactions. Particularly, ^13^C-detected NMR plays an invaluable role in probing the chemical environments of organic molecules.^1^ However, due to low gyromagnetic ratio and natural abundance of the NMR-active isotope, ^13^C NMR often encounters sensitivity issues, especially in two- and multidimensional correlation experiments, where large sample amounts, prolonged experiment times, or expensive isotopic labeling is required. One way of boosting NMR sensitivity is through hyperpolarization, whereby the NMR signal intensity is increased by shifting the nuclear polarization out of thermal equilibrium.^2−4^ Besides reduction in experiment time, hyperpolarization further enables analysis of systems that are otherwise challenging to study by conventional NMR. Methods such as dissolution dynamic nuclear polarization (dDNP) have enabled fast characterization of protein–ligand binding dynamics through the use of hyperpolarized ligands.^5,6^*In vivo* metabolic ^13^C imaging has been achieved by using hyperpolarized molecular probes.^7,8^ Other methods, including surface-enhanced NMR spectroscopy^9^ and photochemically induced dynamic nuclear polarization (photo-CIDNP),^10^ have been implemented to study nanoscopic surfaces and dilute biologically relevant systems.

Overhauser dynamic nuclear polarization (OE-DNP) has recently attracted increasing attention for hyperpolarizing ^13^C NMR at high magnetic fields (≥9.4 T) in solution.^11−15^ In this method, a steady-state enhanced nuclear polarization is generated on the analyte upon continuous microwave irradiation of its solution doped with paramagnetic molecules known as polarizing agents, the electronic spin transition of which is saturated. OE-DNP is induced by molecular motions that are ubiquitously present in solution, thus having a potentially unlimited substrate scope. It further permits continuous analyte hyperpolarization, allowing direct transfer of existing NMR methodologies.^14,15^ However, to date, the application of OE-DNP is still in its infancy, especially when related to hyperpolarized molecular probes. While recent instrument development by the authors and co-workers provides the hardware basis for OE-DNP with resolution approaching that of conventional NMR,^15^ there is still a lack of quantitative information on systems possessing strong enhancements. Instead of a screening approach, predicting OE-DNP effects *a priori*, even at a phenomenological level, would greatly expand the scope of the OE-DNP methodology.

Among the systems tested so far, halogenated small molecules, especially those with heavier halogens, have produced the largest ^13^C Overhauser DNP enhancements, ε(^13^C). At 3.4 T, up to ∼1000-fold enhancement has been achieved for carbon tetrachloride (CCl_4_) when using nitroxide radical as the polarizing agent.^16^ At 9.4 T, a magnetic field relevant for high-resolution studies, large enhancements have been reported for carbon tetrabromide (CBr_4_) (ε(^13^C) ≈ 600) and CCl_4_ (ε(^13^C) ≈ 430).^12^ Although the DNP performance of CI_4_ has not been reported, likely due to chemical instability, iodination has been shown to generate large enhancements in aromatic systems.^15^ For monohalogenated benzenes, the enhancement of iodinated ^13^C (ε ≈ 25) is significantly higher than those of ^13^C functionalized with other halogens (ε ≤ 3 for C_X_ with X = F, Cl, Br) and proton (ε ≈ 8 for benzene).^15^ For more complex drug molecules, up to 10-fold enhancements were achieved for iodinated ^13^C, which are 2 to 10 times higher than for other types of ^13^C.^15^

For these systems, a polarizing agent–analyte halogen bond (XB) has been proposed to be involved in producing NMR signal enhancements of halogenated carbon-13 atoms.^12,15^ A halogen bond is an attractive interaction between a nucleophile and the halogen σ hole, a region with positive electrostatic potential centered along the extension of the covalent bond in which the halogen participates.^17^ Previous studies have revealed XB formation between halogenated small molecules and nitroxide radicals. For instance, iodobenzene and pentafluoroiodobenzene are known to alter the spin density distribution of the nitroxide aminoxyl site through XB interaction in solution.^18^ In the solid state, XBs have been identified between tetrafluorodiiodobenzene and nitroxides by the close I···O contacts.^19^ Furthermore, the influences of XBs on physical properties of nitroxide radical adducts with fluoroiodohydrocarbons, including iodobenzene and pentafluoroiodobenzene, have been computationally studied using density functional theory (DFT) analysis.^20^ While these studies support the formation of XBs with the nitroxide polarizing agent during OE-DNP, it is not yet clear how XB properties, such as bond length, angle, and strength, influence the OE-DNP effect. Unraveling these effects could allow boosting of ^13^C enhancement by molecular design.

Attempting to shed light on this underexplored aspect of OE-DNP, we investigated the OE-DNP performance of 16 halogenated compounds using our recently developed setup,^15,21^ which permits unequivocal determination of ε(^13^C) thanks to its spectral resolution. Test subjects included 10 iodobenzene derivatives, two bromobenzene derivatives, two chlorobenzene derivatives, and two carbon tetrahalides (Scheme 1). The strength of XBs is known to be tunable by functionalization of the analyte electronic system with electron-withdrawing groups such as fluorine to influence the electrostatic potential of the halogen σ hole.^22^ For instance, crystallographic studies showed that increasing degree of fluorination led to stronger XBs between pyridine and iodobenzene derivatives.^23^ Following this principle, the test molecules were selected to generate various degrees of XB with the nitroxide polarizing agent. Remarkably, when using the radical polarizing agent 4-oxo-2,2,6,6-tetramethylpiperidin-1-oxyl (4-oxo-TEMPO), OE-DNP enhancements of the iodinated and brominated carbons of iodobenzene and bromobenzene can be boosted by about 10-fold upon chemical functionalization, corresponding to 100-fold reduction in experiment time for achieving the same signal-to-noise level. Computational analysis revealed strong correlations between OE-DNP performance and XB strength, allowing the establishment of an XB/OE-DNP relationship.

**Scheme 1:**
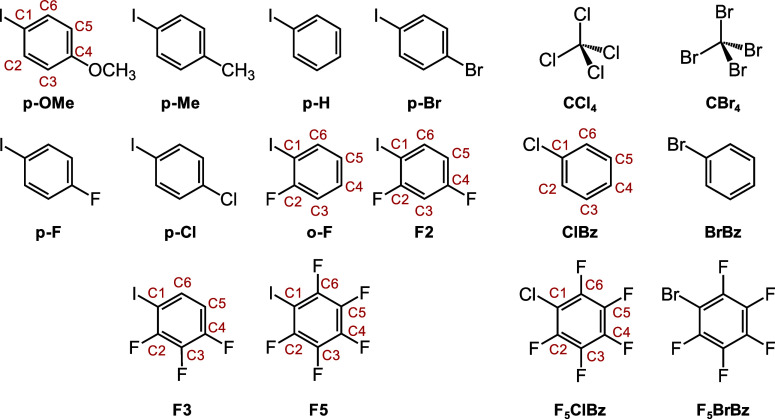
Structures and Abbreviations of Investigated Analytes Red labels indicate carbon numbering, with *p*-Me, *p*-H, *p*-Br, *p*-F, and *p*-Cl following *p*-OMe (iodinated carbon as C1 and *para* carbon as C4). BrBz and F_5_BrBz follow the Cl counterparts (brominated carbon as C1).

^13^C OE-DNP experiments were performed at 9.4 T external magnetic field using a 263 GHz high-power gyrotron and a commercial liquid-state NMR instrument equipped with a novel DNP probe head.^15,21^ Spectra were collected on ∼20 μL of deoxygenated analyte solutions (0.6 mol/L for better signal-to-noise or 0.3 mol/L when solubility is limited) doped with 0.025 mol/L 4-oxo-TEMPO-^15^N-*d*_16_ (TN), a polarizing agent producing a strong ^13^C DNP effect. Although two different analyte concentrations were used, measurements on iodobenzene showed a negligible concentration dependence for ε(^13^C) (Figure S1 and Table S1). We chose CCl_4_ as the solvent due to favorable physicochemical properties (polarity, viscosity, and melting point) for microwave irradiation and penetration. To rule out the solvent effect possibly arising from additional XB formation, we further validated our results in cyclopentane (Table S2). Enhancements were evaluated as the ratio of the integrated NMR peak areas of spectra collected on the same sample under DNP conditions and thermal Boltzmann equilibrium scaled by the number of scans. To correlate OE-DNP performance with molecular properties, we examined the carbon-13 coupling factors, ξ(^13^C), which correct the enhancements for the influences of instrument design, sample composition, etc.^24^ ξ(^13^C) is related to ε(^13^C) through the following equation:
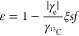
1where γ_e_ and γ_^13^C_ are electron and carbon gyromagnetic ratios (|γ_e_|/γ_^13^C_ = 2617). The saturation factor *s* describes the degree of polarizing agent electron spin saturation and is related to the polarizing agent electron spin relaxation time and the microwave field strength.^25,26^ Our previous investigation identified *s* ≈ 0.3 for dilute CCl_4_ solutions doped with 0.025 mol/L TN under similar experimental conditions.^15^ The leakage factor *f* describes the degree of paramagnetic nuclear relaxation and depends on the polarizing agent concentration. *f* can be calculated from *f* = 1 – *T*_1n_/*T*_1n_^0^, where *T*_1n_ and *T*_1n_^0^ are the paramagnetic and diamagnetic nuclear spin–lattice relaxation times. For this, we separately prepared deoxygenated and otherwise identical analyte solutions containing or excluding the polarizing agent. *T*_1n_ and *T*_1n_^0^ were measured using the standard inversion–recovery sequence under thermal population and extracted using monoexponential fits.

Figure 1 displays representative ^13^C NMR spectra collected under OE-DNP and thermal equilibrium conditions for *p*-OMe, *p*-Br, *p*-Cl, F2, F3, and F5 at natural ^13^C abundance. For compounds investigated here (other spectra are reported in Figures S1 and S2), positive DNP signals corresponding to scalar-dominant OE-DNP are always observed for iodinated, brominated, chlorinated, and protonated carbons. Such halogenated and protonated carbon moieties have been shown to form non-covalent interactions with the electron-rich nitroxide radical moieties based on experimental and computational studies.^18,20,27,28^ Signals of fluorinated and quaternary carbons, in contrast, are often quenched under OE-DNP, suggesting a cancellation between scalar and dipolar mechanisms. These agree with previous observations and support the hypothesis that OE-DNP is promoted by analyte–polarizing agent halogen bonding.^12,15^ For the iodobenzene derivatives, enhancements of the iodinated carbons, ε(^13^C_I_), are the most sensitive to aromatic chemical functionalization (Table 1). ε(^13^C_I_) increases systematically from ε = 12 ± 1 for *p*-OMe to ε = 130 ± 13 for F5, as C_I_ experiences stronger electron-withdrawing effect (electron-withdrawing ability: −Me, −OMe < −H < −Br, −Cl < −F < multiple −F based on literature studies;^29,30^ also see results below). In contrast, ε(^13^C) varies by only ∼3-fold for the protonated carbons. For aromatic compounds, brominated and chlorinated carbons have much smaller enhancements than their iodinated counterparts under similar chemical environments (Table 2). For instance, ε(^13^C_X_) (X = Br, Cl) remains around 2–3 ± 1 for BrBz, ClBz, *p*-Br, and *p*-Cl. Although pentafluorination boosts ε(^13^C_Br_) also by about 10-fold for bromobenzene, a value of ε(^13^C_Br_) = 20 ± 2 for F_5_BrBz is about 6 times smaller than ε(^13^C_I_) for F5. Moreover, pentafluorination produced no effect on ε(^13^C_Cl_) of ClBz, which remains at 3 for F_5_ClBz. Finally, similar to the previous report,^12^ we observed large ε(^13^C) for CCl_4_ (ε = 121 ± 10) and CBr_4_ (ε = 180 ± 18). While the values are smaller in our case due to lower electronic saturation,^12,15^ the ε(CBr_4_)/ε(CCl_4_) ratio agrees well with the literature report (1.5 here, 1.4 in ref (12)).

**Figure 1:**
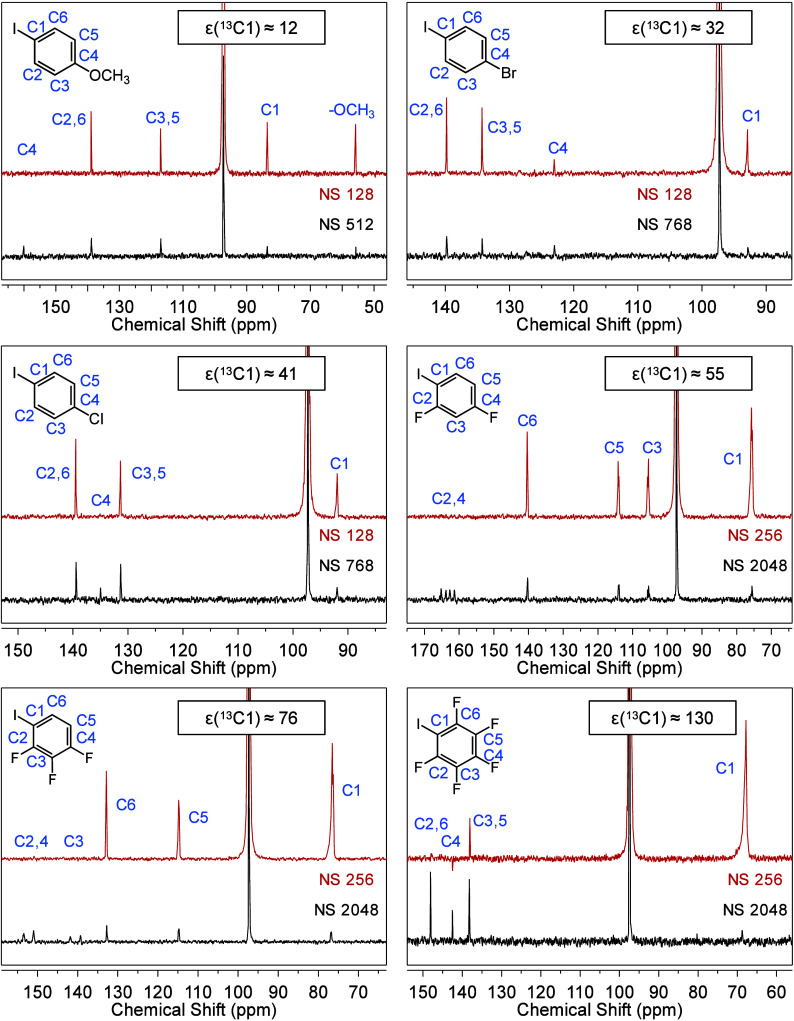
^13^C NMR spectra of selected iodinated compounds in CCl_4_ collected at 9.4 T under OE-DNP (red) and thermal equilibrium (black) conditions. The intense solvent signal at ∼97 ppm is partially cut for better presentation of the analyte peaks. See experimental methods for sample composition and NMR parameters. NS stands for number of scans.

**Table 1:** ^13^C OE-DNP Enhancements at 9.4 T, Leakage Factors, and Coupling Factors of Selected Iodobenzene Derivatives (±10% Error for ε; 5–10% Error for *f*)

analyte	ε(^13^C_I_)	ε(^13^C2)	ε(^13^C3)	ε(^13^C4)	ε(^13^C5)	ε(^13^C6)	*f*(^13^C_I_)	ξ(^13^C_I_)
*p*-OMe	12	7	6	–a	6	7	0.81	–0.017
*p*-Me	17	8	7	–	7	8	0.84	–0.024
*p*-H	28	11	11	9	11	11	0.85	–0.040
*p*-Br	32	9	14	3	14	9	0.82	–0.048
*p*-F	35	13	13	–	13	13	0.86	–0.050
*p*-Cl	41	16	13	3	13	16	0.80	–0.063
o-F	44	–	12	8	9	10	0.91	–0.060
F2	55	–	20	–	17	15	0.88	–0.078
F3	76	–	–	–	16	14	0.88	–0.105
F5	130	–	–	–	–	–	0.97	–0.17

a |ε| < 1 under DNP conditions.

**Table 2:** ^13^C OE-DNP Enhancements at 9.4 T, Leakage Factors, and Coupling Factors of Selected Brominated and Chlorinated Compounds^a^

analyte	ε(^13^C_X_)	*f*(^13^C_X_)	ξ(^13^C_X_)
ClBz	2	0.90	–0.001
BrBz	3	0.57	–0.004
F_5_ClBz	3	0.95	–0.003
F_5_BrBz	20	0.85	–0.028
CCl_4_	121	0.97	–0.16
CBr_4_	180	0.53	–0.43

a X = Br, Cl. Values of ε(^13^C_X_) for ClBz and BrBz were taken from ref (15).

Tables 1 and 2 record *f*(^13^C_X_) and ξ(^13^C_X_) (X = heaviest halogen). Notably, *f*(^13^C_X_) (X = I, Cl) falls in the range of 0.8–0.97. Brominated carbons, however, have much lower *f*(^13^C_Br_) (∼0.55) due to fast diamagnetic *T*_1_ relaxation. This is a result of scalar relaxation between ^13^C and ^79^Br (natural abundance ∼ 50.7%), which are two isotopes with similar γ (γ_^13^C_ = 6.73 × 10^7^ rad/Ts, γ_^79^Br_ = 6.70 × 10^7^ rad/Ts).^31^ In terms of the coupling factor, ξ(^13^C_X_) (X = I, Br, Cl) is negative in all cases, again reflecting scalar-dominant OE-DNP effects. The value for CCl_4_, ξ(^13^C) = −0.16, agrees well with the literature value obtained from an alternative instrument design (−0.17 in ref (12)). For chemically similar systems, |ξ(^13^C_I_)| is generally larger than |ξ(^13^C_X_)| (X = Br, Cl). Functionalization with electron-withdrawing groups promotes |ξ(^13^C_X_)| for iodinated and brominated aromatics but not for chlorinated ones. These results point to a major but complex role of XB in generating the ^13^C OE-DNP for carbons directly bonded to the halogens.

To investigate potential polarizing agent–analyte XB interactions, we performed a computational analysis of the analytes and their TN complexes. We first focused on the iodobenzene derivatives, which experience a systematic increase in OE-DNP enhancements. Scalar OE-DNP requires spin density transfer from the polarizing agent unpaired electron spin to the observed nuclei—here between the halogenated carbons C_X_ and aminoxyl oxygen O_N_. This can be induced by formation of polarizing agent–analyte transient complexes, where the electronic spin density on C_X_ is polarized by the radical, according to previous computational studies.^13,32,33^ Intermolecular interaction is influenced by the molecular electrostatic potential (ESP, *V*_s_) distribution, where sites with positive and negative ESP tend to interact.^34^ Here ESP isosurface analyses for the polarizing agent and the halogenated compounds were performed based on DFT-optimized structures in CCl_4_ (*vide infra*) and electronic wavefunction analysis (also see the Supporting Information). For TN, radical and carbonyl oxygens possess strongly negative ESP minima (*V*_s,min_ ≈ −35 kcal/mol; Figure 2a), similar to literature reports on structurally related nitroxide radicals.^35^ Regardless of chemical functionalization, three electropositive regions can be identified for the iodobenzene derivatives (see Figure 2a for F3 as an example; for other compounds, see Figure S3): the iodine σ hole (ESP maximum (*V*_s,max_) = +30.11 kcal/mol for F3), hydrogen bonding sites of the aromatic protons (*V*_s,max_ = +30.29 and +32.11 kcal/mol for F3), and the aromatic π system (*V*_s,max_ = +6.61 kcal/mol for F3). Based on these considerations, we studied four types of polarizing agent–analyte complexes: those stabilized by the radical–analyte halogen bond (O_N_···I, Figure 2b), an alternative XB involving the polarizing agent carbonyl group (O_C_···I, Figure 2c), a hydrogen bond (O_N_···H, Figure 2d), and a polarizing agent···π interaction (PA···π, Figure 2e). Initial geometries for optimization were set to favor these interactions. We further considered five to eight local minima on the energy surface of each type of polarizing agent–analyte complex (Figures S4–S13). Additionally, only the chair conformer is considered for TN, as the TN ^15^N hyperfine constants calculated from such a conformer agree better with experimental observations (*vide infra*).

**Figure 2:**
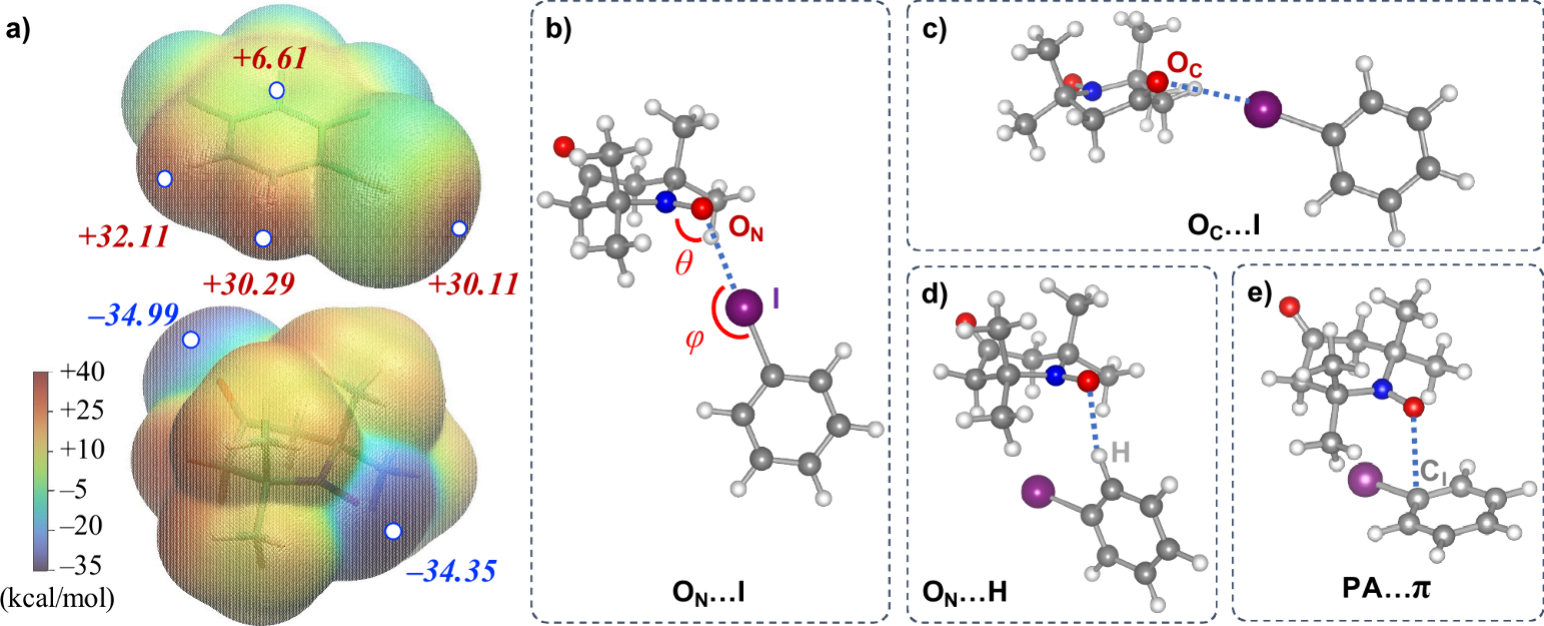
(a) Electrostatic potential isosurfaces (0.001 au) for F3 and TN. Red and blue numbers indicate local ESP maxima and minima, respectively, marked by the white dots. (b–d) Four types of interactions between TN and iodobenzene derivatives (geometries optimized at the M06-2X/aug-cc-pVTZ level), stabilized by the O_N_···I halogen bond (b), O_C_···I halogen bond (c), O_N_···H hydrogen bond (d), and polarizing agent···π interaction (e). Blue dashed lines indicate pathways of intermolecular interaction. θ and φ are the N–O_N_···I and C_I_–I···O_N_ angles described in the text.

For DFT analysis, complex geometries were optimized at the unrestricted M06-2X/aug-cc-pVTZ (-PP for I and Br) levels of theory, which are known to describe non-covalent interactions.^36^ Solvent dielectric effects were described by the SMD model.^37^ The results of the study were further verified by analyzing geometries optimized with a different hybrid functional, B3LYP-D3.^38^ All optimized complexes stabilized by the O_N_···I interaction possess bond angles θ(N–O_N_···I) = 110–140° and φ(C_I_–I···O_N_) ≈ 180° (Figure S14 and Tables S3–S12). Such directionality, especially the strong linearity of C_I_–I···O_N_, is characteristic of XBs according to previous crystallographic and computational studies, where θ(N–O_N_···I) = 120–145° and φ(C_I_–I···O_N_) = 170–180° have been observed for cocrystals and solvated complexes of iodobenzene and nitroxide derivatives.^19,20,23,27,39^ This is a result of interaction between the C–I bond σ hole and the oxygen-based radical spin density (Figure S15). Liquid-state OE-DNP involves molecular motion,^24^ and all these complex configurations may contribute to the observed enhancement. Therefore, to compute physical parameters of the complexes, we consider the averaged quantities of individual geometries weighted by their Gibbs free energies:
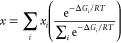
2where *x* is the physical parameter of interest, Δ*G*_*i*_ is the relative Gibbs free energy of geometry *i*, *R* is the ideal gas constant, and *T* is the experimental temperature of 300 K. The same weighting approach has been applied for studying the role of hydrogen bonding in solution OE-DNP at low magnetic field.^28^

For halogen-bonded complexes involving the same XB acceptor, here TN, stronger XB corresponds to more positive ESP maxima at the σ hole of the carbon–iodine bond, *V*_s,max_(CI), shorter bond length *d*(O_N_···I), and more negative binding energy *E*(XB), which can be calculated from the single-point energies of the complexes and their constituents:^20^
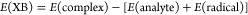
3From *p*-OMe to F5, the average *d*(O_N_···I) decreases while the average |*E*(XB)| and *V*_s,max_(CI) increase (Figure 3 and Table S13), with up to 14%, 2%, or 0.5% variations between individual geometries of the same iodobenzene derivative based on calculations with at the M06-2X/aug-cc-pVTZ level. These observations are consistent with increasing polarizing agent–analyte XB strength upon functionalization with more electron-withdrawing groups. Importantly, these parameters show linear correlations (*r*^2^ ≥ 0.96) with ξ(^13^C_I_) (Figure 3a–c), pointing to the active role of XB in the OE-DNP performance of these systems. A similar linear correlation between ξ(^13^C_I_) and |*E*(XB)| was observed when the latter were evaluated using the domain-based local-pair natural orbital coupled-cluster singles and doubles (DLPNO–CCSD) method (Figure S16), which is more costly but often regarded as the “gold standard” for calculating electronic properties.^40^

**Figure 3:**
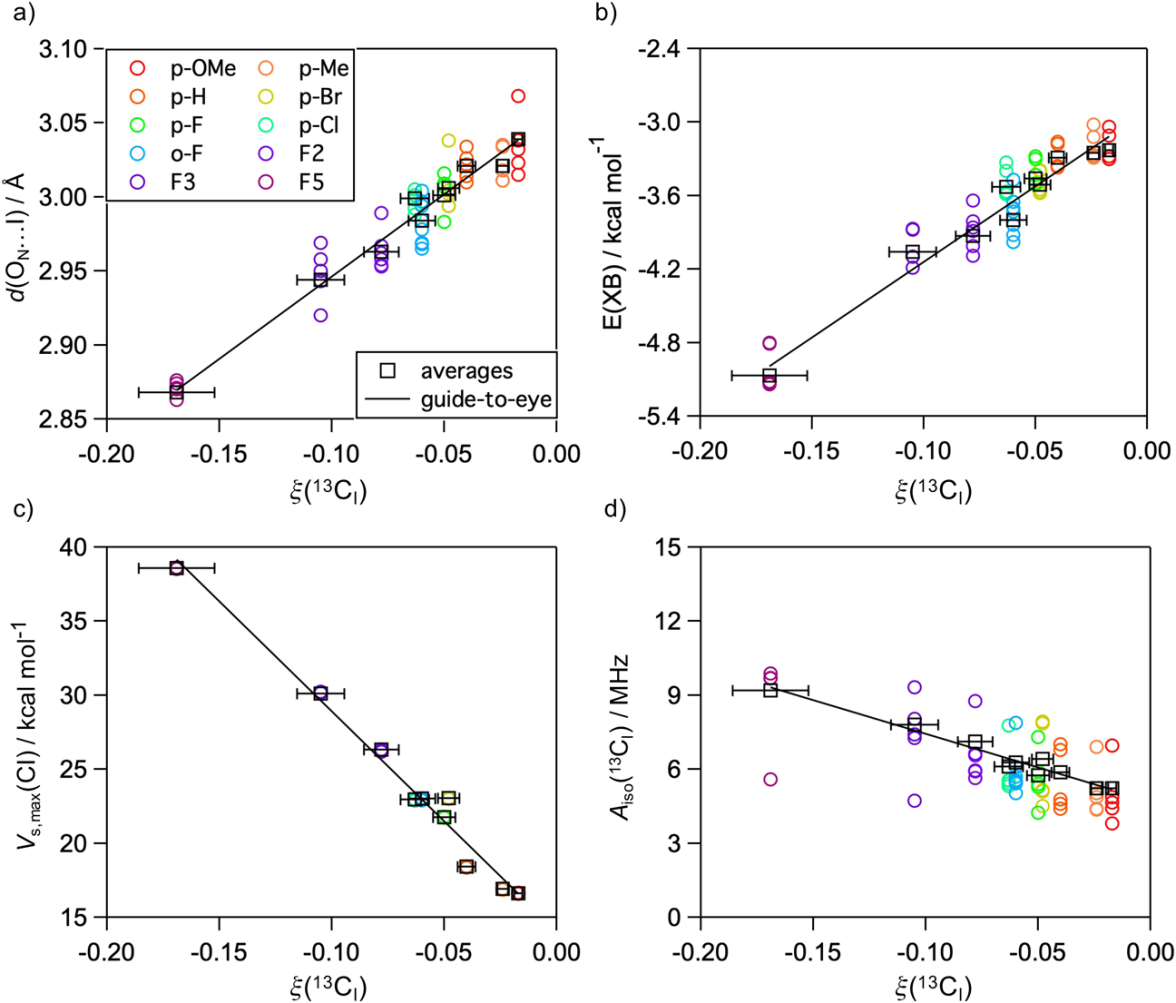
Correlations between ξ(^13^C_I_) and *d*(O_N_···I) (a), *E*(XB) (b), *V*_s,max_(CI) (c), and *A*_iso_(^13^C_I_) (d). Colored circles represent values of individual geometries, and black squares represent averaged values according to eq 2. Black solid lines are guides to the eye for the averaged data, with *r*^2^ = 0.98 (a), *r*^2^ = 0.96 (b), *r*^2^ = 0.98 (c), and *r*^2^ = 0.96 (d). Geometry optimizations and *E*(XB) calculations were performed at the M06-2X/aug-cc-pVTZ level. *A*_iso_(^13^C_I_) were evaluated at the B3LYP-D3/aug-cc-pVTZ-J level.

For scalar-dominant OE-DNP at high magnetic fields induced by spin-1/2 monoradical, ξ(^13^C) is approximately determined by the ratio of the electron–nuclear zero-quantum scalar (*w*_0_^s^) and single-quantum dipolar (*w*_1_^d^) relaxation rates:^24^
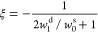
4where *w*_0_^s^ is related to the squared time average of the carbon-13 isotropic hyperfine constant, *A*_iso_(^13^C), according to the pulse model described in ref (32):
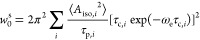
5where *i* represents a pathway of analyte–polarizing agent complex formation, τ_p,*i*_^–1^ is the formation frequency, τ_c,*i*_ is the complex lifetime, and ω_e_ is the electronic Larmor frequency. When other parameters are fixed, larger *A*_iso_(^13^C) should lead to larger *w*_0_^s^ and |ξ(^13^C)| and thus a stronger OE-DNP effect. To examine whether XB formation is correlated with a large *A*_iso_(^13^C_I_), we calculated isotropic hyperfine constants of the iodinated ^13^C for the optimized complexes discussed above using computational methods at DFT and coupled-cluster levels of theory. The computational methods were validated against the experimental ^15^N hyperfine constants of TN measured using an X-band continuous-wave electron paramagnetic resonance spectrometer, with dispersion-corrected B3LYP-D3/aug-cc-pVTZ-J giving the best match (Table S14). *A*_iso_(^13^C_I_) values computed using B3LYP-D3 are subsequently reported in Figure 3d, and those for other methods are shown in Figure S17. In contrast, ^15^N hyperfine constants of ∼41 MHz were obtained for complexes with twisted polarizing agent conformation (Figure S18), which were subsequently omitted in the following discussion due to large deviation from experimentally observed values. For each iodobenzene derivative, up to 40% variation in *A*_iso_(^13^C_I_) is observed between individual geometries, which is a result of difference in binding geometries and electronic properties. The average *A*_iso_(^13^C_I_) increases from 5.23 MHz for *p*-OMe to 9.19 MHz for F5 and could be interpreted as correlated (*r*^2^ = 0.96) with ξ(^13^C_I_) (Figure 3d). While such correlation should not be linear according to eqs 4 and 5, the observed linearity might be an approximation of the complex influence of *w*_0_^s^ and *w*_1_^d^. Note that such correlation persists when obtained with different computational methods, despite the fact that the absolute *A*_iso_(^13^C_I_) values calculated with DFT are on average ∼2.5 MHz larger than those obtained from DLPNO–CCSD (Figure S17 and Table S15). The increase in *A*_iso_(^13^C_I_) is reflected in the increasing Löwdin spin population,^41^ ρ(^13^C_I_), and s-orbital character of the iodinated carbons, with average values increasing from 0.54% to 0.77% and from 13.6% to 15.7%, respectively (Table S13). Increases in ρ(^13^C_I_) are further accompanied by a decrease in ρ(^15^NO), defined as ρ(^15^N) + ρ(O_N_) for the polarizing agent, from 87.7% to 86.8%. The spin densities evolve linearly with ξ(^13^C_I_) (Figure S19). This is consistent with the expectation that the hyperfine interaction and subsequently the OE-DNP effect are induced by a polarizing agent-to-analyte spin polarization mechanism.^24,42^ The aforementioned observations remained valid when *A*_iso_(^13^C_I_) was calculated from geometries optimized at the B3LYP-D3 level (Table S16 and Figure S20). Further geometry analysis for these complexes indicated dihedral angles ζ close to 90° or 270° (Figure S21), which are consistent with an orbital overlap along the nitroxide oxygen p_*z*_ orbital. This orientation was found to facilitate strong analyte–polarizing agent hyperfine coupling in water hydrogen-bonded to nonplanar nitroxide radicals.^43^ These results suggest that analyte–polarizing agent geometries favoring XB also facilitate the isotropic hyperfine interactions required for OE-DNP. Additionally, it is worth noting that *A*_iso_(^13^C_I_) is not determined by a single geometric parameter but rather by a combination of θ, φ, and the XB-associated dihedral angle ζ (Figure S22).

For analyte–polarizing agent interactions potentially competing with that through the O_N_···I halogen bond (Figure 2c–e), much weaker *A*_iso_(^13^C_I_) values were observed, from *A*_iso_(^13^C_I_) ≈ 0.43–0.53 MHz for the O_N_···H interaction to |*A*_iso_(^13^C_I_)| ≈ 0.09–0.24 MHz for PA···π to negligible |*A*_iso_(^13^C_I_)| ≤ 0.02 MHz for O_C_···I (Table S17). The nature of these interactions is confirmed by geometry and orbital analyses (Table S18 and Scheme S1), including the strong linearity of O_C_···I (Figure S23), the <90° angle connection for O_N_···H (Figure S24), and the right-angle connection combined with the involvement of carbon p orbitals for PA···π (Figures S25 and S26). The weaker hyperfine interaction is a result of unfavorable spin polarization transfer, as demonstrated by the lower ρ(^13^C_I_) compared to those observed for O_N_···I, which offsets the superior s-orbital characters observed for some geometries (Figures S19b and S27). Interestingly, no correlation was found between ξ(^13^C_I_) and the *A*_iso_(^13^C_I_) values obtained from these interactions (Figure S28). Additionally, the ^13^C chemical shift differences between the DNP and Boltzmann spectra, Δδ, reflect the analyte paramagnetic shifts, partially compensated due to saturation of the TN EPR transition under OE-DNP.^44^ For fast-tumbling molecules in the liquid state, the paramagnetic shifts of ^13^C are linearly correlated with the *A*_iso_(^13^C) averaged over time and polarizing agent–analyte interaction pathways.^28,45^ For the iodobenzene derivatives, such correlation was observed for *A*_iso_(^13^C_I_) calculated from the O_N_···I interaction but not for *A*_iso_(^13^C_I_) for the O_C_···I, O_N_···H, and PA···π interactions (see Figure S29 and related discussion). Altogether, these results for the iodobenzene derivatives corroborate the importance of the halogen bond in influencing the polarizing agent–analyte interaction and generating a large OE-DNP effect. Of equal importance, only those interactions with suitable geometry and efficient spin polarization mechanism are actually responsible for the observed correlation between XB and DNP performance.

On the other hand, analysis of the brominated and chlorinated compounds emphasizes that the predicted *A*_iso_(^13^C_I_) and XB properties do not alone explain the huge differences in the observed OE-DNP effects. Similar to the case of the iodobenzene derivatives, we analyzed multiple geometries stabilized by the O_N_···X halogen bond (X = Br, Cl), i.e., with C–X oriented toward the TN radical site (Figures S30–S35 and Tables S19–S24). DFT revealed that optimized CX_4_–TN complexes possess strongly directional interactions (θ ≈ 115–141°, φ ≈ 180°). Complexes of the aromatics, however, have larger deviations from geometries that facilitate efficient XB and hyperfine interaction (θ ≈ 90–140°, φ ≈ 140–180°). Moreover, whereas the increase in the XB strength still corresponds to an increase in *A*_iso_(^13^C_X_) (X = Br, Cl) calculated for XB-stabilized complexes (Figure S36 and Table S25), they no longer translate to a monotonic increase in |ξ(^13^C_X_)| or ε(^13^C_X_) (Figure 4). For instance, ξ(^13^C_X_) remains invariant for ClBz, BrBz, and F_5_ClBz despite a 1.3 kcal/mol increase in *E*(XB) and a 16.0 kcal/mol increase in *V*_s,max_(XB). Similar trends were observed for OE-DNP experiments performed with samples with cyclopentane as the solvent (Figure S37).

**Figure 4:**
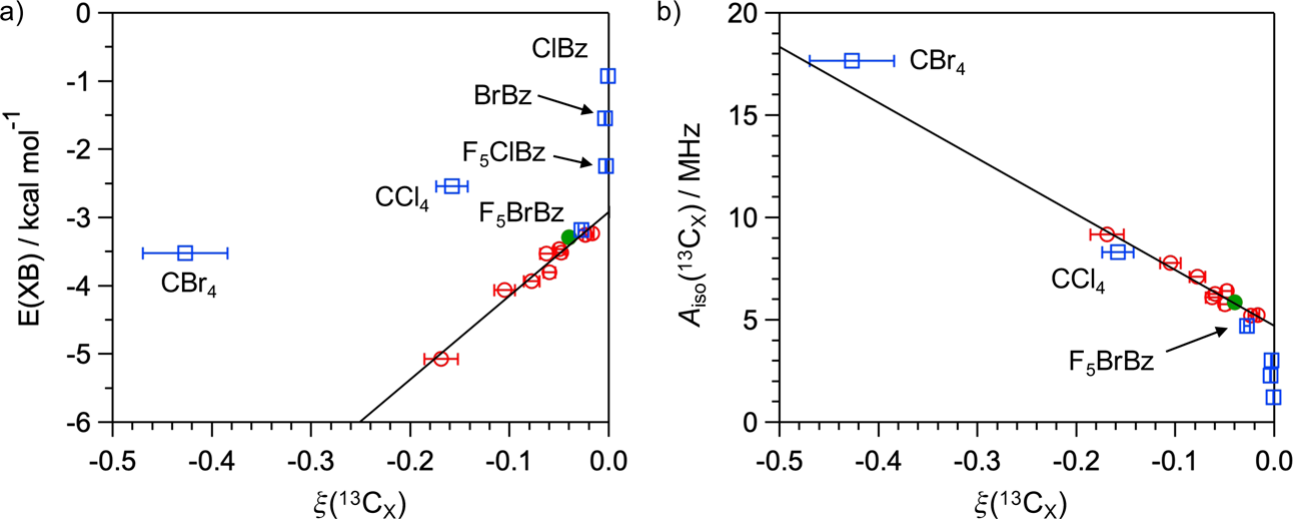
Correlations between (a) *E*(XB) or (b) *A*_iso_(^13^C_X_) and ξ(^13^C_X_) calculated for complexes stabilized by the O_N_···X halogen bond. Red open circles represent iodinated compounds. Blue squares represent brominated and chlorinated compounds. Iodobenzene is marked by green solid circles for clarity. Black lines are guides for the eye presented in Figure 3. *E*(XB) and *A*_iso_(^13^C_X_) were obtained from calculations with the M06-2X and B3LYP-D3 functionals, respectively.

Several factors could contribute to the complex correlation between computed XB properties and the OE-DNP performance. As discussed above, ^13^C OE-DNP involves two temporal aspects: the formation frequency (τ_p,*i*_^–1^) and lifetime (τ_c,*i*_) of the analyte–polarizing agent complex. For the halogenated molecules studied here, DFT and electronic wavefunction analyses already suggest potential variation of these factors. First, molecules with large ε(^13^C_X_) coincide with those with *V*_s,max_ located at the XB, such as F5, CCl_4_, and CBr_4_ (Figure S3, S38). The difference in ESP distribution possibly explains the invariant ε(^13^C_Cl_) of ClBz upon pentafluorination, as F_5_ClBz is the only pentafluorinated compound with *V*_s,max_ outside the XB. Such ESP distribution analysis agrees with the previous observation from NMR chemical shift titration that F_5_ClBz prefers π interaction over XB.^46^ Meanwhile, for CX_4_, interaction of TN with low *V*_s_ regions, such as the center of the −CX_3_ cone, can still induce non-negligible *A*_iso_(^13^C) (∼1.8–3 MHz; Figure S39). Such interactions have been computationally identified in complexes of CCl_4_ and oxygen-based XB acceptors.^47^ Additionally, the relative surface area of the XB σ hole of the analyte, as characterized by positive *V*_s_, increases with functionalization with more electron-withdrawing groups and become largest for carbon tetrahalides (Table S26). These likely cause variations in the dynamics and probability of polarizing agent–analyte interaction pathways. Such variation is further supported by the different Δδ(^13^C_X_)–*A*_iso_(^13^C_X_) dependence of the brominated and chlorinated analytes compared to the iodinated ones (Figure S40).

Finally, the analyte–polarizing agent complexes possess various motions with frequencies matching the time scale suited for OE-DNP. Under 9.4 T, where the electronic Larmor frequency is 263 GHz, optimal OE-DNP requires molecular motion on the time scale of ∼0.6 ps (i.e., ω_e_^–1^), corresponding to ν ∼ 8.8 cm^–1^. Interestingly, numerical vibrational frequency calculations on the complexes revealed low-energy hybrid bending modes of the XB around this frequency (Figures S41 and S42 and Table S27), which further lead to changes in *A*_iso_(^13^C_X_). For instance, vibrations of 2.5–20.8 cm^–1^ alter *A*_iso_(^13^C_X_) of the complexes by <0.01 to 5.4 MHz (Figure S43). A previous study on chloroform revealed that efficient OE-DNP at high field is induced by not only formation–dissociation but also subpicosecond processes of the polarizing agent–analyte transient complex.^32^ A similar effect could be induced by the above-mentioned vibrational modes. Additionally, it is worth noting that experimental study of such modes is rare. Infrared spectroscopic studies have identified the C–I stretch to be around 400 cm^–1^ and influenced by XB strength.^48^ The lowest XB vibrational modes observed so far are about 60–70 cm^–1^ for azopyridine–tetrafluorodiiodobenzene in the solid state according to Raman spectroscopy.^49^ Although optical spectroscopy has revealed pico- to subpicosecond dynamics in pure CCl_4_ and benzene, either assigned to molecular collisions^50^ or collective modes,^51^ such analyses have not been applied to OE-DNP systems. Thorough understanding of processes on this time scale will require suitable spectroscopic methods, which is the topic of ongoing investigations.

In conclusion, we analyzed the DNP performance and halogen bond properties of representative iodinated, brominated, and chlorinated compounds. We established, for the first time, that strong XB can facilitate a strong Overhauser DNP effect for the carbon atoms involved. This is a result of an efficient XB-mediated polarizing agent-to-analyte spin polarization transfer. This effect enables up to 10-fold boosts in the OE-DNP performance through analyte chemical functionalization. Potentially, our results open up new ways of fine-tuning DNP performance by controlling intermolecular interactions, a useful aspect for designing hyperpolarized molecular probes and labels.^6,8^ Additionally, we envision the implementation of Overhauser DNP in studying halogen bonding itself, which plays unique roles in various applications, including organocatalysis, molecular recognition, bioinhibitor design, and crystal engineering.^52−55^

## Data Availability

Source data is available at the Göttinger Research Online Database under accession link https://doi.org/10.25625/RTPVQI.

## Supporting Information Available

Experimental methods, computational details, additional analyses of geometric and electronic properties, and validation in another solvent and with other computational methods (PDF)

## References

[ref1] Felli I. C.; Pierattelli R. ^13^C Direct Detected NMR for Challenging Systems. Chem. Rev. 2022, 122 (10), 9468–9496. 10.1021/acs.chemrev.1c00871. 35025504 PMC9136920

[ref2] Griesinger C.; Bennati M.; Vieth H. M.; Luchinat C.; Parigi G.; Höfer P.; Engelke F.; Glaser S. J.; Denysenkov V.; Prisner T. F. Dynamic Nuclear Polarization at High Magnetic Fields in Liquids. Prog. Nucl. Magn. Reson. Spectrosc. 2012, 64, 4–28. 10.1016/j.pnmrs.2011.10.002. 22578315

[ref3] Ardenkjaer-Larsen J. H.; Boebinger G. S.; Comment A.; Duckett S.; Edison A. S.; Engelke F.; Griesinger C.; Griffin R. G.; Hilty C.; Maeda H.; et al. Facing and Overcoming Sensitivity Challenges in Biomolecular NMR Spectroscopy. Angew. Chem., Int. Ed. 2015, 54 (32), 9162–9185. 10.1002/anie.201410653. PMC4943876 26136394

[ref4] Buntkowsky G.; Theiss F.; Lins J.; Miloslavina Y. A.; Wienands L.; Kiryutin A.; Yurkovskaya A. Recent Advances in the Application of Parahydrogen in Catalysis and Biochemistry. RSC Adv. 2022, 12 (20), 12477–12506. 10.1039/D2RA01346K. 35480380 PMC9039419

[ref5] Wang Y.; Hilty C. Determination of Ligand Binding Epitope Structures Using Polarization Transfer from Hyperpolarized Ligands. J. Med. Chem. 2019, 62 (5), 2419–2427. 10.1021/acs.jmedchem.8b01711. 30715877

[ref6] Bütikofer M.; Stadler G. R.; Kadavath H.; Cadalbert R.; Torres F.; Riek R. Rapid Protein-Ligand Affinity Determination by Photoinduced Hyperpolarized NMR. J. Am. Chem. Soc. 2024, 146 (26), 17974–17985. 10.1021/jacs.4c04000. 38957136 PMC11228983

[ref7] Meier S.; Jensen P. R.; Karlsson M.; Lerche M. H. Hyperpolarized NMR Probes for Biological Assays. Sensors 2014, 14 (1), 1576–1597. 10.3390/s140101576. 24441771 PMC3926627

[ref8] Angelovski G.; Tickner B. J.; Wang G. Opportunities and Challenges with Hyperpolarized Bioresponsive Probes for Functional Imaging Using Magnetic Resonance. Nat. Chem. 2023, 15 (6), 755–763. 10.1038/s41557-023-01211-3. 37264100

[ref9] Lesage A.; Lelli M.; Gajan D.; Caporini M. A.; Vitzthum V.; Miéville P.; Alauzun J.; Roussey A.; Thieuleux C.; Mehdi A.; et al. Surface Enhanced NMR Spectroscopy by Dynamic Nuclear Polarization. J. Am. Chem. Soc. 2010, 132 (44), 15459–15461. 10.1021/ja104771z. 20831165

[ref10] Okuno Y.; Mecha M. F.; Yang H.; Zhu L.; Fry C. G.; Cavagnero S. Laser- And Cryogenic Probe-Assisted NMR Enables Hypersensitive Analysis of Biomolecules at Submicromolar Concentration. Proc. Natl. Acad. Sci. U. S. A. 2019, 116 (24), 11602–11611. 10.1073/pnas.1820573116. 31142651 PMC6575578

[ref11] Dubroca T.; Wi S.; van Tol J.; Frydman L.; Hill S. Large Volume Liquid State Scalar Overhauser Dynamic Nuclear Polarization at High Magnetic Field. Phys. Chem. Chem. Phys. 2019, 21 (38), 21200–21204. 10.1039/C9CP02997D. 31310269

[ref12] Orlando T.; Dervişoğlu R.; Levien M.; Tkach I.; Prisner T. F.; Andreas L. B.; Denysenkov V. P.; Bennati M. Dynamic Nuclear Polarization of ^13^C Nuclei in the Liquid State over a 10 T Field Range. Angew. Chem., Int. Ed. 2019, 58 (5), 1402–1406. 10.1002/anie.201811892. 30485626

[ref13] Dai D.; Wang X.; Liu Y.; Yang X.; Glaubitz C.; Denysenkov V.; He X.; Prisner T.; Mao J. Room-Temperature Dynamic Nuclear Polarization Enhanced NMR Spectroscopy of Small Biological Molecules in Water. Nat. Commun. 2021, 12 (1), 6880 10.1038/s41467-021-27067-0. 34824218 PMC8616939

[ref14] Rao Y.; Venkatesh A.; Moutzouri P.; Emsley L. ^1^H Hyperpolarization of Solutions by Overhauser Dynamic Nuclear Polarization with ^13^C–^1^H Polarization Transfer. J. Phys. Chem. Lett. 2022, 13 (33), 7749–7755. 10.1021/acs.jpclett.2c01956. 35969266 PMC9421900

[ref15] Levien M.; Yang L.; van der Ham A.; Reinhard M.; John M.; Purea A.; Ganz J.; Marquardsen T.; Tkach I.; Orlando T.; et al. Overhauser Enhanced Liquid State Nuclear Magnetic Resonance Spectroscopy in One and Two Dimensions. Nat. Commun. 2024, 15 (1), 5904 10.1038/s41467-024-50265-5. 39003303 PMC11246421

[ref16] Liu G.; Levien M.; Karschin N.; Parigi G.; Luchinat C.; Bennati M. One-Thousand-Fold Enhancement of High Field Liquid Nuclear Magnetic Resonance Signals at Room Temperature. Nat. Chem. 2017, 9 (7), 676–680. 10.1038/nchem.2723. 28644482

[ref17] Cavallo G.; Metrangolo P.; Milani R.; Pilati T.; Priimagi A.; Resnati G.; Terraneo G. The Halogen Bond. Chem. Rev. 2016, 116 (4), 2478–2601. 10.1021/acs.chemrev.5b00484. 26812185 PMC4768247

[ref18] Mugnaini V.; Punta C.; Liantonio R.; Metrangolo P.; Recupero F.; Resnati G.; Pedulli G. F.; Lucarini M. Noncovalent Paramagnetic Complexes: Detection of Halogen Bonding in Solution by ESR Spectroscopy. Tetrahedron Lett. 2006, 47 (19), 3265–3269. 10.1016/j.tetlet.2006.03.033.

[ref19] Davy K. J. P.; McMurtrie J.; Rintoul L.; Bernhardt P. V.; Micallef A. S. Vapour Phase Assembly of a Halogen Bonded Complex of an Isoindoline Nitroxide and 1,2-Diiodotetrafluorobenzene. CrystEngComm 2011, 13 (16), 5062–5070. 10.1039/c1ce05344b.

[ref20] Cimino P.; Pavone M.; Barone V. Halogen Bonds between 2,2,6,6-Tetramethylpiperidine-*N*-oxyl Radical and C_*x*_H_*y*_F_*z*_I Species: DFT Calculations of Physicochemical Properties and Comparison with Hydrogen Bonded Adducts. J. Phys. Chem. A 2007, 111 (34), 8482–8490. 10.1021/jp073567b. 17685598

[ref21] Marquardsen T.; Bennati M.; Tkach I.; Levien M.; Orlando T.; Yang L.; Leavesley A.; Wylde R. DNP Probe Head for High Resolution, Liquid-State NMR. WO 2024/115698, 2024.

[ref22] Riley K. E.; Murray J. S.; Fanfrlík J.; Řezáč J.; Solá R. J.; Concha M. C.; Ramos F. M.; Politzer P. Halogen Bond Tunability I: The Effects of Aromatic Fluorine Substitution on the Strengths of Halogen-Bonding Interactions Involving Chlorine, Bromine, and Iodine. J. Mol. Model. 2011, 17 (12), 3309–3318. 10.1007/s00894-011-1015-6. 21369930

[ref23] Präsang C.; Whitwood A. C.; Bruce D. W. Halogen-Bonded Cocrystals of 4-(*N*,*N*-Dimethylamino)pyridine with Fluorinated Iodobenzenes. Cryst. Growth Des. 2009, 9 (12), 5319–5326. 10.1021/cg900823d.

[ref24] Bennati M.; Orlando T. Overhauser DNP in Liquids on ^13^C Nuclei. eMagRes 2019, 8 (1), 11–18. 10.1002/9780470034590.emrstm1581.

[ref25] Türke M.-T.; Bennati M. Saturation Factor of Nitroxide Radicals in Liquid DNP by Pulsed ELDOR Experiments. Phys. Chem. Chem. Phys. 2011, 13 (9), 3630–3633. 10.1039/c0cp02126a. 21264371

[ref26] Türke M.-T.; Parigi G.; Luchinat C.; Bennati M. Overhauser DNP with ^15^N Labelled Frémy’s Salt at 0.35 T. Phys. Chem. Chem. Phys. 2012, 14 (2), 502–510. 10.1039/C1CP22332A. 22069056

[ref27] Pang X.; Jin W. J. Exploring the Halogen Bond Specific Solvent Effects in Halogenated Solvent Systems by ESR Probe. New J. Chem. 2015, 39 (7), 5477–5483. 10.1039/C5NJ00300H.

[ref28] Wang X.; Isley W. C.; Salido S. I.; Sun Z.; Song L.; Tsai K. H.; Cramer C. J.; Dorn H. C. Optimization and Prediction of the Electron-Nuclear Dipolar and Scalar Interaction in ^1^H and ^13^C Liquid State Dynamic Nuclear Polarization. Chem. Sci. 2015, 6 (11), 6482–6495. 10.1039/C5SC02499D. 30090267 PMC6054052

[ref29] Halogen Bonding in Solution; Huber S., Ed.; Wiley-VCH, 2021. 10.1002/9783527825738.

[ref30] Scheiner S.; Hunter S. Influence of Substituents in the Benzene Ring on the Halogen Bond of Iodobenzene with Ammonia. ChemPhysChem 2022, 23 (6), 1–8. 10.1002/cphc.202200011. 35099849

[ref31] Breitmaier E.; Spohn K.; Berger S. ^13^C Spin-Lattice Relaxation Times and the Mobility of Organic Molecules in Solution. Angew. Chem., Int. Ed. 1975, 14 (3), 144–159. 10.1002/anie.197501441.

[ref32] Orlando T.; Kuprov I.; Hiller M. Theoretical Analysis of Scalar Relaxation in ^13^C-DNP in Liquids. J. Magn. Reson. Open 2022, 10–11, 100040 10.1016/j.jmro.2022.100040.

[ref33] Küçük S. E.; Sezer D. Multiscale Computational Modeling of ^13^C DNP in Liquids. Phys. Chem. Chem. Phys. 2016, 18 (14), 9353–9357. 10.1039/C6CP01028H. 27001446

[ref34] Murray J. S.; Politzer P. Molecular Electrostatic Potentials and Noncovalent Interactions. Wiley Interdiscip. Rev. Comput. Mol. Sci. 2017, 7 (6), 1–10. 10.1002/wcms.1326.

[ref35] Shiroudi A.; Śmiechowski M.; Czub J.; Abdel-Rahman M. A. Computational Analysis of Substituent Effects on Proton Affinity and Gas-Phase Basicity of TEMPO Derivatives and Their Hydrogen Bonding Interactions with Water Molecules. Sci. Rep. 2024, 14 (1), 1–18. 10.1038/s41598-024-58582-x. 38600208 PMC11006853

[ref36] Kozuch S.; Martin J. M. L. Halogen Bonds: Benchmarks and Theoretical Analysis. J. Chem. Theory Comput. 2013, 9 (4), 1918–1931. 10.1021/ct301064t. 26583543

[ref37] Marenich A. V.; Cramer C. J.; Truhlar D. G. Universal Solvation Model Based on Solute Electron Density and on a Continuum Model of the Solvent Defined by the Bulk Dielectric Constant and Atomic Surface Tensions. J. Phys. Chem. B 2009, 113 (18), 6378–6396. 10.1021/jp810292n. 19366259

[ref38] Grimme S.; Antony J.; Ehrlich S.; Krieg H. A Consistent and Accurate Ab Initio Parametrization of Density Functional Dispersion Correction (DFT-D) for the 94 Elements H-Pu. J. Chem. Phys. 2010, 132 (15), 154104 10.1063/1.3382344. 20423165

[ref39] Espallargas G. M.; Recuenco A.; Romero F. M.; Brammer L.; Libri S. One-Dimensional Organization of Free Radicals via Halogen Bonding. CrystEngComm 2012, 14 (20), 6381–6383. 10.1039/c2ce26131f.

[ref40] Saitow M.; Neese F. Accurate Spin-Densities Based on the Domain-Based Local Pair-Natural Orbital Coupled-Cluster Theory. J. Chem. Phys. 2018, 149 (3), 034104 10.1063/1.5027114. 30037259

[ref41] Löwdin P. O. On the Non-Orthogonality Problem Connected with the Use of Atomic Wave Functions in the Theory of Molecules and Crystals. J. Chem. Phys. 1950, 18 (3), 365–375. 10.1063/1.1747632.

[ref42] McConnell H. M. Indirect Hyperfine Interactions in the Paramagnetic Resonance Spectra of Aromatic Free Radicals. J. Chem. Phys. 1956, 24 (4), 764–766. 10.1063/1.1742605.

[ref43] Hecker F.; Fries L.; Hiller M.; Chiesa M.; Bennati M. ^17^O Hyperfine Spectroscopy Reveals Hydration Structure of Nitroxide Radicals in Aqueous Solutions. Angew. Chem., Int. Ed. 2023, 62 (4), 1–6. 10.1002/anie.202213700. PMC10107301 36399425

[ref44] Neugebauer P.; Krummenacker J. G.; Denysenkov V. P.; Parigi G.; Luchinat C.; Prisner T. F. Liquid State DNP of Water at 9.2 T: An Experimental Access to Saturation. Phys. Chem. Chem. Phys. 2013, 15 (16), 6049–6056. 10.1039/c3cp44461a. 23493879

[ref45] Russ J. L.; Gu J.; Tsai K.-H.; Glass T.; Duchamp J. C.; Dorn H. C. Nitroxide/Substrate Weak Hydrogen Bonding: Attitude and Dynamics of Collisions in Solution. J. Am. Chem. Soc. 2007, 129 (22), 7018–7027. 10.1021/ja064632i. 17497854

[ref46] Yan X. Q.; Zhao X. R.; Wang H.; Jin W. J. The Competition of σ-Hole···Cl^–^ and π-Hole···Cl^–^ Bonds between C_6_F_5_X (X = F, Cl, Br, I) and the Chloride Anion and Its Potential Application in Separation Science. J. Phys. Chem. B 2014, 118 (4), 1080–1087. 10.1021/jp4097869. 24405511

[ref47] Wang P.; Zhao N.; Tang Y. Halogen Bonding in the Complexes of CH_3_I and CCl_4_ with Oxygen-Containing Halogen-Bond Acceptors. J. Phys. Chem. A 2017, 121 (26), 5045–5055. 10.1021/acs.jpca.7b04342. 28587451

[ref48] Person W. B.; Humphrey R. E.; Popov A. I. Infrared Spectra of Charge Transfer Complexes. II. Iodine Cyanide Complexes. J. Am. Chem. Soc. 1959, 81 (2), 273–277. 10.1021/ja01511a004.

[ref49] Kalout H.; Boubegtiten-Fezoua Z.; Maurel F.; Hellwig P.; Ferlay S. An Accurate Vibrational Signature in Halogen Bonded Molecular Crystals. Phys. Chem. Chem. Phys. 2022, 24 (24), 15103–15109. 10.1039/D2CP01336C. 35698883

[ref50] Bucaro J. A.; Litovitz T. A. Molecular Motions in CCl_4_: Light Scattering and Infrared Absorption. J. Chem. Phys. 1971, 55 (7), 3585–3588. 10.1063/1.1676617.

[ref51] McMorrow D.; Lotshaw W. T. Evidence for Low-Frequency (≈15 cm^–1^) Collective Modes in Benzene and Pyridine Liquids. Chem. Phys. Lett. 1993, 201 (1–4), 369–376. 10.1016/0009-2614(93)85085-3.

[ref52] Wilcken R.; Zimmermann M. O.; Lange A.; Joerger A. C.; Boeckler F. M. Principles and Applications of Halogen Bonding in Medicinal Chemistry and Chemical Biology. J. Med. Chem. 2013, 56 (4), 1363–1388. 10.1021/jm3012068. 23145854

[ref53] Mukherjee A.; Tothadi S.; Desiraju G. R. Halogen Bonds in Crystal Engineering: Like Hydrogen Bonds yet Different. Acc. Chem. Res. 2014, 47 (8), 2514–2524. 10.1021/ar5001555. 25134974

[ref54] Tepper R.; Schubert U. S. Halogen Bonding in Solution: Anion Recognition, Templated Self-Assembly, and Organocatalysis. Angew. Chem., Int. Ed. 2018, 57 (21), 6004–6016. 10.1002/anie.201707986. 29341377

[ref55] Berger G.; Frangville P.; Meyer F. Halogen Bonding for Molecular Recognition: New Developments in Materials and Biological Sciences. Chem. Commun. 2020, 56 (37), 4970–4981. 10.1039/D0CC00841A. 32297598

