# Supplementary material for: Halogen-Bond-Mediated 13C Overhauser Dynamic Nuclear Polarization at 9.4 T
Source: J Phys Chem Lett. 2025 Apr 28;16(18):4505–14. doi: 10.1021/acs.jpclett.5c00798 (PMC12067439; doi:10.1021/acs.jpclett.5c00798)
Supplement: Supplementary file 1 — jz5c00798_si_001.pdf [file jz5c00798_si_001.pdf]

Supporting Information for "Halogen Bond Mediated ^{13}C Overhauser Dynamic Nuclear Polarization at 9.4 Tesla"

Luming Yang[1*], *Tomas Orlando*[1#], *Marina Bennati*[1,2]

1. Research group ESR Spectroscopy, Max Planck Institute for Multidisciplinary Sciences, Am Fassberg 11, 37077, Göttingen, Germany.

2. Institute of Physical Chemistry, Department of Chemistry, Georg-August-University, Tammanstr. 6, 37077, Göttingen, Germany.

#Present address: National High Magnetic Field Laboratory, 1800 E. Paul Dirac Dr., 32310-3706, Tallahassee, FL, USA;

Table of Content:

Experimental methods: 3

DNP sample concentrations, parameters, and leakage factors: 4

^{13}C Overhauser DNP spectra of iodobenzene under different analyte concentration 5

^{13}C NMR spectra of analytes under OE-DNP and thermal equilibrium 6

^{13}C Overhauser DNP enhancements of analytes in cyclopentane 7

Electrostatic potential isosurfaces for halogenated analytes 8

Optimized geometries for polarizing agent-analyte complexes stabilized by the O_N...I interaction 9

Bond angles of analyte-polarizing agent complexes for iodobenzene derivatives 16

Calculated properties of polarizing agent-analyte complexes stabilized by the O_N...I interaction 17

Averaged values of polarizing agent-analyte complexes stabilized by the O_N...I interaction 22

Molecular orbital and spin density isosurfaces of analyte-polarizing agent complexes 23

Correlation between OE-DNP performance and halogen bond strength calculated using CCSD 24

Calculated and measured polarizing agent isotropic hyperfine constants 25

Correlation between $\xi(^{13}C_I)$ and $A_{iso}(^{13}C_I)$ calculated with different methods 29

Hyperfine constants of selected complexes formed by TN with twisted conformation 30

Electron spin density distribution for polarizing agent-analyte complexes of iodobenzene derivatives stabilized by the O_N...I interaction 31

Verification using other computational methods 32

Dihedral angles of complexes of TN and iodobenzene derivatives stabilized by the O_N...I interaction 34

Correlation between XB geometries and $A_{iso}(^{13}C_I)$ of complexes stabilized by the O_N...I interaction........35
Hyperfine constants and spin densities of the iodinated carbons calculated for polarizing agent-analyte complexes stabilized by competing interactions........36
Bonding geometries for the polarizing agent-analyte O_C...I, O_N...H, and PA-π interactions........37
Optimized geometries for polarizing agent-analyte complexes stabilized by the O_C...I, O_N...H, PA-π interactions........38
Orbital interaction patterns for the polarizing agent-analyte PA-π interaction........41
Electronic properties of polarizing agent-analyte complexes stabilized by various interactions........42
Chemical shifts of DNP and Boltzmann spectra for the iodobenzene derivatives, and relationship with hyperfine constants........43
Bonding geometries for the brominated and chlorinated compounds........45
Calculated properties of complexes of brominated and chlorinated analytes with TN........49
Relationship between DNP performance and hyperfine constants for all halogenated compounds investigated in this study........52
Correlation between halogen bond properties and DNP performance measured in cyclopentane........53
Electrostatic potential isosurface maxima of halogenated molecules........54
Alternative binding modes of CX_4 with TN........54
Relative surface area of the σ-hole of the halogenated analytes........55
Chemical shift differences between the DNP and Boltzmann spectra for the iodinated, brominated, and the chlorinated analytes........56
Vibrational modes of halogen bond stabilized analyte-polarizing agent complexes........57
Vibrational frequencies and OE-DNP spectral density function........58
Benchmarking vibrational frequency calculation against experimental measurements........59
Polarizing agent-analyte complex vibration induced isotropic hyperfine constant variations........60
References........60

Experimental methods:

Materials. 4-iodoanisole (98%), 4-iodotoluene (99%), iodobenzene-$^{13}C_6$ (99% ^{13}C, 99%) 1-bromo-4-iodobenzene (98%), 1-fluoro-4-iodobenzene (99%), 1-chloro-4-iodobenzene (99%), iodopentafluorobenzene (99%), chloropentafluorobenzene (99%), bromopentafluorobenzene (99%), carbon tetrabromide-^{13}C (99% ^{13}C, 99%), 4-Oxo-,2,2,6,6-TEMPO-^{15}N-d_{16} (98% D, 98% ^{15}N, 99%), and carbon tetrachloride (suitable for HPLC, ≥99.9%) were purchased from Sigma-Aldrich. 1-fluoro-2-iodobenzene (>99%) was purchased from TCI. 2,4-difluoroiodobenzene (99%) was purchased from Thermo Scientific. 2,3,4-trifluoroiodobenzene (NMR pure) was purchased from abcr.

OE-DNP procedure. OE-DNP procedure follows our published procedure in Ref. 15 in the main text. Specifically, experiments were performed using a liquid-state OE-DNP setup consisting of a 263 GHz high-power frequency-tunable gyrotron and a commercial 9.4 T NMR equipped with a custom-designed DNP probe head. Samples were prepared by codissolving 0.3 or 0.6 mol/L analyte and 0.025 mol/L 4-oxo-TEMPO-^{15}N-d_{16} in carbon tetrachloride or cyclopentane. The solution is degassed with five freeze-pump-thaw cycles under a turbopump, and packed inside a N_2-filled glovebox. DNP spectra were collected under continuous microwave irradiation, 20 Hz spinning, and continuous cooling by cold N_2 gas, leading to effective sample temperature of ~ 300 K. Boltzmann spectra were collected at 300 K. NMR spectra were collected with 30 s recovery delay and 32k points, zero-filled to 64k points, and processed with exponential window function.

Computational methods. DFT analyses were performed with ORCA 5.0.3.[1] Geometry optimization of the analyte-polarizing agent complexes were performed with the unrestricted M06-2X[2] or B3LYP-D3[3] functional, aug-cc-pVTZ basis (-PP for I and Br)[4–6], SK-MCDHF-RSC auxiliary basis, RIJCOSX[7] and SMD solvent approximation[8] (CCl_4) on grid level of defgrid3. Optimized complexes were subjected to numerical frequency calculations at the same level of theory and showed no imaginary mode. Unless otherwise noted, computational results presented in the main text and the Supporting Information were obtained from geometries optimized with unrestricted M06-2X/aug-cc-pVTZ (-PP for I and Br). Hyperfine calculations were performed with B3LYP-D3/aug-cc-pVTZ-J, DLPNO-CCSD, and other methods recorded in Tables S14 and S15. Electrostatic potential analysis was performed with Multiwfn[9,10] and visualized with VWD[11].

DNP sample concentrations, parameters, and leakage factors:

Analyte	c(analyte) (mol/L)	NS*(DNP)	NS(Boltzmann)
p-OMe	0.3	128	512
p-Me	0.6	128	1536
p-H**	0.3	16	16
p-Br	0.3	128	768
p-F	0.3	128	1536
p-Cl	0.3	128	768
o-F	0.6	512	2048
F2	0.6	256	2048
F3	0.6	256	2048
F5	0.6	256	2048
F_5ClBz	0.6	256	1024
F_5BrBz	0.6	256	1024
CBr_4**	0.6	16	16

*NS represents number of scans. **spectra were collected on ^{13}C-enriched sample.
Note: polarizing agent concentration always stays at 0.025 mol/L

^{13}C Overhauser DNP spectra of iodobenzene under different analyte concentration

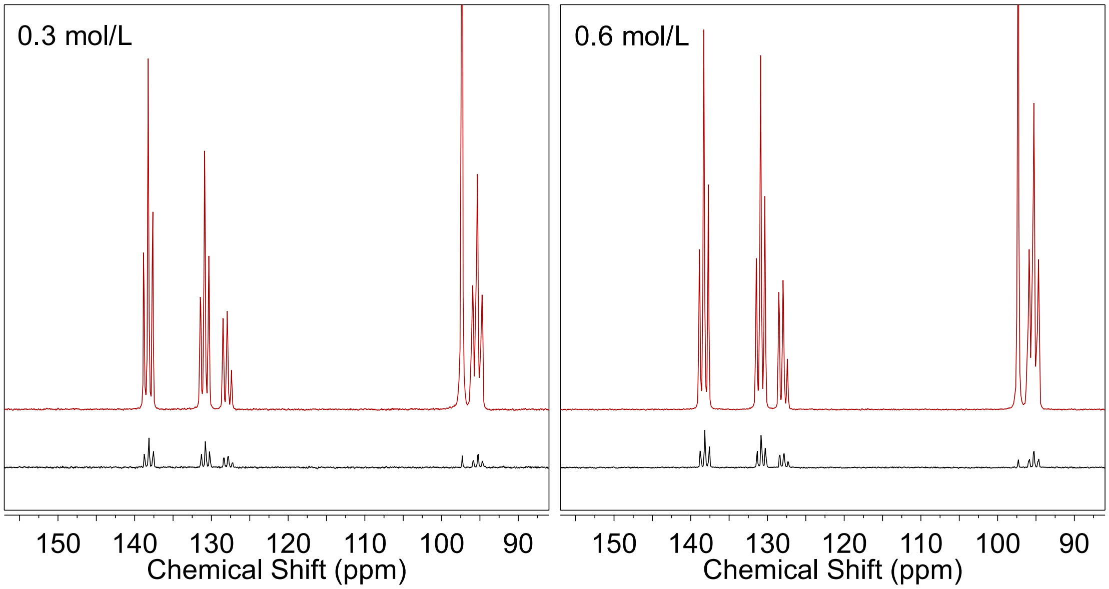

Figure S1. ^{13}C NMR spectra of 0.3 mol/L and 0.6 mol/L iodobenzene-$^{13}C_6$ (p-H) in CCl_4 containing 0.025 mol/L TN measured under OE-DNP condition (red, 16 scans) and thermal equilibrium (black, 16 scans).

Table S1. Overhauser DNP enhancements of iodobenzene-$^{13}C_6$ measured at 0.3 mol/L and 0.6 mol/L concentration and otherwise identical sample composition (with ±10% error).

Concentration	$\varepsilon(^{13}C_I)$	$\varepsilon(^{13}C2)$	$\varepsilon(^{13}C3)$	$\varepsilon(^{13}C4)$	$\varepsilon(^{13}C5)$	$\varepsilon(^{13}C6)$
0.3 mol/L	28	11	11	9	11	11
0.6 mol/L	28	11	11	9	11	11

^{13}C NMR spectra of analytes under OE-DNP and thermal equilibrium

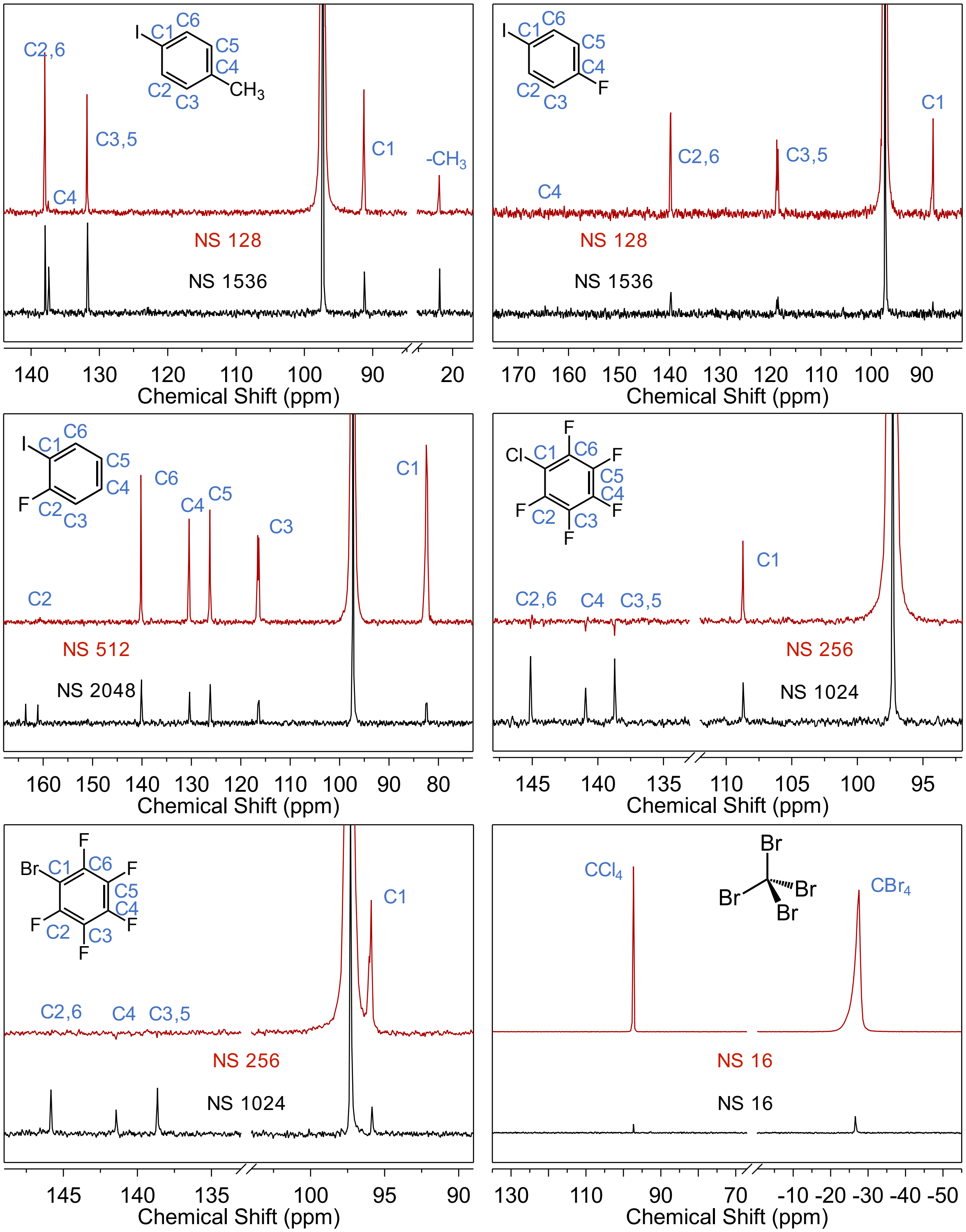

Figure S2. ^{13}C NMR spectra of iodinated, brominated, and chlorinated compounds in CCl_4 under OE-DNP (red) and thermal equilibrium (black). See Experimental methods for analyte concentrations and number of scans. Note that CBr_4 is ^{13}C-enriched.

^{13}C Overhauser DNP enhancements of analytes in cyclopentane

Table S2. Overhauser DNP enhancements of selected halogenated compounds measured in cyclopentane at 0.6 mol/L containing 0.025 mol/L TN (with ±10% error).

Analyte	ε(C–X)	ε(C2)	ε(C3)	ε(C4)	ε(C5)	ε(C6)
p-H	43	18	18	16	18	18
o-F	52	-*	20	14	14	18
F3	78	-	-	-	26	24
F5	120	-	3	-	3	-
CCl_4	132	/**	/	/	/	/
CBr_4	186	/	/	/	/	/

*|ε| < 1 under DNP conditions. **not applicable.

Electrostatic potential isosurfaces for halogenated analytes

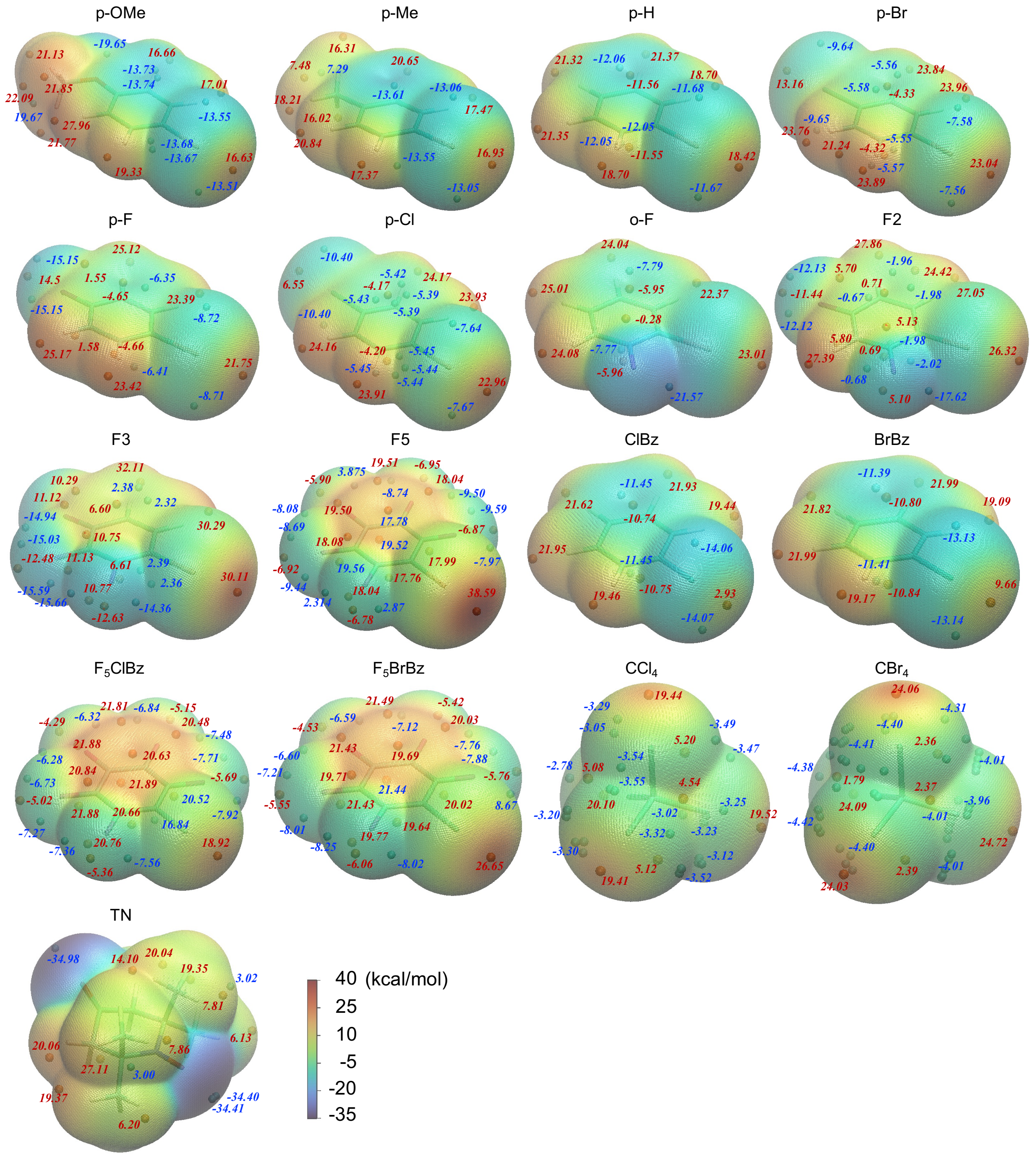

Figure S3. Electrostatic potential isosurfaces (0.001 a.u.) for the halogenated compounds and TN. Red spheres and numbers correspond to local maxima. Blue spheres and numbers correspond to local minima. ESP analyses were performed on structures optimized using M06-2X as parts of analyte-polarizing agent complexes. Structure of TN was taken from its complex with F5.

Optimized geometries for polarizing agent-analyte complexes stabilized by the O_N…I interaction

Color code: carbon, gray; hydrogen, white; oxygen, red; nitrogen, blue; iodine, purple; bromine, brown; fluorine, yellow; chlorine, green. Numbering scheme of Figures S4-S13 is shared with Tables S3-S12.

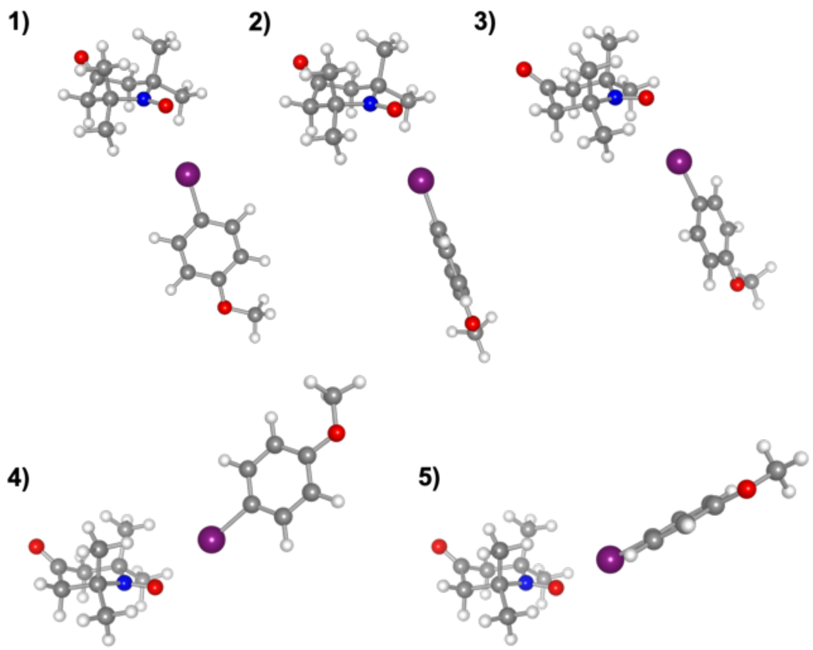

Figure S4. DFT-optimized geometries of the complexes of p-OMe and TN stabilized by the O_N…I interaction. Numbering represents individual geometries.

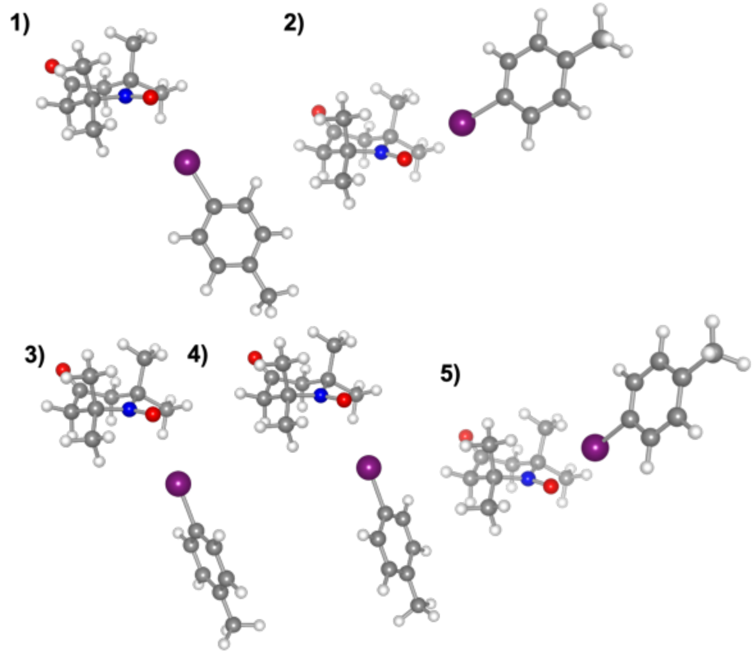

Figure S5. DFT-optimized geometries of the complexes of p-Me and TN stabilized by the O_N…I interaction. Numbering represents individual geometries.

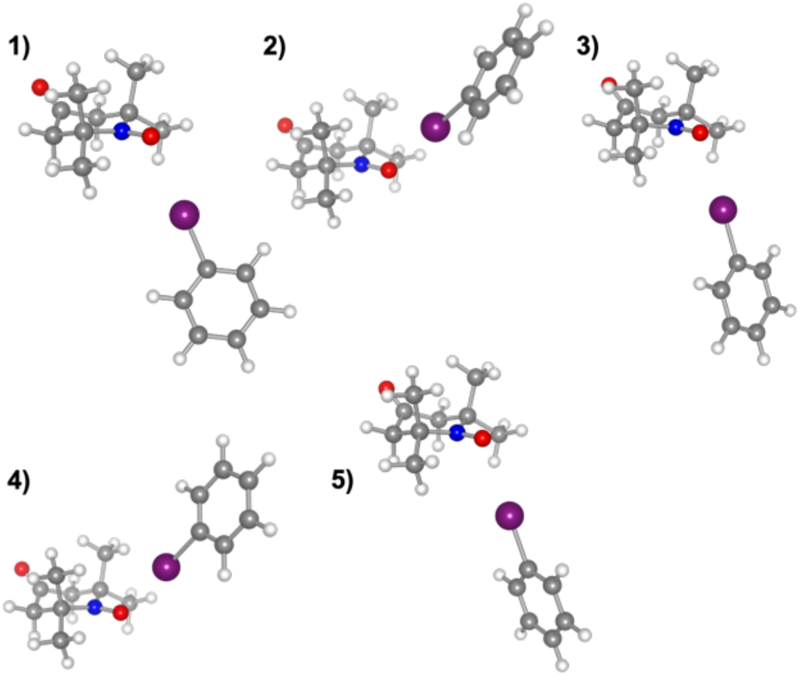

Figure S6. DFT-optimized geometries of the complexes of p-H and TN stabilized by the O_N…I interaction. Numbering represents individual geometries.

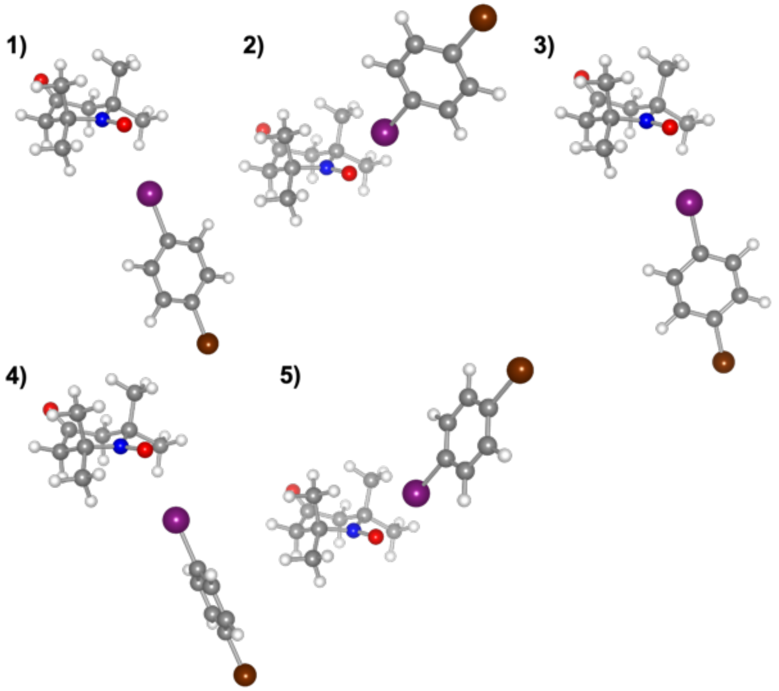

Figure S7. DFT-optimized geometries of the complexes of p-Br and TN stabilized by the O_N…I interaction. Numbering represents individual geometries.

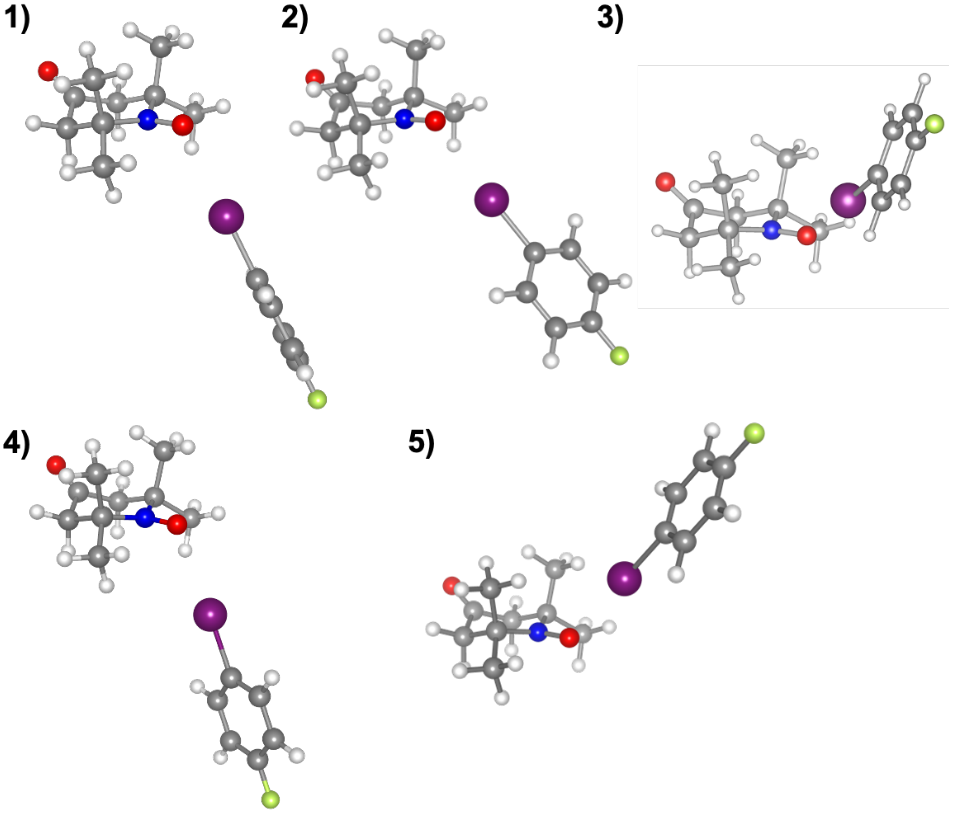

Figure S8. DFT-optimized geometries of the complexes of p-F and TN stabilized by the O_N…I interaction. Numbering represents individual geometries.

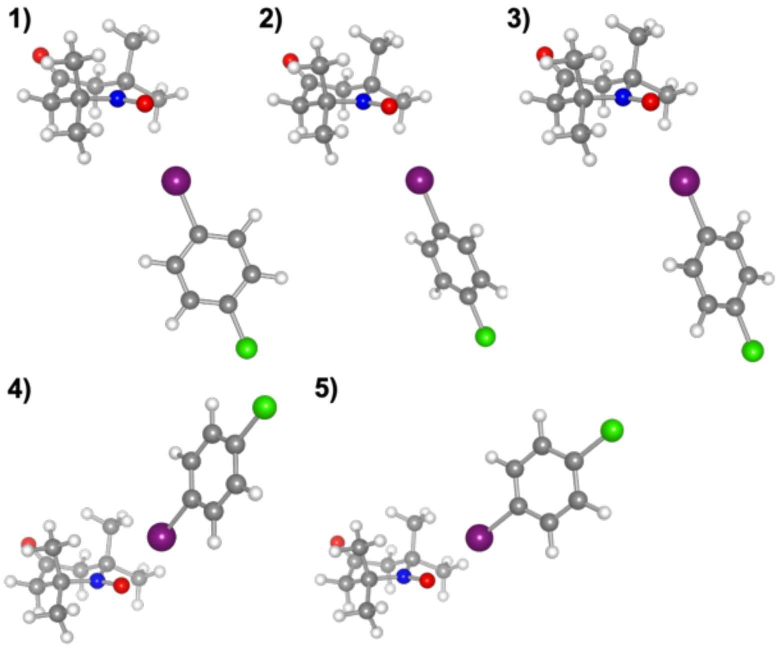

Figure S9. DFT-optimized geometries of the complexes of p-Cl and TN stabilized by the O_N…I interaction. Numbering represents individual geometries.

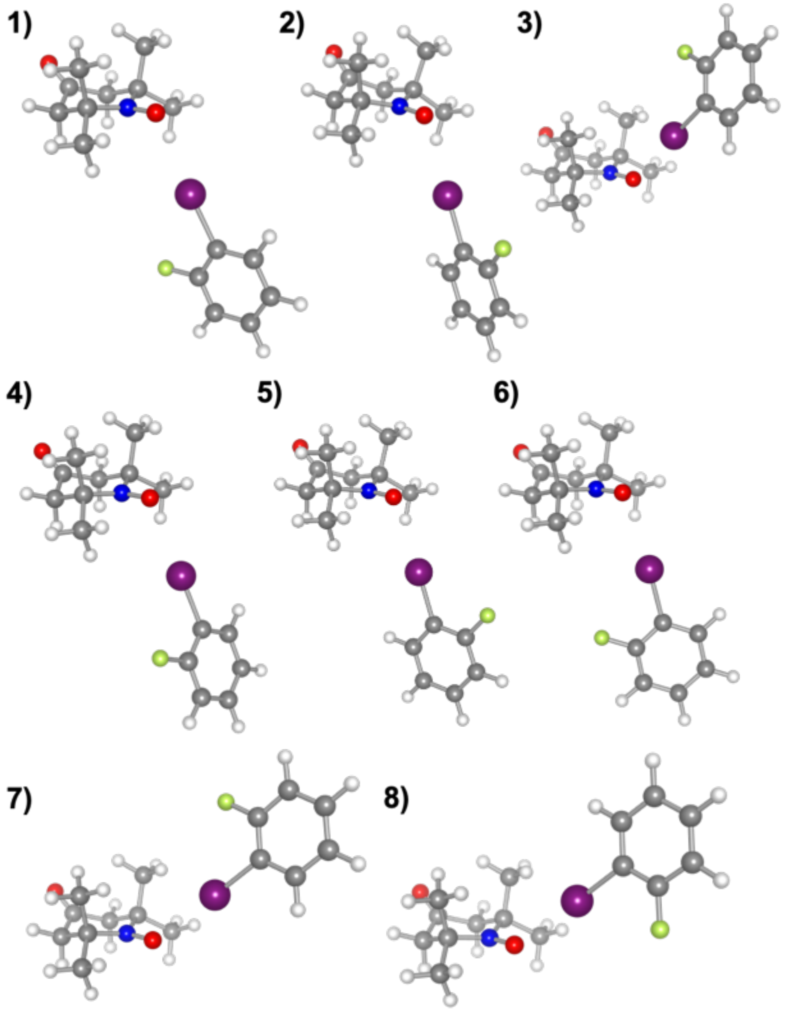

Figure S10. DFT-optimized geometries of the complexes of o-F and TN stabilized by the O_N…I interaction. Numbering represents individual geometries.

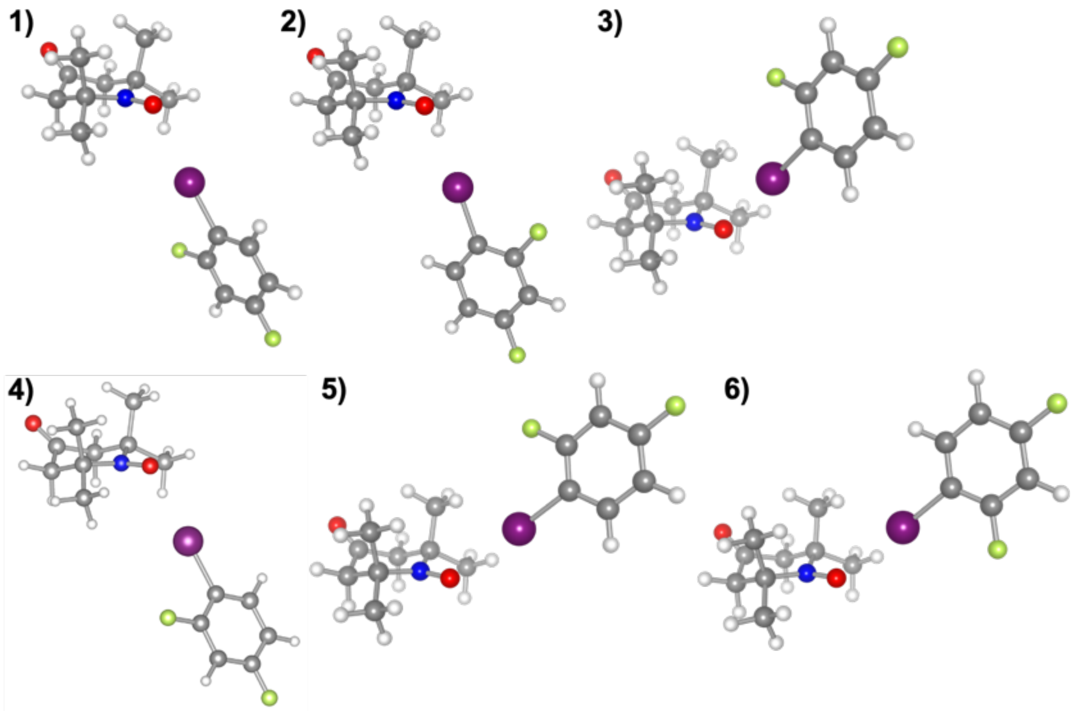

Figure S11. DFT-optimized geometries of the complexes of F2 and TN stabilized by the O_N…I interaction. Numbering represents individual geometries.

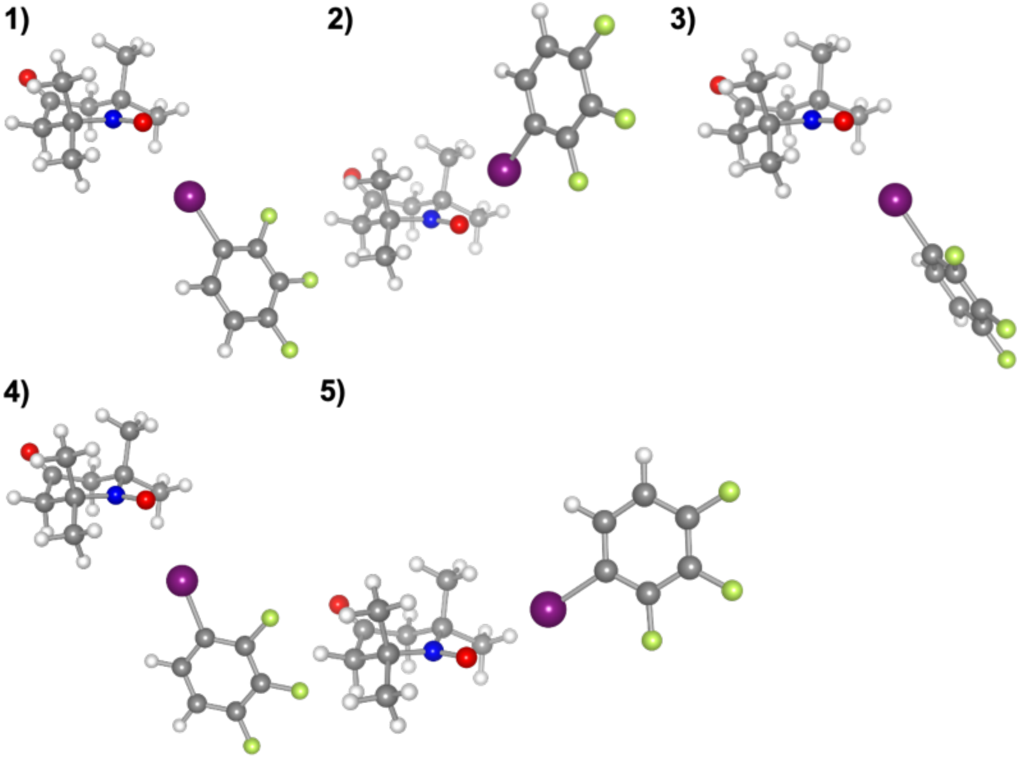

Figure S12. DFT-optimized geometries of the complexes of F3 and TN stabilized by the O_N…I interaction. Numbering represents individual geometries.

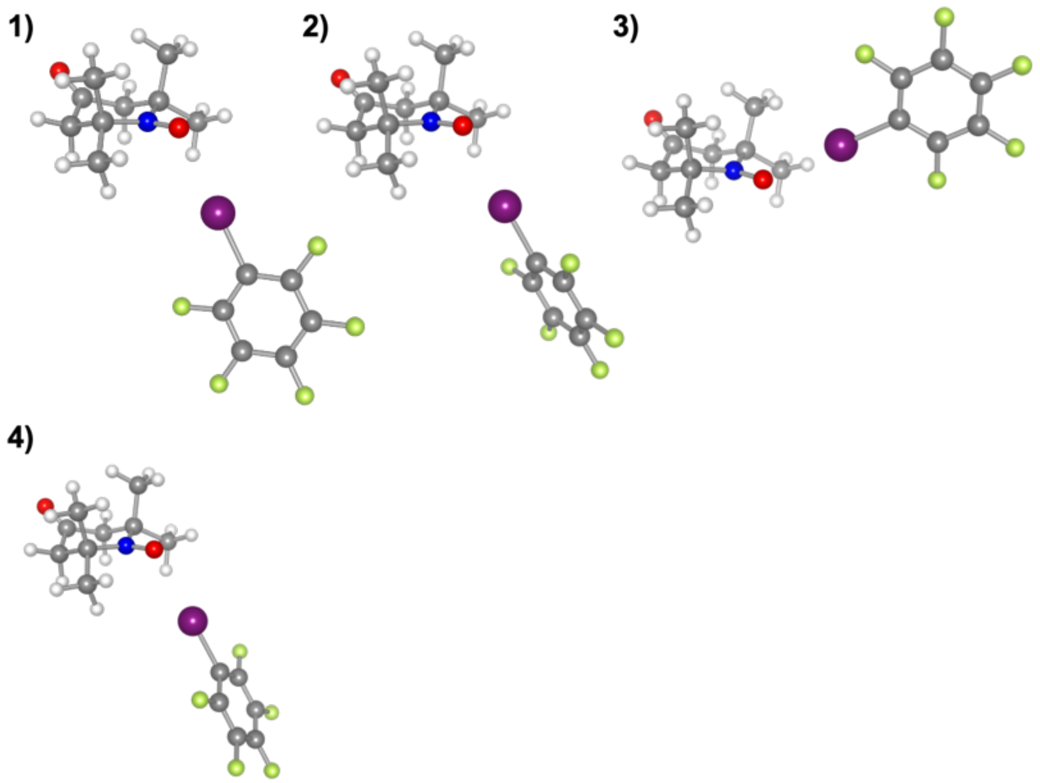

Figure S13. DFT-optimized geometries of the complexes of F5 and TN stabilized by the O_N…I interaction. Numbering represents individual geometries.

Bond angles of analyte-polarizing agent complexes for iodobenzene derivatives

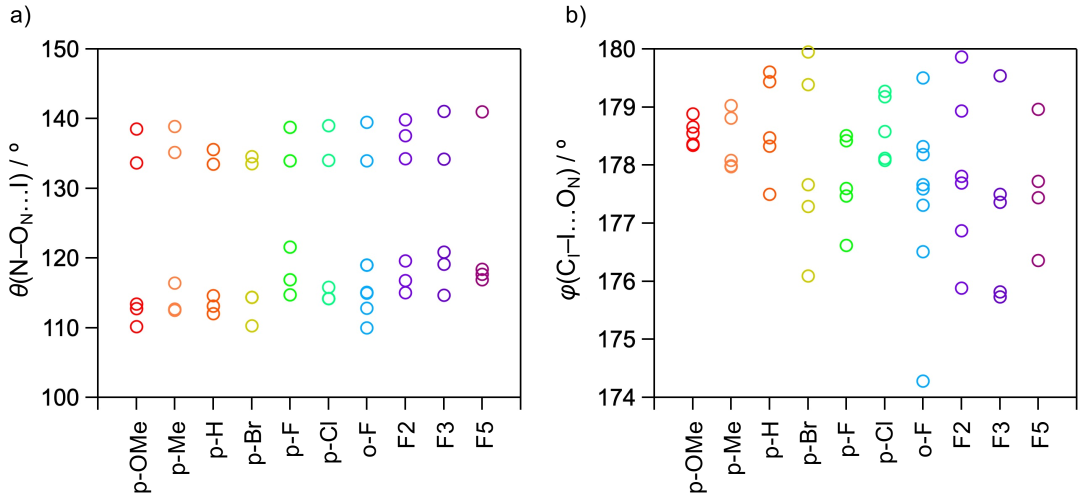

Figure S14. Bond angles $\theta(\mathrm{N–O_N}\ldots\mathrm{I})$ (a) and $\phi(\mathrm{C_I–I}\ldots\mathrm{O_N})$ from optimized geometries of polarizing agent-analyte complexes stabilized by the $\mathrm{O_N}\ldots\mathrm{I}$ interaction (b).

Calculated properties of polarizing agent-analyte complexes stabilized by the O_N…I interaction

Table S3. Calculated parameters of geometrically optimized transient complexes of TN and p-OMe. Δ*G* for the most stable geometry is set to 0. See main text for definition of the parameters. For Tables S3-S13, geometry optimization was performed with M06-2X/aug-cc-pVTZ. Hyperfine interaction and spin population were evaluated by B3LYP-D3/aug-cc-pVTZ-J.

p-OMe geometries	1	2	3	4	5
ΔG / kcal·mol^{-1}	0	+0.07	+0.14	+0.17	+0.62
θ(N–O_N…I) / °	110.18	112.74	113.43	133.64	138.52
ϕ(C_I–I…O_N) / °	178.36	178.66	178.55	178.88	178.34
d(I…O_N) / Å	3.068	3.038	3.032	3.023	3.015
ζ(dihedral) / °	268.70	270.34	270.21	87.39	115.17
E(XB) / kcal·mol^{-1}	–3.30	–3.28	–3.30	–3.11	–3.04
A_{iso}($^{13}C_I$) / MHz	4.18	4.80	5.04	7.36	5.18
ϱ($^{13}C_I$) %	0.44	0.50	0.51	0.76	0.55
s component (%)	13.8	13.9	14.1	13.9	13.4
ESP / kcal·mol^{-1}	16.63	16.63	16.66	16.58	16.63

Table S4. Calculated parameters of geometrically optimized transient complexes of TN and p-Me. Δ*G* for the most stable geometry is set to 0.

p-Me geometries	1	2	3	4	5
ΔG / kcal·mol^{-1}	0	+0.20	+0.30	+0.46	+1.07
θ(N–O_N…I) / °	116.39	138.90	112.69	112.53	134.86
ϕ(C_I–I…O_N) / °	177.97	177.77	179.03	178.81	178.53
d(I…O_N) / Å	3.011	3.017	3.034	3.035	3.012
ζ(dihedral) / °	269.91	66.50	270.61	270.01	90.29
E(XB) / kcal·mol^{-1}	–3.27	–3.01	–3.28	–3.29	–3.11
A_{iso}($^{13}C_I$) / MHz	5.40	5.18	4.74	4.78	7.30
ϱ($^{13}C_I$) %	0.56	0.56	0.50	0.51	0.77
s component (%)	13.8	13.3	13.6	13.7	13.7
ESP / kcal·mol^{-1}	16.93	16.92	16.87	16.92	16.87

Table S5. Calculated parameters of geometrically optimized transient complexes of TN and p-H. ΔG for the most stable geometry is set to 0.

p-H geometries	1	2	3	4	5
ΔG / kcal·mol^{-1}	0	+0.21	+0.34	+0.37	+0.40
θ(N–O$_N$…I) / °	114.62	135.56	112.04	133.48	113.12
ϕ(C$_I$–I…O$_N$) / °	179.60	177.50	178.33	178.47	179.44
d(I…O$_N$) / Å	3.022	3.010	3.034	3.014	3.026
ζ(dihedral) / °	268.12	94.20	269.81	86.84	270.88
E(XB) / kcal·mol^{-1}	–3.36	–3.16	–3.37	–3.18	–3.36
$A_{iso}(^{13}C_I)$ / MHz	5.16	7.19	4.80	7.43	4.98
$\varrho(^{13}C_I)$ %	0.54	0.76	0.51	0.79	0.52
s component (%)	13.8	13.7	13.7	13.7	13.8
ESP / kcal·mol^{-1}	18.42	18.38	18.36	18.37	18.37

Table S6. Calculated parameters of geometrically optimized transient complexes of TN and p-Br. ΔG for the most stable geometry is set to 0.

p-Br geometries	1	2	3	4	5
ΔG / kcal·mol^{-1}	0	+0.11	+0.25	+0.33	+0.44
θ(N–O$_N$…I) / °	114.38	134.53	110.27	114.35	133.56
ϕ(C$_I$–I…O$_N$) / °	177.29	179.39	176.09	179.95	177.66
d(I…O$_N$) / Å	3.002	2.994	3.038	3.004	2.994
ζ(dihedral) / °	270.55	93.82	269.67	270.68	87.38
E(XB) / kcal·mol^{-1}	–3.57	–3.40	–3.55	–3.58	–3.39
$A_{iso}(^{13}C_I)$ / MHz	5.48	8.25	4.91	5.53	8.32
$\varrho(^{13}C_I)$ %	0.55	0.83	0.49	0.56	0.84
s component (%)	14.3	14.3	14.4	14.2	14.2
ESP / kcal·mol^{-1}	23.04	23.00	23.00	23.02	22.99

Table S7. Calculated parameters of geometrically optimized transient complexes of TN and p-F. ΔG for the most stable geometry is set to 0.

p-F geometries	1	2	3	4	5
ΔG / kcal·mol^{-1}	0	+0.05	+0.22	+0.24	+0.94
θ(N–O$_N$…I) / °	116.89	121.56	138.78	114.71	133.94
ϕ(C$_I$–I…O$_N$) / °	178.51	176.62	178.42	177.47	177.60
d(I…O$_N$) / Å	3.009	2.983	3.007	3.009	3.016
ζ(dihedral) / °	271.83	267.38	122.95	270.35	88.53
E(XB) / kcal·mol^{-1}	–3.50	–3.43	–3.28	–3.51	–3.30
A_{iso}(^{13}C$_I$) / MHz	5.79	6.28	4.52	5.67	7.72
ϱ(^{13}C$_I$) %	0.57	0.62	0.48	0.56	0.77
s component (%)	14.4	14.5	13.4	14.4	14.3
ESP / kcal·mol^{-1}	21.75	21.76	21.78	21.74	21.72

Table S8. Calculated parameters of geometrically optimized transient complexes of TN and p-Cl. ΔG for the most stable geometry is set to 0.

p-Cl geometries	1	2	3	4	5
ΔG / kcal·mol^{-1}	0	+0.10	+0.27	+0.58	+0.65
θ(N–O$_N$…I) / °	115.78	114.21	114.20	134.03	139.01
ϕ(C$_I$–I…O$_N$) / °	179.18	179.27	178.08	178.58	178.12
d(I…O$_N$) / Å	2.998	3.005	3.001	2.992	2.988
ζ(dihedral) / °	268.94	271.00	270.43	86.84	66.77
E(XB) / kcal·mol^{-1}	–3.59	–3.57	–3.58	–3.40	–3.33
A_{iso}(^{13}C$_I$) / MHz	5.92	5.71	5.83	8.19	5.88
ϱ(^{13}C$_I$) %	0.59	0.57	0.57	0.82	0.60
s component (%)	14.5	14.5	14.5	14.2	13.8
ESP / kcal·mol^{-1}	22.96	22.94	22.89	22.91	22.88

Table S9. Calculated parameters of geometrically optimized transient complexes of TN and o-F. ΔG for the most stable geometry is set to 0.

o-F geometries	1	2	3	4	5	6	7	8
ΔG / kcal·mol^{-1}	0	+0.22	+0.41	+0.53	+0.58	+0.73	+0.77	+0.96
θ(N–O$_N$…I) / °	115.08	118.98	133.96	112.81	114.99	109.98	137.45	139.48
ϕ(C$_I$–I…O$_N$) / °	177.31	178.32	179.50	178.18	176.51	174.28	177.59	177.66
d(I…O$_N$) / Å	2.997	2.968	2.978	2.984	2.994	3.004	2.965	2.969
ζ(dihedral) / °	271.05	270.16	87.51	270.42	269.87	268.69	66.26	65.36
E(XB) / kcal·mol^{-1}	–3.98	–3.70	–3.73	–3.84	–3.71	–3.92	–3.65	–3.47
A_{iso}(^{13}C$_I$) / MHz	5.86	6.45	8.29	5.84	5.86	5.44	6.00	5.87
ϱ(^{13}C$_I$) %	0.56	0.62	0.82	0.57	0.57	0.57	0.62	0.59
s component (%)	14.6	14.7	14.4	14.6	14.6	14.7	13.7	13.8
ESP / kcal·mol^{-1}	23.01	23.01	22.99	22.99	23.00	22.89	22.90	22.93

Table S10. Calculated parameters of geometrically optimized transient complexes of TN and F2. ΔG for the most stable geometry is set to 0.

F2 geometries	1	2	3	4	5	6
ΔG / kcal·mol^{-1}	0	+0.25	+0.29	+0.43	+0.59	+0.83
θ(N–O$_N$…I) / °	119.59	115.04	134.23	116.76	137.55	139.87
ϕ(C$_I$–I…O$_N$) / °	176.87	175.88	179.86	178.93	177.81	177.69
d(I…O$_N$) / Å	2.958	2.989	2.953	2.954	2.962	2.967
ζ(dihedral) / °	273.74	269.86	89.6	269.18	65.15	63.93
E(XB) / kcal·mol^{-1}	–4.09	–3.86	–3.89	–4.03	–3.81	–3.64
A_{iso}(^{13}C$_I$) / MHz	7.00	6.32	9.17	7.13	6.25	5.95
ϱ(^{13}C$_I$) %	0.64	0.58	0.86	0.65	0.62	0.58
s component (%)	15.3	15.2	14.9	15.2	14.2	14.3
ESP / kcal·mol^{-1}	26.32	26.30	26.20	26.24	26.21	26.27

Table S11. Calculated parameters of geometrically optimized transient complexes of TN and F3. ΔG for the most stable geometry is set to 0.

F3 geometries	1	2	3	4	5
ΔG / kcal·mol^{-1}	0	+0.19	+0.30	+0.44	+0.77
θ(N–O$_N$…I) / °	120.83	134.18	119.10	114.67	141.05
ϕ(C$_I$–I…O$_N$) / °	179.54	175.73	177.50	177.36	175.82
d(I…O$_N$) / Å	2.920	2.969	2.950	2.943	2.958
ζ(dihedral) / °	266.97	90.07	268.2	269.65	54.71
E(XB) / kcal·mol^{-1}	–4.19	–3.87	–4.10	–4.10	–3.88
A_{iso}(^{13}C$_I$) / MHz	8.05	9.32	7.40	7.27	4.72
ϱ(^{13}C$_I$) %	0.71	0.85	0.66	0.65	0.47
s component (%)	15.5	15.0	15.4	13.4	13.7
ESP / kcal·mol^{-1}	30.11	30.20	30.18	30.15	30.23

Table S12. Calculated parameters of geometrically optimized transient complexes of TN and F5. ΔG for the most stable geometry is set to 0.

F5 geometries	1	2	3	4
ΔG / kcal·mol^{-1}	0	+0.52	+0.75	+0.80
θ(N–O$_N$…I) / °	118.41	116.91	141.01	117.67
ϕ(C$_I$–I…O$_N$) / °	178.96	177.72	176.36	177.44
d(I…O$_N$) / Å	2.863	2.870	2.874	2.876
ζ(dihedral) / °	269.75	271.28	52.15	269.48
E(XB) / kcal·mol^{-1}	–5.14	–5.13	–4.80	–5.12
A_{iso}(^{13}C$_I$) / MHz	9.89	9.68	5.58	9.70
ϱ(^{13}C$_I$) %	0.82	0.80	0.52	0.80
s component (%)	16.0	16.0	14.1	16.0
ESP / kcal·mol^{-1}	38.59	38.62	38.57	38.61

Averaged values of polarizing agent-analyte complexes stabilized by the O_N…I interaction

Table S13. Averaged values of calculated parameters of geometrically optimized transient complexes of TN and ten iodobenzene derivatives. See Table S15 for $A_{iso}(^{13}C_I)$.

Analyte	$d(I…O_N)$ / Å	E(XB) / kcal·mol^{-1}	$V_{s,max}$(CI) / kcal·mol^{-1}	$\varrho(^{13}C_I)$ / %	s-orbital component / %	$\varrho(^{15}NO)$ / %
p-OMe	3.039	–3.23	16.63	0.54	13.86	87.66
p-Me	3.021	–3.21	16.93	0.55	13.63	87.62
p-H	3.021	–3.29	18.42	0.62	13.75	87.57
p-Br	3.006	–3.51	23.04	0.64	14.30	87.47
p-F	3.001	–3.43	21.75	0.57	14.25	87.55
p-Cl	2.999	–3.53	22.96	0.61	14.40	87.45
o-F	2.984	–3.80	23.01	0.61	14.48	87.42
F2	2.963	–3.94	26.32	0.66	14.99	87.38
F3	2.944	–4.06	30.11	0.70	15.20	87.24
F5	2.868	–5.07	38.59	0.77	15.69	86.76

Molecular orbital and spin density isosurfaces of analyte-polarizing agent complexes

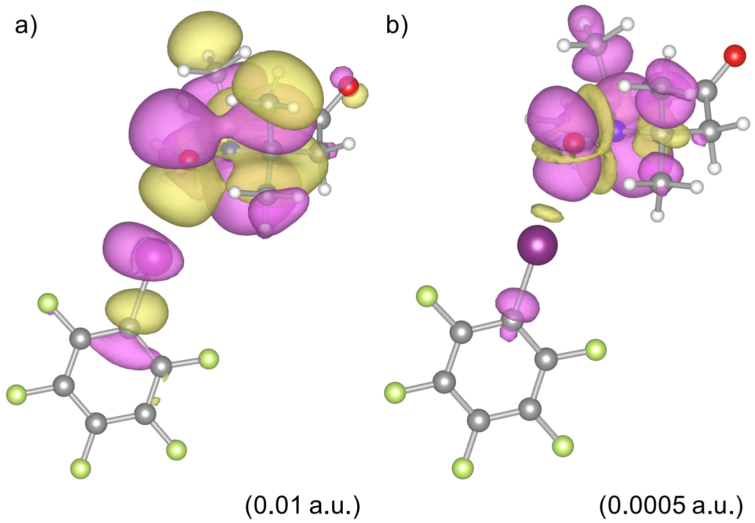

Figure S15. (a) Singly-occupied molecular orbital (SOMO) and (b) spin density isosurfaces for geometry 2 (see Table S12) of F5-TN complex. Pink and yellow correspond to positive and negative densities.

Correlation between OE-DNP performance and halogen bond strength calculated using CCSD

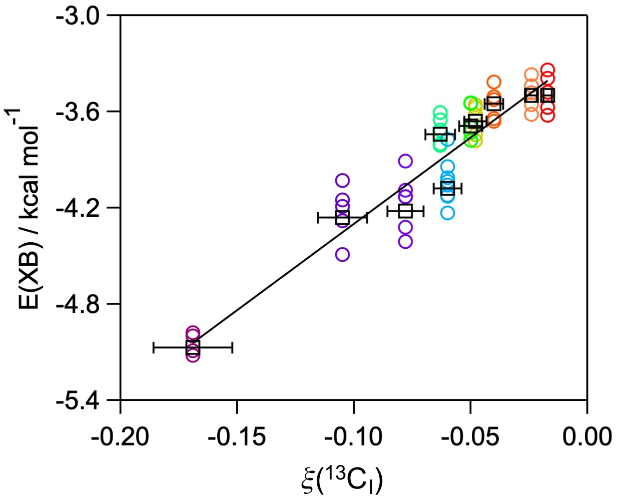

Figure S16. Correlation between $\xi(^{13}C_I)$ and E(XB) calculated using DLPNO-CCSD/aug-cc-pVTZ-J on geometries optimized with M06-2X/auc-cc-pVTZ. Colored circles represent values of individual geometries, and black squares represent averaged values according to equation 2 in the main text. Black solid line is guide-to-eye for the averaged data ($r^2 = 0.94$).

Calculated and measured polarizing agent isotropic hyperfine constants

Table S14. Calculated and measured isotropic hyperfine constants, $A_{iso}(^{15}N)$, of the ^{15}N-enriched nitroxide polarizing agents in DNP samples of different iodobenzene derivatives.

Analyte	Method 1	Method 2	Method 3	Method 4	Method 5	Method 6	Expt.
p-OMe	51.5	52.2	52.8	56.2	57.2	56.1	54.2
p-Me	51.4	52.1	52.7	56.2	57.1	56.0	52.0
p-H	51.5	52.2	52.8	56.2	57.2	56.1	54.2
p-Br	51.6	52.3	52.9	56.2	57.2	56.3	50.9
p-F	51.6	52.2	52.8	56.4	57.3	56.2	50.9
p-Cl	51.7	52.3	53.0	56.4	57.3	56.3	53.1
o-F	51.5	52.2	52.8	56.3	57.2	56.3	54.7
F2	51.6	52.4	52.9	56.5	57.4	56.5	53.1
F3	51.5	52.3	52.8	56.5	57.4	56.4	53.6
F5	52.0	52.8	53.2	57.0	57.7	57.3	53.6
RMSE	1.93	1.46	1.28	3.59	4.45	3.55	-

Method 1. PBE0-D3, x2c-TZVPall-s
Method 2. PBE0-D3, EPR-III
Method 3. B3LYP-D3, aug-cc-pVTZ-J
Method 4. M06-2X, aug-cc-pVTZ-J
Method 5. M06-2X, ZORA, ZORA-TZVPP
Method 6. DLPNO-CCSD

RMSE of the computed $A_{iso}(^{15}N)$ against experimental data is calculated as:

$$RMSE = \sqrt{\frac{1}{n}\sum_{j=1}^{n}\left(A_j^{calc} - A_j^{expt}\right)^2}$$

Here, methods 1-5 test the DFT approach with common hybrid functionals. x2c-TZVPall-s is Karlsruhe basis for exact two-component (X2C) calculation, optimized for NMR shielding tensor calculation, and is available for H-Rn.[12] EPR-III is basis developed by Barone for EPR calculation, but only available for H, C, N, O, F.[13] So, it's paired with IGLO-III (Kutzelnigg's basis for NMR/EPR calculation) for Cl,[14] DKH-def2-TZVP and SARC-DKH-TZVP full electron bases for Br and I.[7,15] The Sauer's basis aug-cc-pVTZ-J is uncontracted from aug-cc-pVTZ and augmented with additional s-functions.[16] It has been used for benchmarking ^{14}N hyperfine interaction for nitroxide radicals.[16] Because iodine could generate relativistic effect, we further tested the zeroth-order regular approximation approach (ZORA) for describing relativistic correction with the uncontracted Karlsruhe orbitals provided by ORCA.[16] Method 6 applies the domain-based local pair-natural orbital coupled-cluster singles and doubles theory,[17] often regarded as standard for benchmarking hyperfine calculations.

As discussed in the main text, we report results obtained from multiple computational methods to emphasize that the main conclusion of this study, i.e. the important role of halogen bond in mediating OE-DNP, is valid for various data analysis approaches. While CCSD is regarded as the

“golden computational standard”, we note that CCSD does not always give the best agreement with experiment when compared to DFT, which is parameterized towards specific systems. For example, M06-2X has been shown to provide results closer to the experimental values than DLPNO-CCSD when evaluating $A_{iso}(^{14}N)$ of several nitroxide radicals in solution.[18] For certain hydrated semiquinone radicals, B3LYP-D3 has shown comparable or slightly better performance than DLPNO-CCSD for calculating $A_{iso}(^{1}H)$ and $A_{iso}(^{17}O)$.[17] Obtaining an experimental dataset for benchmarking the intermolecular $A_{iso}(^{13}C)$ of the halogenated analytes in solution at room temperature remains a challenging task due to fast electron spin relaxation and broadening related to ensemble averaging. Nevertheless, both CCSD and typical DFT methods point towards a strong correlation between halogen bond, the induced hyperfine interaction, and the observed OE-DNP performances (see both main text and SI), which supports our study.

Table S15. Hyperfine constants calculated with different methods for optimized analyte-polarizing agent complexes stabilized by the O_N…X halogen bond and Gibbs free energy weighting. All values are in unit of MHz. All calculations here were based on geometry optimization and frequency calculations with the M06-2X functional. See Figure S20 for calculations based on geometry optimization and frequency calculations with B3LYP.

Analyte	Method 1	Method 2	Method 3	Method 4	Method 5	Method 6
p-OMe	4.85	4.63	5.23	5.63	5.85	3.57
p-Me	4.86	4.64	5.22	5.55	5.73	3.59
p-H	5.48	5.24	5.87	6.16	6.35	4.04
p-Br	6.02	5.80	6.42	6.58	6.76	4.40
p-F	5.39	5.12	5.76	5.97	6.15	3.93
p-Cl	5.71	5.45	6.11	6.28	6.47	4.16
o-F	5.89	5.60	6.27	6.39	6.60	4.10
F2	6.72	6.41	7.12	7.11	7.31	4.68
F3	7.41	7.04	7.80	7.66	7.90	5.10
F5	8.84	8.21	9.19	8.55	8.77	6.28
ClBz	1.08	1.02	1.22	1.57	1.56	0.46
BrBz	2.05	1.94	2.29	2.72	2.82	1.48
F_5ClBz	2.65	2.58	3.03	3.04	2.95	2.01
F_5BrBz	3.75	4.08	4.70	4.28	4.41	2.81
CCl_4	6.75	6.44	8.31	5.42	5.45	7.68
CBr_4	15.83	14.75	17.68	10.44	10.64	12.71

Method 1. PBE0-D3, x2c-TZVPall-s
Method 2. PBE0-D3, EPR-III
Method 3. B3LYP-D3, aug-cc-pVTZ-J
Method 4. M06-2X, aug-cc-pVTZ-J
Method 5. M06-2X, ZORA, ZORA-TZVPP
Method 6. DLPNO-CCSD

See next page for ORCA 5.0.3 keywords.

Examples of ORCA keywords for hyperfine calculations

Method 1. PBE0-D3, x2c-TZVPall-s
UKS PBE0 D3BJ x2c-TZVPall-s x2c/J RIJCOSX TIGHTSCF CPCM defgrid3

Method 2. PBE0-D3, EPR-III
UKS PBE0 D3BJ EPR-III SARC/J RIJCOSX TIGHTSCF CPCM defgrid3

Method 3. B3LYP-D3, aug-cc-pVTZ-J
UKS B3LYP D3BJ aug-cc-pVTZ-J SARC/J RIJCOSX TIGHTSCF CPCM defgrid3

Method 4. M06-2X, aug-cc-pVTZ-J
UKS M062X aug-cc-pVTZ-J SARC/J RIJCOSX TIGHTSCF CPCM defgrid3

Method 5. M06-2X, ZORA, ZORA-TZVPP
UKS ZORA M062X ZORA-TZVPP SARC/J TIGHTSCF CPCM defgrid3

Method 6. DLPNO-CCSD
UKS DLPNO-CCSD DLPNO-HFC1 def2-TZVP def2-TZVP/C def2/J SPLIT-RI-J nofrozencore TIGHTSCF CPCM defgrid3

Note:
1. SMD solvent model with CCl_4 was used for all calculations. The CPCM keyword is still specified due to ORCA syntax.
2. Because OE-DNP experiments use ^{15}N-enriched nitroxide polarizing agents, hyperfine calculation also considers ^{15}N in all cases. This is done by adding "M 15" after the N atomic coordinate, and "ist=15, PPP=-54.101, III=0.5, QQQ=0" in the %eprnmr block.
3. Molecular coordinates are omitted for clarity. A_{iso} is calculated by specifying {aiso} in the %eprnmr block.
4. In case the bases specified in the keywords are not available for certain elements, the following are used. For non-relativistic calculations, SARC-DKH-TZVP for I, DKH-def2-TZVP for Br, IGLO-III for Cl; for relativistic calculations, SARC-ZORA-TZVPP for I.
5. Choices for the coupled-cluster calculation were based on limitations of computational resources. Particularly, only singles and doubles excitations were considered. Kohn-Sham orbitals were used, as they seem to speed up the calculation.

Correlation between $\xi(^{13}C_I)$ and $A_{iso}(^{13}C_I)$ calculated with different methods

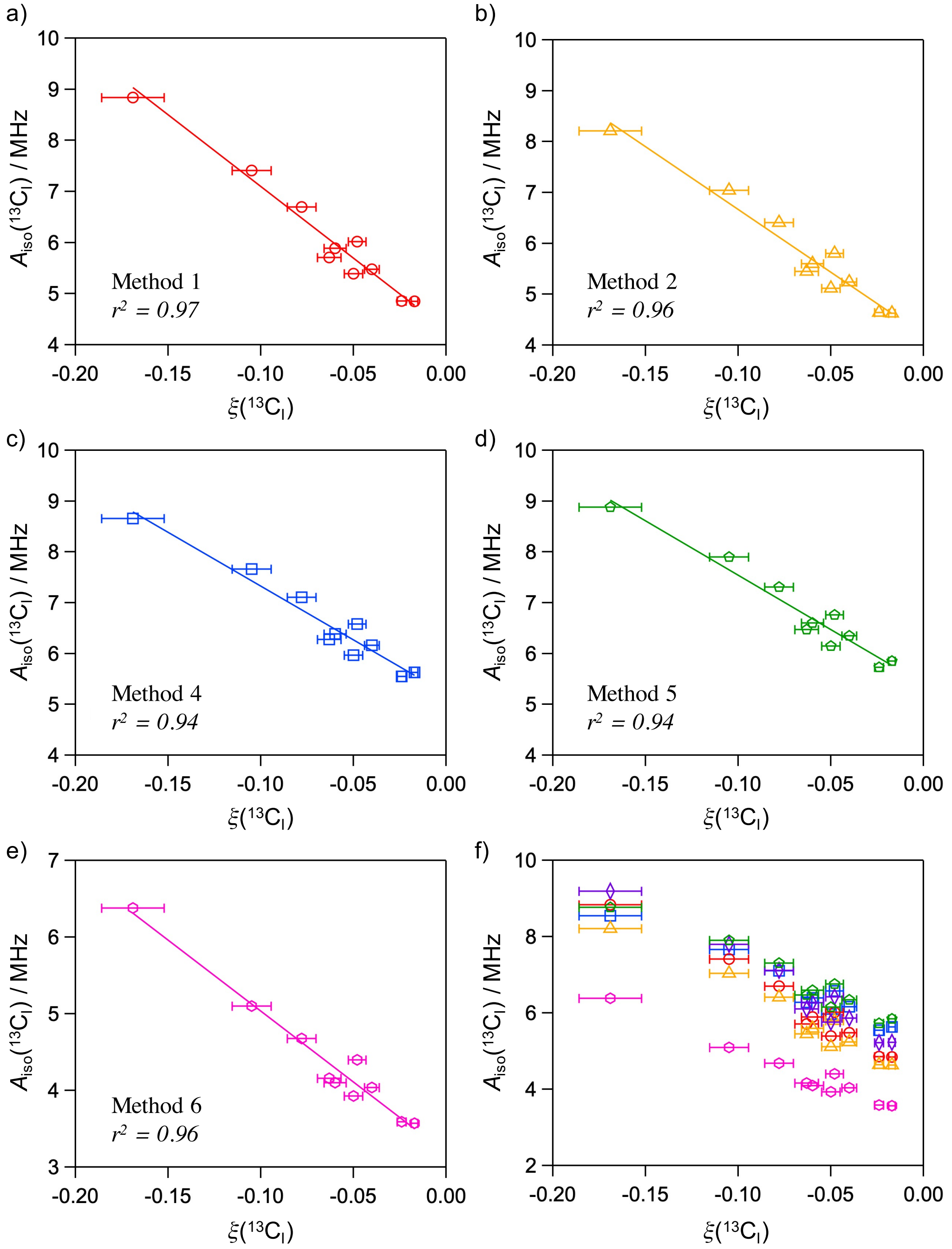

Figure S17. Comparison of correlations between $\xi(^{13}C_I)$ and $A_{iso}(^{13}C_I)$ calculated with different methods described in Table S15. (a) Method 1, red circles; (b) Method 2, yellow triangles; (c) Method 4, blue squares; (d) Method 5, green pentagons; (e) Method 6, pink hexagons; (f) superimposition of methods 1-6. Method 3 (purple triangles in (f)) is plotted separately in main text Figure 3d. All calculations here were based on geometries optimized with the M06-2X functional. See Figure S20 for calculations based on geometries optimized with B3LYP.

Hyperfine constants of selected complexes formed by TN with twisted conformation

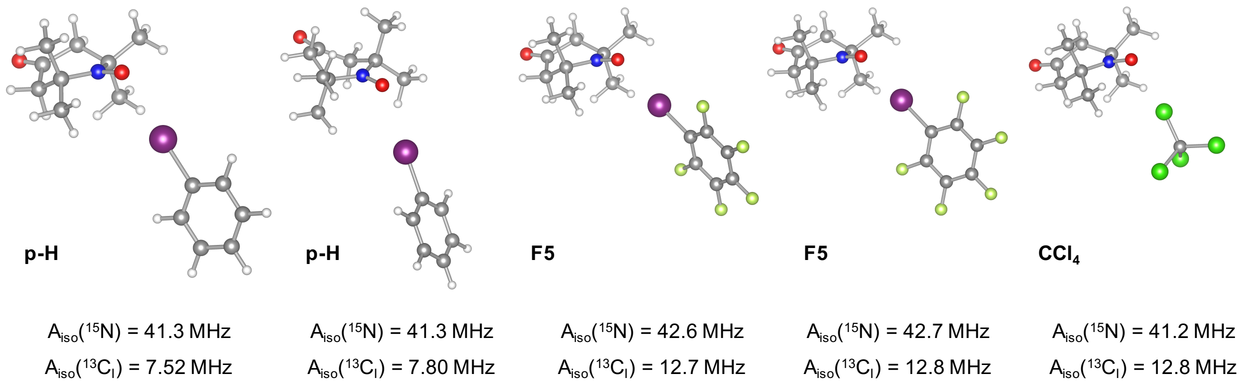

Figure S18. Optimized geometries and hyperfine constants of selected complexes of p-H, F5, and CCl_4 with TN with twisted conformations. Hyperfine constants were calculated using B3LYP-D3/aug-cc-pVTZ-J for geometries optimized with M06-2X/aug-cc-pVTZ.

Electron spin density distribution for polarizing agent-analyte complexes of iodobenzene derivatives stabilized by the O_N…I interaction

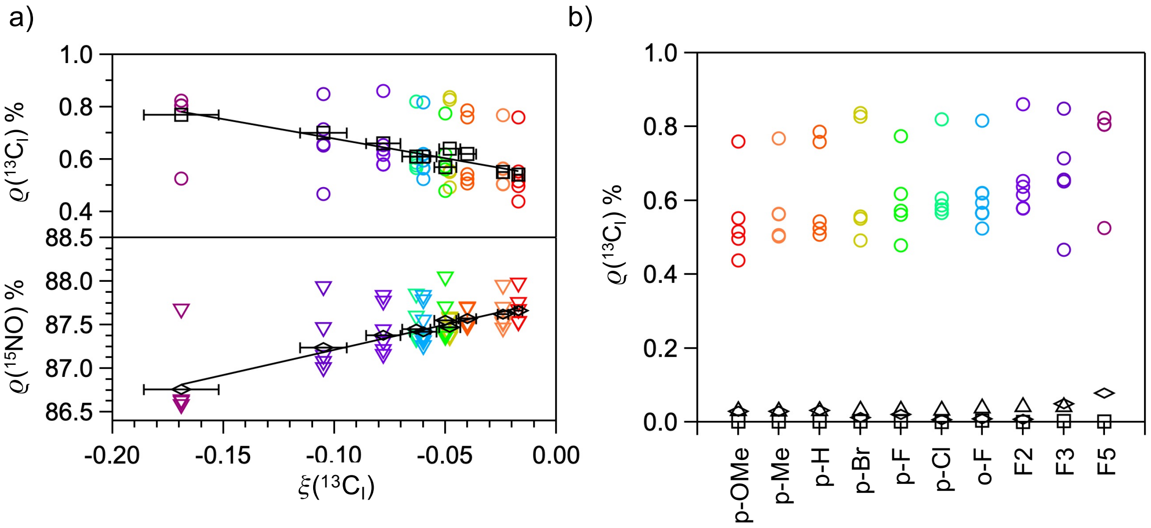

Figure S19. (a) Relationships between $\xi(^{13}C_I)$, $\varrho(^{13}C_I)$ and $\varrho(^{15}NO)$. Colored circles and triangles represent values for individual geometries. Black squares and diamonds represent averaged values. Black lines are guides-to-eye obtained from linear fitting the averaged data. (b) $\varrho(^{13}C_I)$ of polarizing agent-analyte complexes stabilized by different interactions. Colored circles represent O_N…I as in (a). Black squares, triangles, and diamonds represent O_C…I, O_N…H, and PA-π, respectively. Quality of linearity in panel (a): r^2 = 0.89 top, r^2 = 0.98 bottom. Geometry optimization was performed with M06-2X/aug-cc-pVTZ. Spin population were evaluated by B3LYP-D3/aug-cc-pVTZ-J.

Verification using other computational methods

Table S16. Averaged parameters of transient complexes of TN and ten iodobenzene derivatives optimized using B3LYP-D3. Hyperfine constants and spin densities were evaluated with EPR-III (HCNOF), IGLO-III (Cl), DKH-def2-TZVP (Br), and SARC-DKH-TZVP (I).

Analyte	$d(I...O_N)$ / Å	E(XB) / kcal·mol^{-1}	$A_{iso}(^{13}C_I)$ / MHz	$\varrho(^{13}C_I)$ / %	s-orbital component / %	$\varrho(^{15}NO)$ / %
p-OMe	3.070	–3.94	4.58	0.52	14.67	88.14
p-Me	3.055	–3.96	4.95	0.56	14.42	88.15
p-H	3.022	–3.97	5.42	0.61	14.48	87.66
p-Br	3.017	–4.29	6.15	0.66	15.00	87.90
p-F	3.019	–4.18	5.62	0.61	14.94	87.94
p-Cl	3.018	–4.28	5.95	0.64	14.98	87.91
o-F	2.988	–4.65	6.18	0.64	15.29	87.80
F2	2.969	–4.77	6.89	0.68	15.82	87.72
F3	2.935	–5.03	8.46	0.80	16.10	87.50
F5	2.862	–5.98	9.50	0.82	16.86	87.20

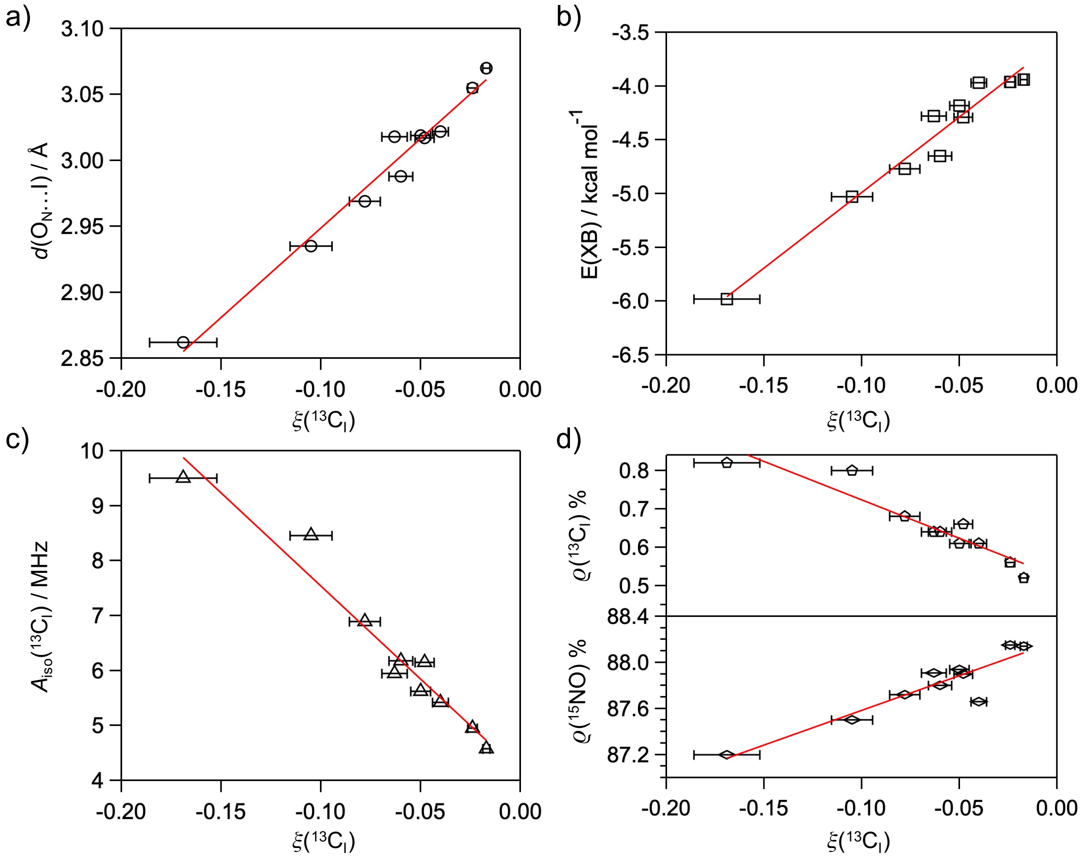

Figure S20. Correlations between $\xi(^{13}C_I)$ and the averaged $d(O_N...I)$ (a, black circles), E(XB) (b, black squares), $A_{iso}(^{13}C_I)$ (c, black triangles), and $\varrho(^{13}C_I)$ as well as $\varrho(^{15}NO)$ (d, black pentagons and diamonds, respectively). Red lines are guides-to-eye, with $r^2 = 0.97$ (a), 0.96 (b), 0.95 (c), and $r^2 = 0.88$ (d, $\varrho(^{13}C)$), $r^2 = 0.85$ (d, $\varrho(^{15}NO)$). Parameters were calculated for TN complexes with the iodobenzene derivatives stabilized by the $O_N...I$ halogen bond.

Dihedral angles of complexes of TN and iodobenzene derivatives stabilized by the O_N…I interaction

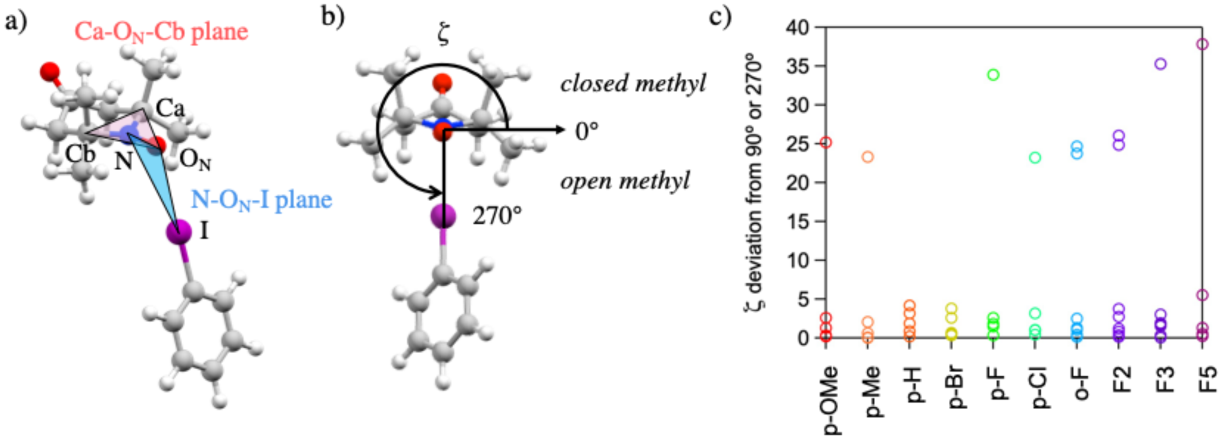

Figure S21. (a, b) Representation of the dihedral angle ζ, as defined by the Ca-O_N-Cb and N-O_N…I planes; (c) Deviation of ζ from 90° or 270° (absolute values) for individual polarizing agent-analyte geometries stabilized by the O_N…I interaction. Geometry optimization was performed with M06-2X/aug-cc-pVTZ.

Correlation between XB geometries and $A_{iso}(^{13}C_I)$ of complexes stabilized by the O_N…I interaction

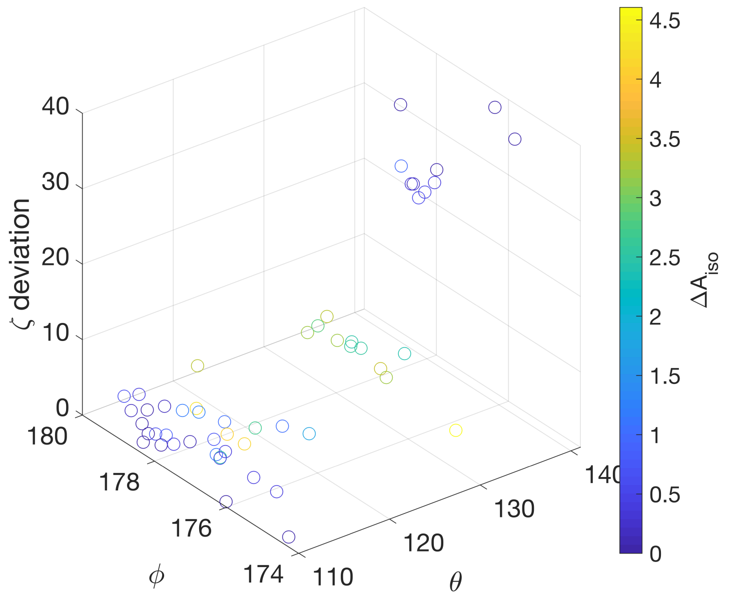

Figure S22. Correlation between θ(N–O_N…I), ϕ(C_I–I…O_N) and ζ with $\Delta A_{iso}(^{13}C_I)$ for individual geometries of polarizing agent-analyte complexes stabilized by the O_N…I halogen bond (optimized by M06-2X). Here, ζ deviation represents the smallest absolute deviation of ζ from 90° or 270° for each geometry. $\Delta A_{iso}(^{13}C_I)$ represents deviation from the lowest value of individual geometries for a certain polarizing agent-analyte combination.

Hyperfine constants and spin densities of the iodinated carbons calculated for polarizing agent-analyte complexes stabilized by competing interactions

Table S17. $A_{iso}(^{13}C_I)$ and $\varrho(^{13}C_I)$ values calculated from polarizing agent-analyte complexes stabilized by O_C…I, O_N…H, and PA-π interactions. Geometry optimization was performed with M06-2X/aug-cc-pVTZ. Hyperfine interaction and spin population were evaluated by B3LYP-D3/aug-cc-pVTZ-J.

Analyte	$A_{iso}(^{13}C_I)$ / MHz			$\varrho(^{13}C_I)$ / %		
	O_C…I	O_N…H	PA-π	O_C…I	O_N…H	PA-π
p-OMe	-0.0083	0.51	-0.16	0.001	0.030	0.029
p-Me	-0.0081	0.47	-0.16	0.001	0.029	0.029
p-H	-0.0081	0.44	-0.16	0.001	0.030	0.031
p-Br	0.012	0.47	-0.24	0.0013	0.031	0.012
p-F	-0.0089	0.47	-0.21	0.0011	0.031	0.020
p-Cl	-0.0004	0.47	-0.24	0.0000	0.032	0.006
o-F	0.026	0.43	-0.21	0.0031	0.038	0.009
F2	0.0064	0.44	-0.18	0.0005	0.040	0.007
F3	0.021	0.53	-0.09	0.0020	0.041	0.049
F5	-0.0055	-*	-0.04	0.0007	-	0.078

*not applicable

Bonding geometries for the polarizing agent-analyte O_C…I, O_N…H, and PA-π interactions

Table S18. Bonding angles of polarizing agent-analyte complexes stabilized by the O_C…I, O_N…H, and PA-π interactions. See Scheme S1 for definition of angles. See main text Scheme 2 for the labeling of O_N, O_C, and C_I.

Analyte	$\alpha(O_C\ldots I–C_I)$ / °	$\beta(C_I–I\ldots O_N)$ / °	$\lambda(N–O_N\ldots C_I)$ / °
p-OMe	175.3	67.6	92.0
p-Me	175.0	68.3	92.3
p-H	175.0	69.2	91.7
p-Br	177.7	68.8	96.3
p-F	174.7	68.9	94.6
p-Cl	179.9	68.8	96.8
o-F	177.0	68.1	95.0
F2	175.4	68.2	96.1
F3	177.2	67.1	100.0
F5	177.8	-	100.3

Scheme S1. Bonding angles of polarizing agent analyte complexes stabilized by the O_C…I, O_N…H, and PA-π interactions, as described in Table S18.

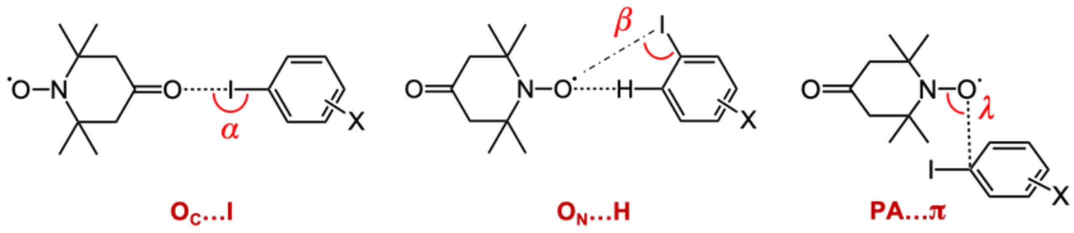

Optimized geometries for polarizing agent-analyte complexes stabilized by the O_C…I, O_N…H, PA-π interactions

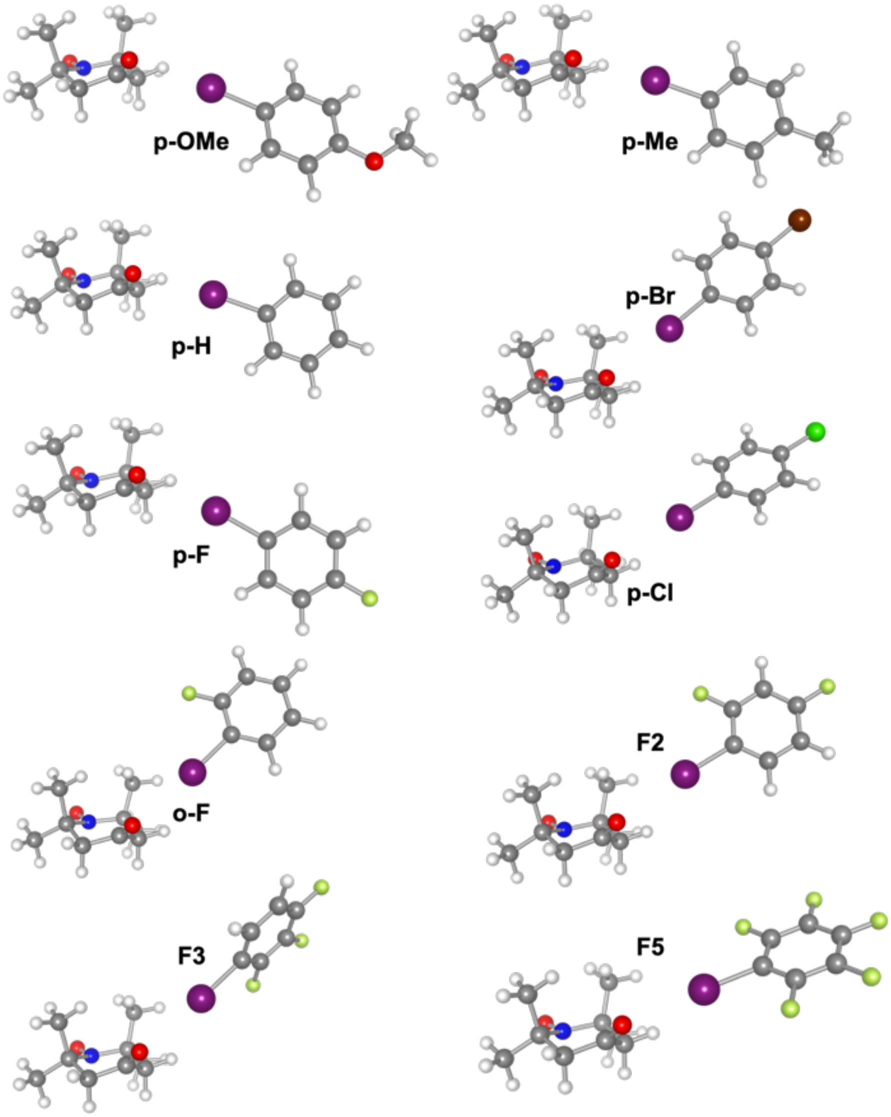

Figure S23. DFT-optimized geometries of the complexes of iodobenzene derivatives and TN stabilized by the O_C…I interaction.

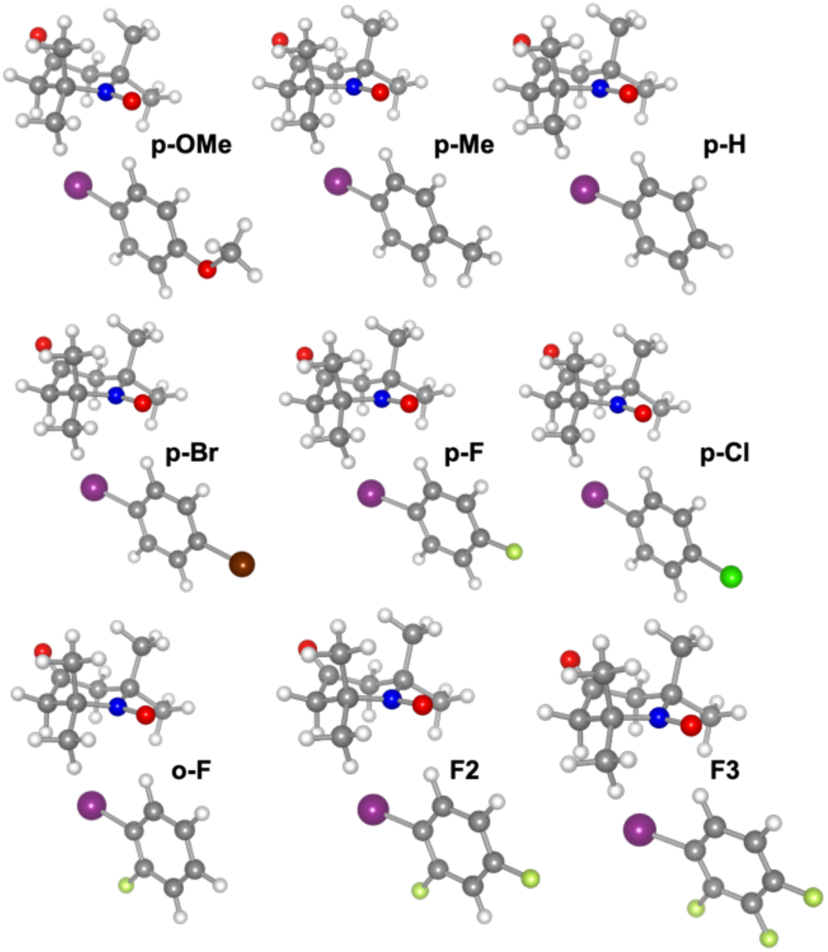

Figure S24. DFT-optimized geometries of the complexes of iodobenzene derivatives and TN stabilized by the O_N…H interaction.

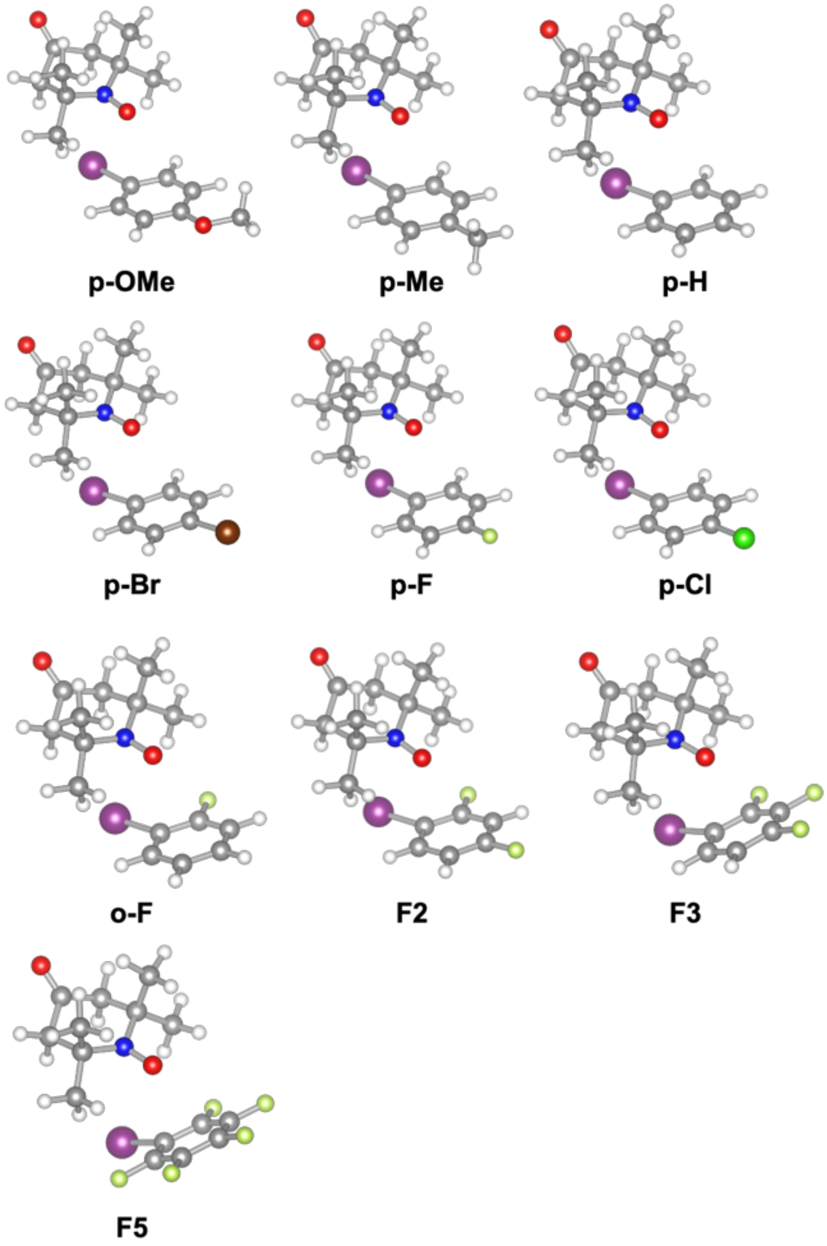

Figure S25. DFT-optimized geometries of the complexes of iodobenzene derivatives and TN stabilized by the PA-π interaction.

Orbital interaction patterns for the polarizing agent-analyte PA-π interaction

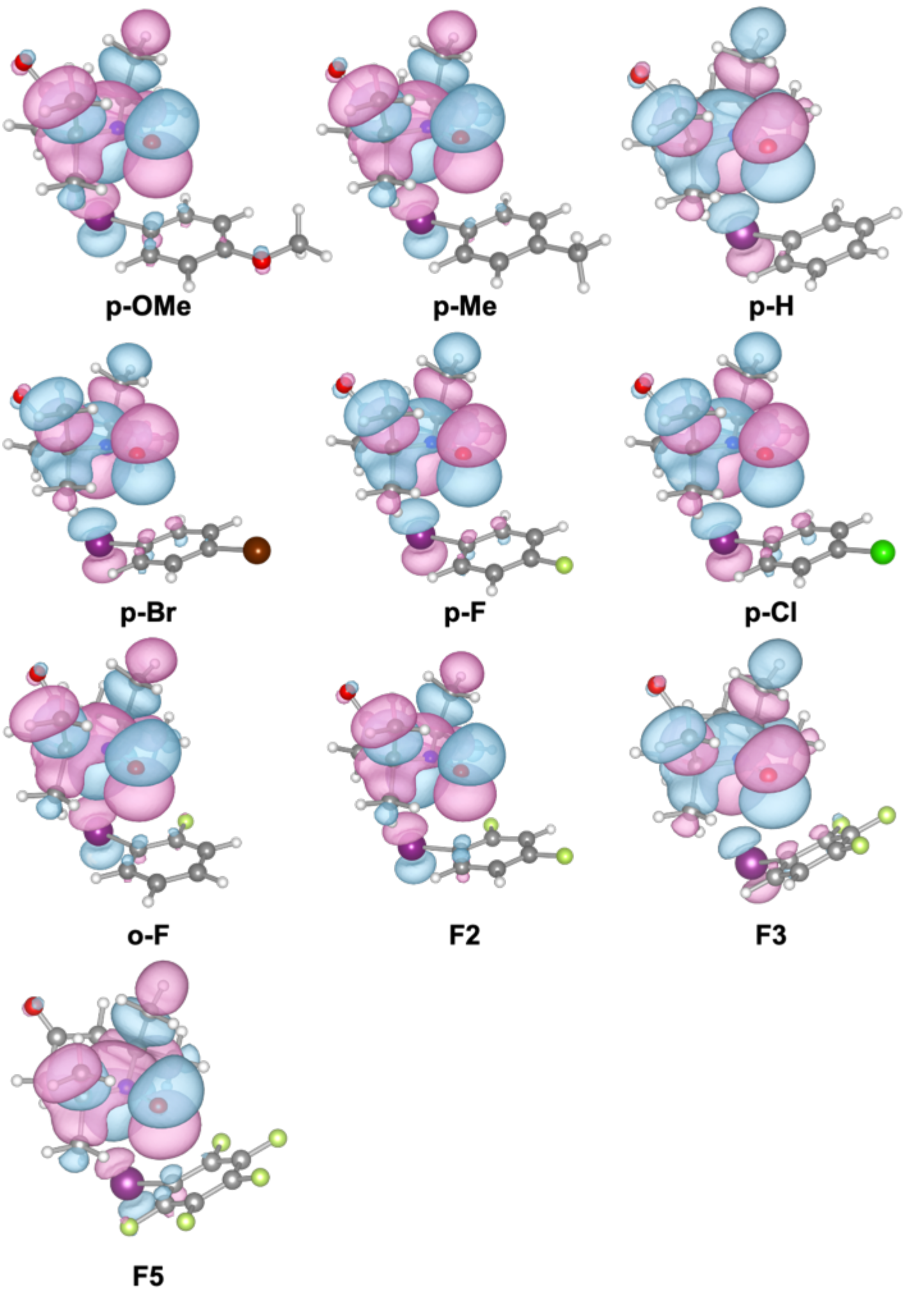

Figure S26. Singly-occupied molecular orbitals of the polarizing agent-analyte complexes between TN and the iodobenzene derivatives stabilized by the PA-π interactions. Note that the aromatic carbon-centered orbitals possess delocalized *p*-characters. Red and blue lobes represent positive and negative densities.

Electronic properties of polarizing agent-analyte complexes stabilized by various interactions

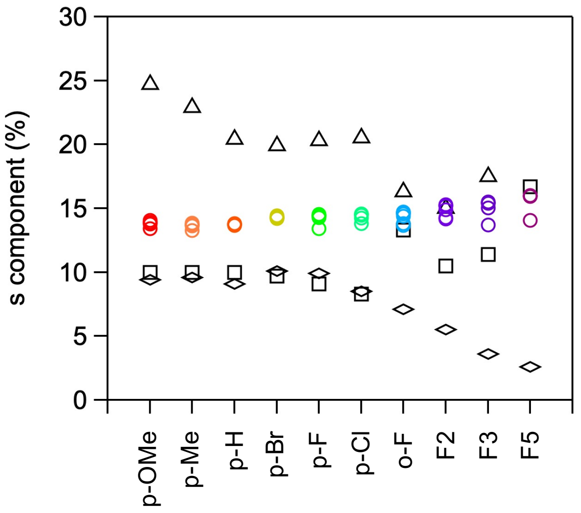

Figure S27. *s*-orbital components of the iodinated carbons of polarizing agent-analyte complexes stabilized by $O_N...I$ (colored; same scheme as main text Figure 2), $O_C...I$ (black squares), $O_N...H$ (black triangles), and PA-π (black diamonds) interactions. Analyses were performed with B3LYP-D3/aug-cc-pVTZ-J on geometries optimized with M06-2X/aug-cc-pVTZ.

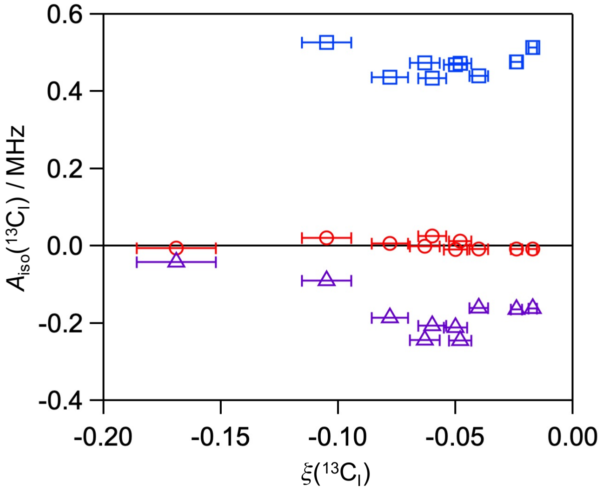

Figure S28. Relationship between $\xi(^{13}C_I)$ and $A_{iso}(^{13}C_I)$ calculated from polarizing agent-analyte complexes stabilized by the $O_C...I$ (red circles), $O_N...H$ (blue squares), and PA-π (purple triangles) interactions. No obvious correlation can be inferred. Attempted linear fits produce $r^2 = 0.04$ for $O_C...I$, $r^2 = 0.07$ for $O_N...H$, and $r^2 = 0.47$ for PA-π. Analyses were performed with B3LYP-D3/aug-cc-pVTZ-J on geometries optimized with M06-2X/aug-cc-pVTZ.

Chemical shifts of DNP and Boltzmann spectra for the iodobenzene derivatives, and relationship with hyperfine constants

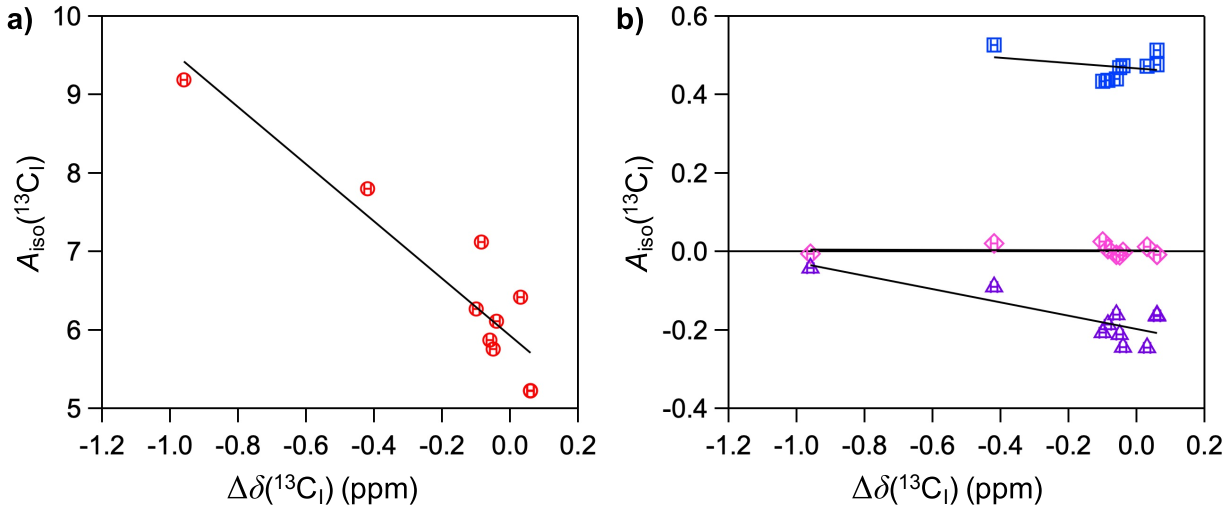

Figure S29. Correlation between $\Delta\delta(^{13}C_I)$, the chemical shift difference between DNP and Boltzmann spectra, of the iodinated carbons of the iodobenzene derivatives, and $A_{iso}(^{13}C_I)$ calculated for complexes stabilized by (a) the O_N…I halogen bond (red circles); (b) the O_C…I halogen bond (pink diamonds), the O_N…H hydrogen bond (blue squares), and the PA…π interaction (purple triangles). Black solid lines are guide-to-eye ($r^2 = 0.86$ for O_N…I, 0.0 for O_C…I, 0.10 for O_N…H, and 0.68 for PA…π). $A_{iso}(^{13}C_I)$ were evaluated with B3LYP-D3/aug-cc-pVTZ-J. Sizes of the error bars for $\Delta\delta(^{13}C_I)$ are smaller than those of the markers.

As discussed in the main text, $\Delta\delta(^{13}C_I) = \delta(^{13}C_I, \text{DNP}) - \delta(^{13}C_I, \text{Boltzmann})$ reflects the paramagnetic shift of $^{13}C_I$. Under thermal Boltzmann condition and without microwave irradiation, $\delta(^{13}C_I)$ is equal to the paramagnetic shift $\delta_{para}(^{13}C_I)$, as induced by interaction with the TN polarizing agent. Under OE-DNP, the polarizing agent EPR transition is partially saturated. Thus, $\delta(^{13}C_I)$ changes towards the diamagnetic shift $\delta_{dia}(^{13}C_I)$, i.e. the chemical shift in the absence of TN, according to the saturation factor s:[19,20]

$$\Delta\delta(^{13}C_I) = -s\left(\delta_{para}(^{13}C_I) - \delta_{dia}(^{13}C_I)\right) = -s\frac{|\gamma_e|}{\gamma_{^{13}C}}\frac{\langle A_{iso}(^{13}C_I)\rangle}{4kT}$$

where γ are the gyromagnetic ratios, T is temperature, k is the Boltzmann constant. OE-DNP measurements share the same s, due to identical instrument setup and polarizing agent concentration. Therefore, $\Delta\delta(^{13}C_I)$ should be linearly correlated with the solution-averaged isotropic hyperfine constant $\langle A_{iso}(^{13}C_I)\rangle$. Correlation between $\Delta\delta(^{13}C_I)$ and $A_{iso}(^{13}C_I)$ for the O_N…I interaction, but not the O_C…I, O_N…H, and PA-π interactions, reflect major role of O_N…I halogen bond in governing the polarizing agent-analyte spin density transfer in solution. Note that $\langle A_{iso}(^{13}C_I)\rangle$ is averaged over not only time, but also different polarizing agent-analyte interaction

mechanisms. $\langle A^2_{iso,i} \rangle$ in equation 5 of the main text, on the other hand, is averaged over time, but not over different interaction mechanisms.

Additionally, we note that all chemical shifts reported here are described by referencing to $\delta(^{13}C$, $CCl_4)$ set to 97.3 ppm. The paramagnetic shifts estimated from $\Delta\delta(^{13}C_I)$ are thus relative to that of CCl_4. This only introduces an offset to the aforementioned $\Delta\delta(^{13}C_I)$-$A_{iso}(^{13}C_I)$ relationship, and the proportionality remains valid. However, our current experimental setup does not allow measurement of the absolute paramagnetic shift due to the lack of diamagnetic references. Additionally, field drift is not compensated, as the lock field is adjusted for achieving the optimal EPR saturation (see ref.15 of the main text). Nevertheless, the relative chemical shift changes between DNP and Boltzmann measurements still provide useful insight into the polarizing agent-analyte interaction and the role of halogen bond in OE-DNP.

Bonding geometries for the brominated and chlorinated compounds

Numbering scheme of Figures S27-S32 is shared with Tables S19-S24.

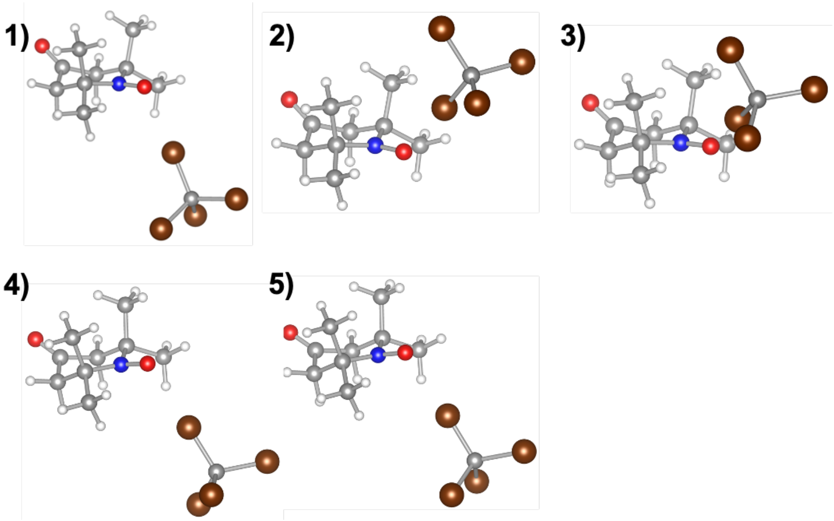

Figure S30. DFT-optimized geometries of the complexes of CBr_4 and TN stabilized by the O_N…Br halogen bond. Numbering represents individual geometries.

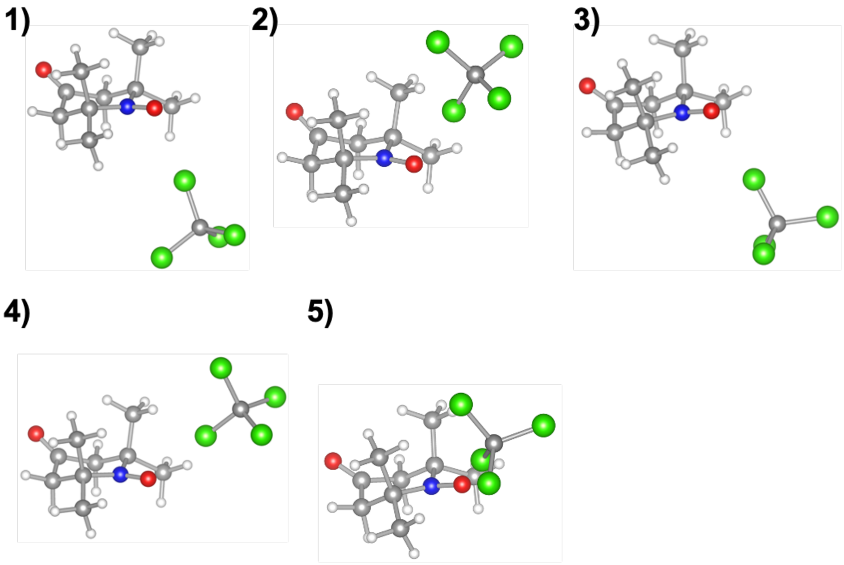

Figure S31. DFT-optimized geometries of the complexes of CCl_4 and TN stabilized by the O_N…Cl halogen bond. Numbering represents individual geometries.

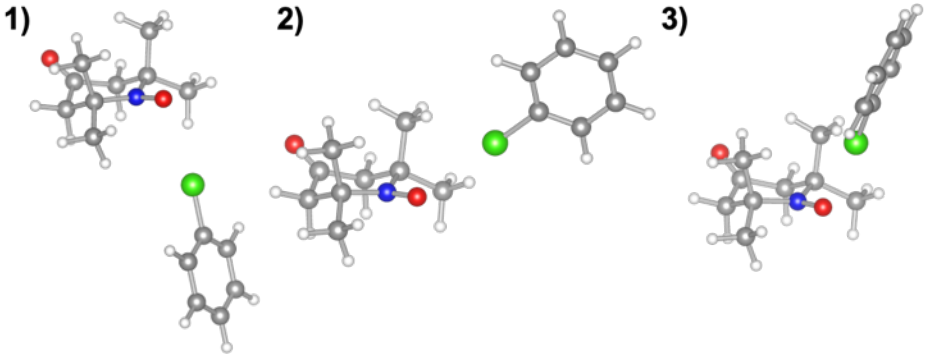

Figure S32. DFT-optimized geometries of the complexes of ClBz and TN stabilized by the O_N…Cl halogen bond. Numbering represents individual geometries.

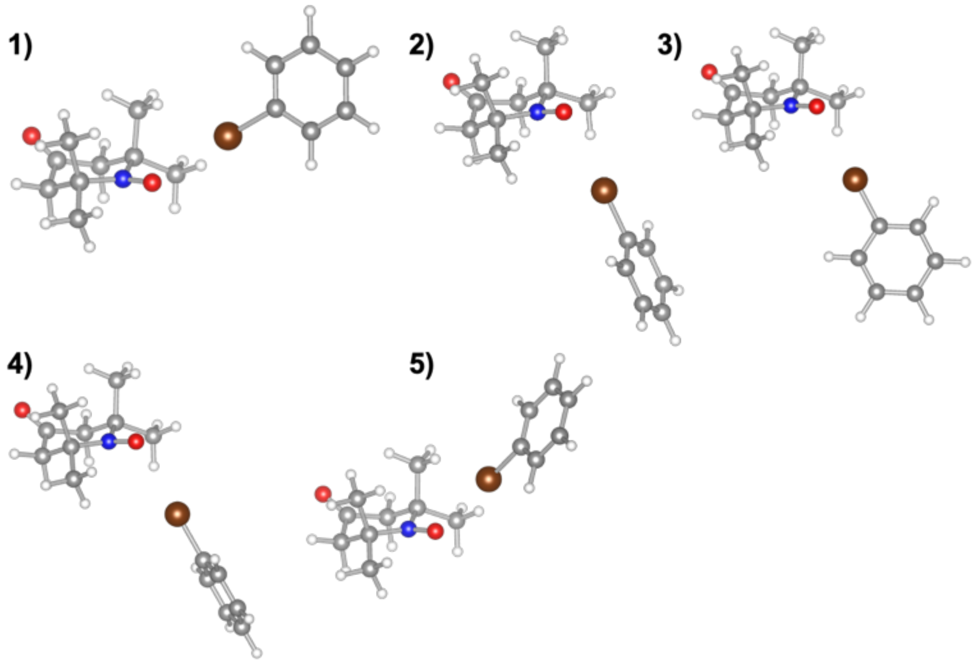

Figure S33. DFT-optimized geometries of the complexes of BrBz and TN stabilized by the O_N…Br halogen bond. Numbering represents individual geometries.

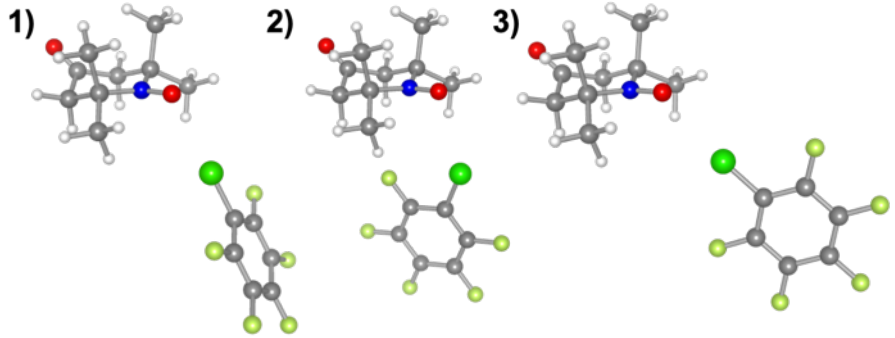

Figure S34. DFT-optimized geometries of the complexes of F_5ClBz and TN stabilized by the O_N…Cl halogen bond. Numbering represents individual geometries.

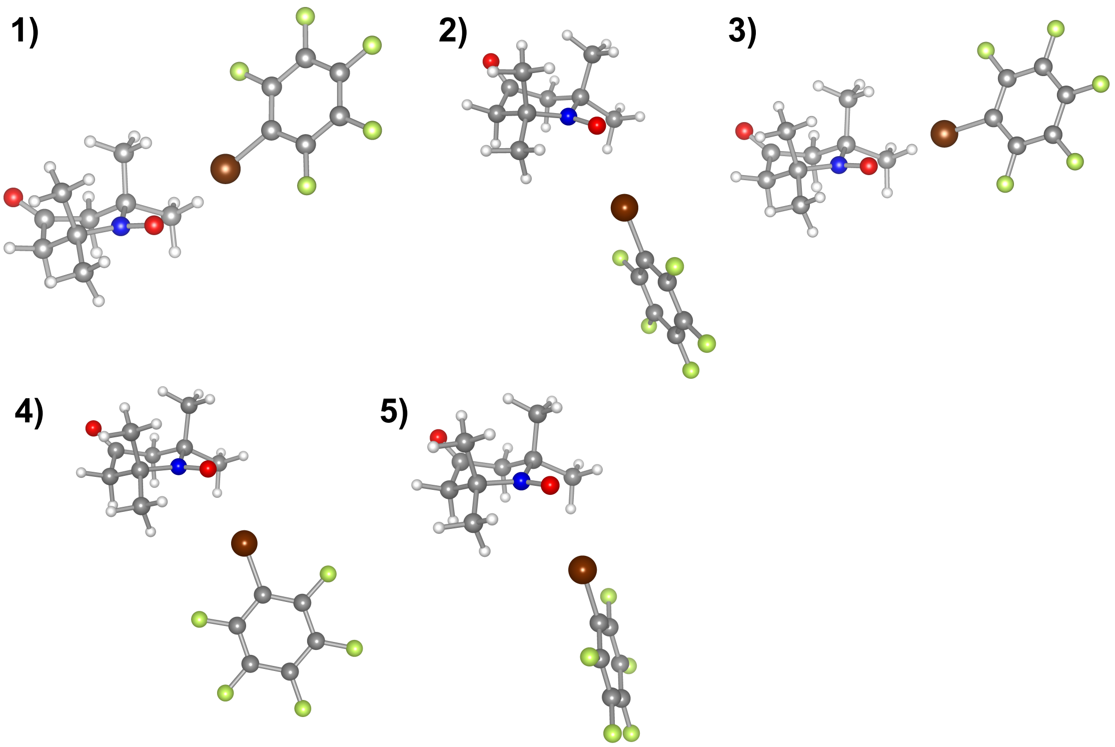

Figure S35. DFT-optimized geometries of the complexes of F_5BrBz and TN stabilized by the O_N…Br halogen bond. Numbering represents individual geometries.

Calculated properties of complexes of brominated and chlorinated analytes with TN

Table S19. Calculated parameters of geometrically optimized transient complexes of TN and CBr_4. ΔG for the most stable geometry is set to 0. See main text for definition of the parameters. For Tables S19-S25, geometry optimization was performed with M06-2X/aug-cc-pVTZ. Hyperfine interaction and spin population were evaluated by B3LYP-D3/aug-cc-pVTZ-J.

Geometries	1	2	3	4	5
ΔG / kcal·mol^{-1}	0	+0.22	+0.36	+0.50	+0.52
θ(N–O_N…Br) / °	115.19	134.55	139.72	115.14	114.55
ϕ(C–Br…O_N) / °	176.91	177.41	178.3	178.07	176.49
E(XB) / kcal·mol^{-1}	–3.40	–3.40	–3.77	–3.72	–3.33
$A_{iso}(^{13}C_{Br})$ / MHz	18.7	21.4	9.93	18.1	18.8
$\varrho(^{13}C_{Br})$ %	0.78	0.91	0.43	0.75	0.78
s component (%)	30.6	31.2	30.9	30.6	30.6

Table S20. Calculated parameters of geometrically optimized transient complexes of TN and CCl_4. ΔG for the most stable geometry is set to 0. See main text for definition of the parameters.

Geometries	1	2	3	4	5
ΔG / kcal·mol^{-1}	0	+0.65	+1.14	+1.19	+1.20
θ(N–O_N…Cl) / °	110.07	107.13	111.12	139.26	138.34
ϕ(C–Cl…O_N) / °	174.77	173.51	175.66	178.49	178.16
E(XB) / kcal·mol^{-1}	–2.72	–2.73	–2.65	–2.29	–2.31
$A_{iso}(^{13}C_{Cl})$ / MHz	8.96	8.45	8.93	6.96	3.82
$\varrho(^{13}C_{Cl})$ %	0.34	0.32	0.34	0.28	0.16
s component (%)	24.1	24.2	24.1	25.1	25.3

Table S21. Calculated parameters of geometrically optimized transient complexes of TN and ClBz. ΔG for the most stable geometry is set to 0. See main text for definition of the parameters.

Geometries	1	2	3
ΔG / kcal·mol^{-1}	0	+0.24	+0.32
θ(N–O_N…Cl) / °	115.96	146.15	139.81
ϕ(C_{Cl}–Cl…O_N) / °	168.02	169.27	162.37
E(XB) / kcal·mol^{-1}	–1.05	–0.82	–0.87
A_{iso}($^{13}C_{Cl}$) / MHz	0.69	0.97	2.40
ϱ($^{13}C_{Cl}$) %	0.05	0.08	0.20
s component (%)	10.9	11.5	11.6

Table S22. Calculated parameters of geometrically optimized transient complexes of TN and BrBz. ΔG for the most stable geometry is set to 0. See main text for definition of the parameters.

Geometries	1	2	3	4	5
ΔG / kcal·mol^{-1}	0	+0.04	+0.24	+0.48	+0.71
θ(N–O_N…Br) / °	140.32	107.02	115.82	112.62	139.00
ϕ(C_{Br}–Br…O_N) / °	178.22	174.21	172.75	178.65	177.45
E(XB) / kcal·mol^{-1}	–1.40	–1.63	–1.63	–1.62	–1.44
A_{iso}($^{13}C_{Br}$) / MHz	1.46	2.34	2.86	2.88	2.69
ϱ($^{13}C_{Br}$) %	0.15	0.22	0.27	0.27	0.26
s component (%)	11.9	12.6	12.6	12.5	12.7

Table S23. Calculated parameters of geometrically optimized transient complexes of TN and F_5ClBz. ΔG for the most stable geometry is set to 0. See main text for definition of the parameters.

Geometries	1	2	3
ΔG / kcal·mol^{-1}	0	+0.27	+0.89
θ(N–O_N…Cl) / °	127.00	113.92	97.80
ϕ(C_{Cl}–Cl…O_N) / °	173.20	175.23	143.13
E(XB) / kcal·mol^{-1}	–2.26	–2.26	–2.57
A_{iso}($^{13}C_{Cl}$) / MHz	3.11	3.49	1.41
ϱ($^{13}C_{Cl}$) %	0.19	0.21	0.08
s component (%)	14.1	13.8	14.1

Table S24. Calculated parameters of geometrically optimized transient complexes of TN and F_5BrBz. ΔG for the most stable geometry is set to 0. See main text for definition of the parameters.

Geometries	1	2	3	4	5	6
ΔG / kcal·mol^{-1}	0	+0.01	+0.04	+0.06	+0.25	+0.78
θ(N–O_N…Br) / °	140.41	116.20	150.35	115.83	108.74	98.70
ϕ(C_{Br}–Br…O_N) / °	175.56	176.69	172.29	172.75	169.80	133.82
E(XB) / kcal·mol^{-1}	–3.14	–3.40	–3.02	–3.42	–3.31	–2.62
$A_{iso}(^{13}C_{Br})$ / MHz	4.42	6.22	2.26	6.24	5.59	1.36
$\varrho(^{13}C_{Br})$ %	0.35	0.45	0.19	0.46	0.40	0.10
s component (%)	14.6	15.3	13.6	15.4	15.5	16.1

Table S25. Averaged values of calculated parameters of geometrically optimized transient complexes of TN and the brominated and chlorinated compounds. X represent the heaviest halogen in each compound.

Analyte	E(XB) / kcal·mol^{-1}	$A_{iso}(^{13}C_X)$ / MHz	$\varrho(^{13}C_X)$ / %	s-orbital component / %
CBr_4	–3.52	17.68	0.74	30.8
CCl_4	–2.54	8.31	0.31	24.3
ClBz	–0.92	1.22	0.10	11.3
BrBz	–1.54	2.29	0.21	12.4
F_5ClBz	–2.24	3.03	0.19	13.9
F_5BrBz	–3.28	4.70	0.35	14.9

Relationship between DNP performance and hyperfine constants for all halogenated compounds investigated in this study

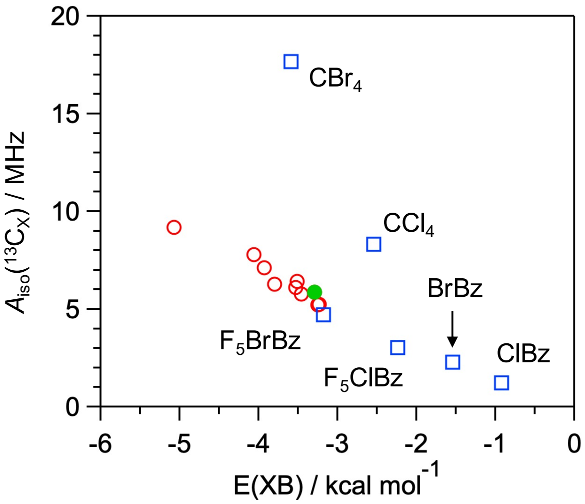

Figure S36. Correlation between $A_{iso}(^{13}C_X)$ and E(XB) for polarizing agent-analyte complexes stabilized by the $O_N \ldots X$ halogen bond. Red empty circles represent iodinated compounds. Blue squares represent brominated and chlorinated compounds. Green filled circle marks iodobenzene. E(XB) and $A_{iso}(^{13}C_X)$ were obtained from calculations with M06-2X and B3LYP-D3, respectively.

Correlation between halogen bond properties and DNP performance measured in cyclopentane

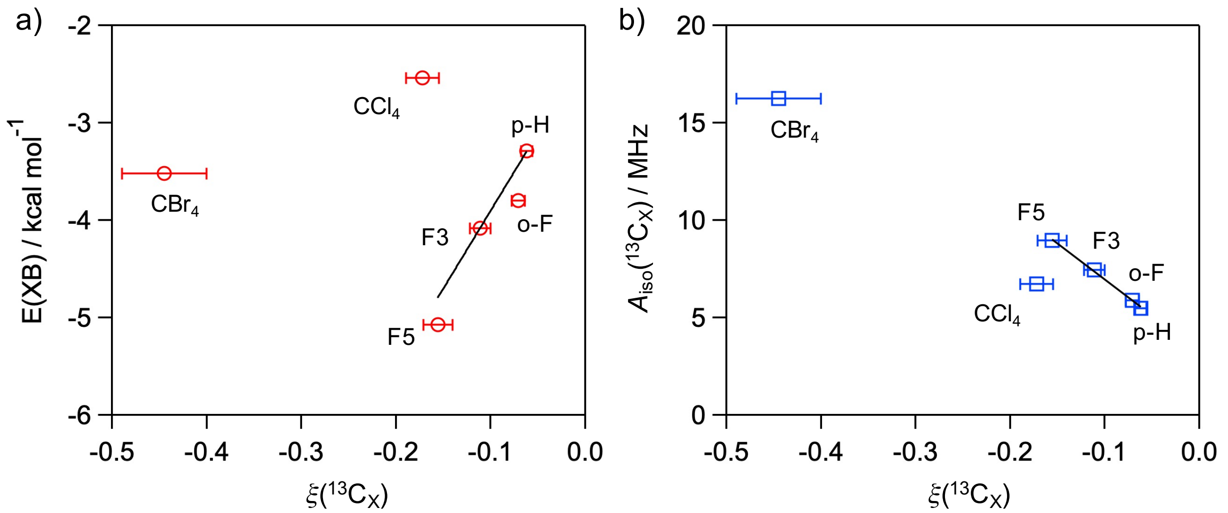

Figure S37. Correlation between (a) E(XB)-$\xi(^{13}C_X)$ and (b) $A_{iso}(^{13}C_X)$-$\xi(^{13}C_X)$ based on experimental data obtained from measurements of analytes dissolved in cyclopentane. Black lines are guides-to-eye, with $r^2 = 0.94$ (a) and $r^2 > 0.99$ (b). Note that the leakage and saturation factors were taken from those for CCl_4. E(XB) were calculated from geometries optimized with M06-2X/aug-cc-pVTZ. $A_{iso}(^{13}C_X)$ were calculated from optimized geometries using PBE0/x2c-TZVPall-s.

Electrostatic potential isosurface maxima of halogenated molecules

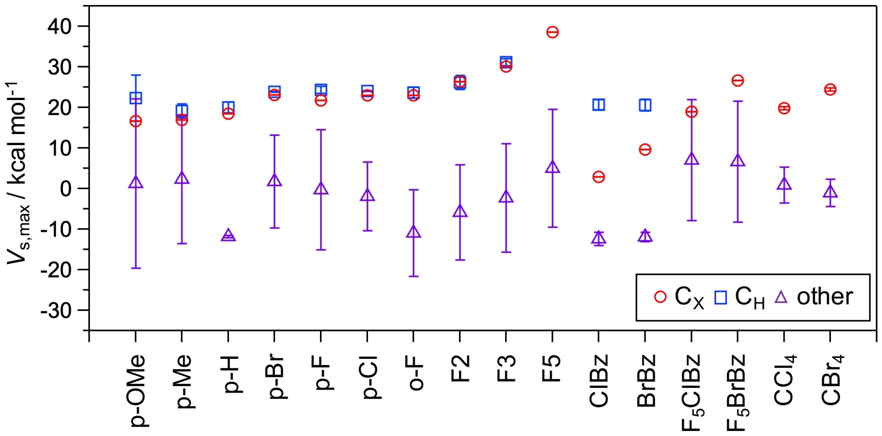

Figure S38. Electrostatic potential isosurface maxima ($V_{s,max}$) for investigated halogenated compounds. Red circles, blue squares, and purple triangles correspond to values for XB, proton, and the rest part of the molecules, respectively. Vertical bars represent range of $V_{s,max}$.

Alternative binding modes of CX_4 with TN

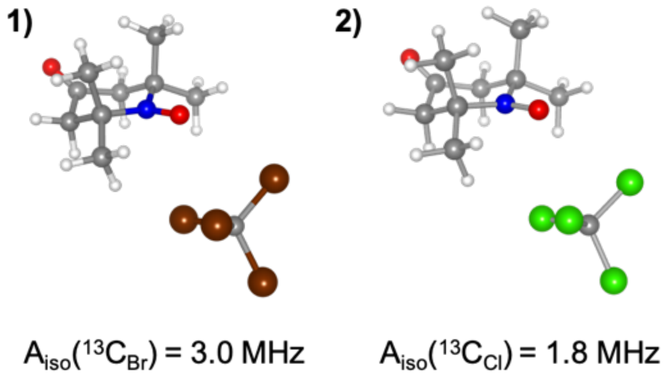

Figure S39. Alternative CX_4-TN halogen bonded geometries giving rise to non-negligible $A_{iso}(^{13}C_X)$ beside through the O_N…X halogen bonds. Specifically, CX_4 interacts with the TN radical site through facial binding (i.e., the $-CX_3$ cone).

Relative surface area of the σ-hole of the halogenated analytes

Table S26. Relative surface area of the XB σ-hole of the halogenated analytes. Here, the surface areas of the σ-hole refer to the region with $0 \leq V_s \leq V_{s,max}$(XB) near the halogen involved in XB, evaluated using Multiwfn[9] at isosurface described in Figure S3. Relative surface area is calculated by the surface area of the σ-hole divided by the total surface area of the analyte electrostatic potential isosurface.

Analyte	Relative surface area (%)
p-OMe	3.04
p-Me	4.40
p-H	5.66
p-Br	6.65
p-F	7.12
p-Cl	7.29
o-F	6.87
F2	8.52
F3	13.88
F5	20.88
ClBz	0.87
BrBz	2.90
F_5ClBz	12.53
F_5BrBz	15.91
CCl_4	41.62
CBr_4	38.96

Chemical shift differences between the DNP and Boltzmann spectra for the iodinated, brominated, and the chlorinated analytes

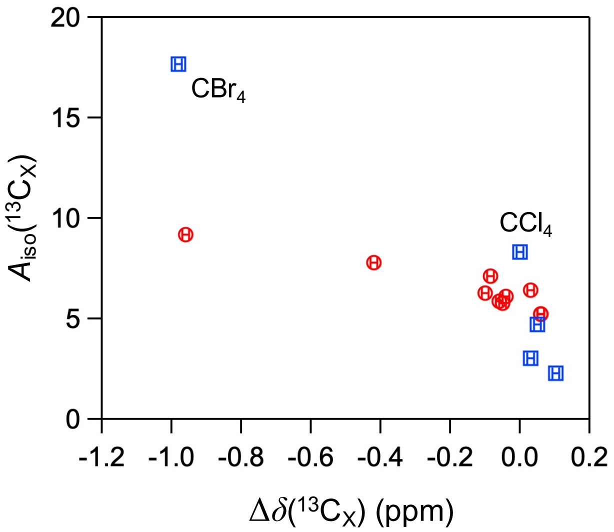

Figure S40. Correlation between $\Delta\delta(^{13}C_X)$ and $A_{iso}(^{13}C_X)$ calculated for complexes stabilized by the O_N…X halogen bond. Red circles represent iodinated analytes. Blue squares represent brominated and chlorinated analytes. $A_{iso}(^{13}C_I)$ were evaluated with B3LYP-D3/aug-cc-pVTZ-J. Sizes of the error bars for $\Delta\delta(^{13}C_X)$ are smaller than those of the markers.

Vibrational modes of halogen bond stabilized analyte-polarizing agent complexes

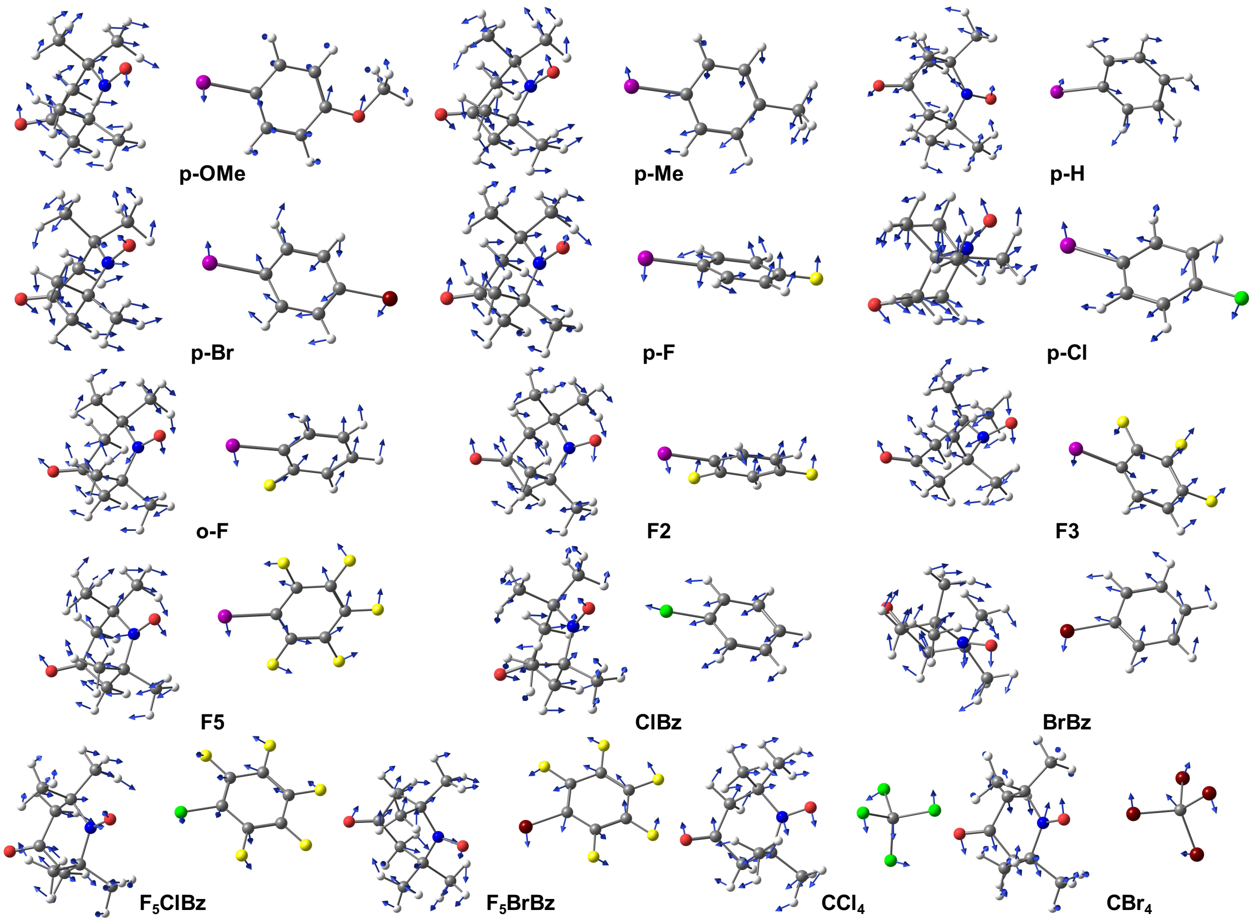

Figure S41. Lowest energy vibrational modes for analyte-polarizing agent complexes stabilized by the $O_N \ldots X$ (X = heaviest halogen) interactions, obtained from geometries with lowest Gibbs free energies for respective analytes. Arrows correspond to displacement vectors.

Vibrational frequencies and OE-DNP spectral density function

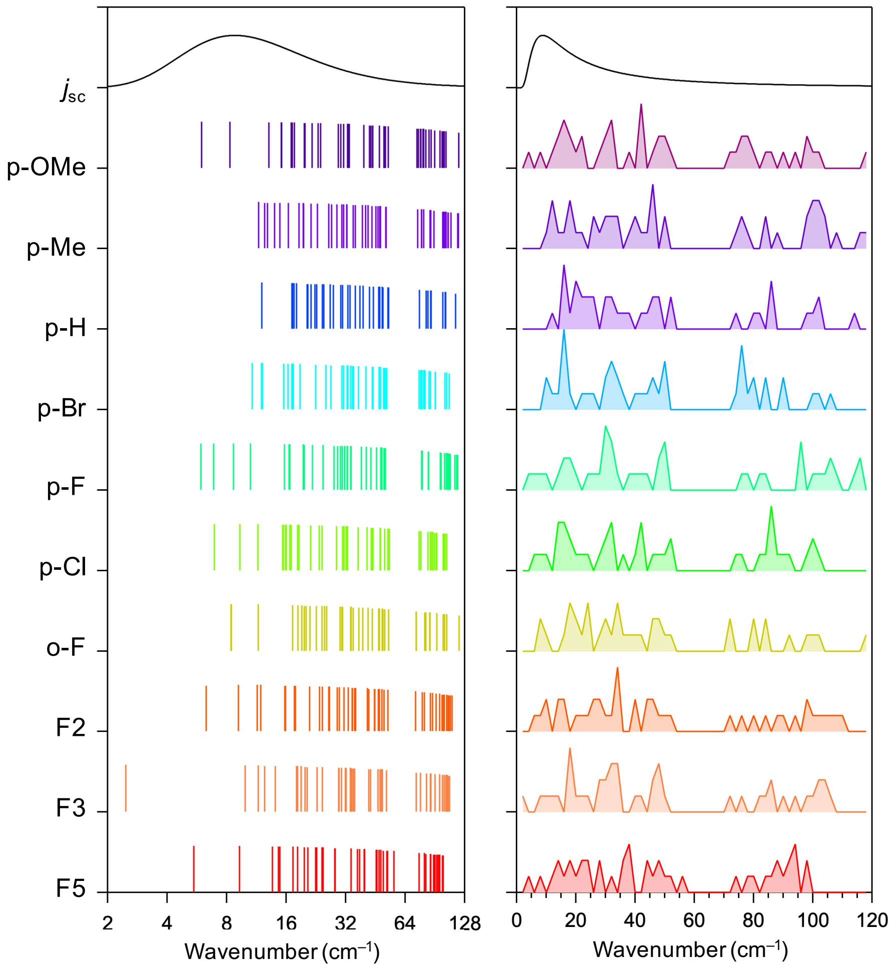

Figure S42. Summary of vibrational frequencies computed for optimized polarizing agent-analyte complexes of the iodobenzene derivatives stabilized by the N–O_N…I halogen bond. Left: frequencies of vibrational levels and relative populations obtained from numerical frequency calculations; Right: histogram of vibrational frequencies (bin size: 2 cm^{-1}, starting from 2 cm^{-1}). Black traces are spectral density function (j_{sc}) simulated using parameters for benzene taken from Supplementary Figure 23 of Ref. 15 in the main text. Efficient OE-DNP is expected when frequencies of vibrational modes overlap with the region where j_{sc} is close to maximum. Note that the x-axis in the left panel is plotted on logarithmic scale, whereas that in the right panel is on linear scale.

Benchmarking vibrational frequency calculation against experimental measurements

Table S27. Calculated and experimental infrared spectral features of selected halogen-bonded complexes. Experimental data and assignments were taken from previous studies.[21–23] Vibrational frequencies were computed following the same approach as for the polarizing agent-analyte complexes presented here, with geometries obtained from the crystal structures without further optimization. Reasonable agreement is seen between experimental and vibrational frequencies, which supports potential contribution to OE-DNP from vibration of the polarizing agent-analyte complexes.

Compound	Vibrational Mode	Experiment (cm^{-1})	Calculation (cm^{-1})
diiodo-tetrafluorobenzene with piperazine*	C–I stretch	229	238.4
	N–I stretch	67	64.3
pentafluoroiodobenzene with pyridine*	C–I bend	140	142.0
	C–I stretch	193	195.1
pentafluoroiodobenzene with 4-methyl-pyridine*	C–I bend	146	144.3
	C–I stretch	192	196.8
iodocyanide with pyridine#	C–I bend	336	363.1
	C–I stretch	430	448.7

**in solid state; #in liquid state*

Polarizing agent-analyte complex vibration induced isotropic hyperfine constant variations

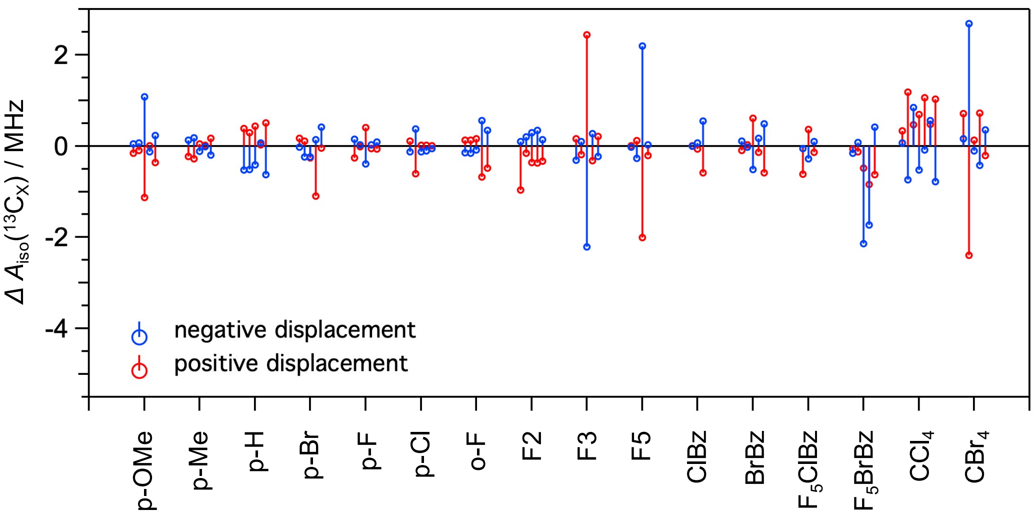

Figure S43. Variations in $A_{iso}(^{13}C_X)$ during lowest energy vibrational motions for the geometry with lowest Gibbs free energy for each polarizing agent-analyte complex. Atoms were displaced by plus (red) or minus (blue) unit length along individual displacement vectors marked in Figure S41 (without further geometry optimization). Analyses were performed with M06-2X/aug-cc-pVTZ.

References

(1) Neese, F.; Wennmohs, F.; Becker, U.; Riplinger, C. The ORCA Quantum Chemistry Program Package. *J. Chem. Phys.* **2020**, *152* (22). https://doi.org/10.1063/5.0004608.

(2) Zhao, Y.; Truhlar, D. G. The M06 Suite of Density Functionals for Main Group Thermochemistry, Thermochemical Kinetics, Noncovalent Interactions, Excited States, and Transition Elements: Two New Functionals and Systematic Testing of Four M06-Class Functionals and 12 Other Function. *Theor. Chem. Acc.* **2008**, *120* (1–3), 215–241. https://doi.org/10.1007/s00214-007-0310-x.

(3) Grimme, S.; Antony, J.; Ehrlich, S.; Krieg, H. A Consistent and Accurate Ab Initio Parametrization of Density Functional Dispersion Correction (DFT-D) for the 94 Elements H-Pu. *J. Chem. Phys.* **2010**, *132* (15), 154104. https://doi.org/10.1063/1.3382344.

(4) Kendall, R. A.; Dunning, T. H.; Harrison, R. J. Electron Affinities of the First-Row Atoms Revisited. Systematic Basis Sets and Wave Functions. *J. Chem. Phys.* **1992**, *96* (9), 6796–6806. https://doi.org/10.1063/1.462569.

(5) Woon, D. E.; Dunning, T. H. Gaussian Basis Sets for Use in Correlated Molecular Calculations. III. The Atoms Aluminum through Argon. *J. Chem. Phys.* **1993**, *98* (2), 1358–1371. https://doi.org/10.1063/1.464303.

(6) Peterson, K. A.; Shepler, B. C.; Figgen, D.; Stoll, H. On the Spectroscopic and Thermochemical Properties of ClO, BrO, IO, and Their Anions. *J. Phys. Chem. A* **2006**,

110 (51), 13877–13883. https://doi.org/10.1021/jp065887l.
(7) Neese, F.; Wennmohs, F.; Hansen, A.; Becker, U. Efficient, Approximate and Parallel Hartree–Fock and Hybrid DFT Calculations. A 'Chain-of-Spheres' Algorithm for the Hartree–Fock Exchange. *Chem. Phys.* **2009**, *356* (1–3), 98–109. https://doi.org/10.1016/j.chemphys.2008.10.036.
(8) Marenich, A. V.; Cramer, C. J.; Truhlar, D. G. Universal Solvation Model Based on Solute Electron Density and on a Continuum Model of the Solvent Defined by the Bulk Dielectric Constant and Atomic Surface Tensions. *J. Phys. Chem. B* **2009**, *113* (18), 6378–6396. https://doi.org/10.1021/jp810292n.
(9) Lu, T.; Chen, F. Multiwfn: A Multifunctional Wavefunction Analyzer. *J. Comput. Chem.* **2012**, *33* (5), 580–592. https://doi.org/10.1002/jcc.22885.
(10) Lu, T.; Chen, F. Quantitative Analysis of Molecular Surface Based on Improved Marching Tetrahedra Algorithm. *J. Mol. Graph. Model.* **2012**, *38*, 314–323. https://doi.org/10.1016/j.jmgm.2012.07.004.
(11) Davis, D. G.; Bax, A. Simplification of Proton NMR Spectra by Selective Excitation of Experimental Subspectra. *J. Am. Chem. Soc.* **1985**, *107* (24), 7197–7198. https://doi.org/10.1021/ja00310a085.
(12) Franzke, Y. J.; Treß, R.; Pazdera, T. M.; Weigend, F. Error-Consistent Segmented Contracted All-Electron Relativistic Basis Sets of Double- and Triple-Zeta Quality for NMR Shielding Constants. *Phys. Chem. Chem. Phys.* **2019**, *21* (30), 16658–16664. https://doi.org/10.1039/c9cp02382h.
(13) Rega, N.; Cossi, M.; Barone, V. Development and Validation of Reliable Quantum Mechanical Approaches for the Study of Free Radicals in Solution. *J. Chem. Phys.* **1996**, *105* (24), 11060–11067. https://doi.org/10.1063/1.472906.
(14) Huzinaga, S. Gaussian-Type Functions for Polyatomic Systems. I. *J. Chem. Phys.* **1965**, *42* (4), 1293–1302. https://doi.org/10.1063/1.1696113.
(15) Rolfes, J. D.; Neese, F.; Pantazis, D. A. All-Electron Scalar Relativistic Basis Sets for the Elements Rb–Xe. *J. Comput. Chem.* **2020**, *41* (20), 1842–1849. https://doi.org/10.1002/jcc.26355.
(16) Provasi, P. F.; Aucar, G. A.; Sauer, S. P. A. The Effect of Lone Pairs and Electronegativity on the Indirect Nuclear Spin-Spin Coupling Constants in CH2X (X=CH2, NH, O, S): Ab Initio Calculations Using Optimized Contracted Basis Sets. *J. Chem. Phys.* **2001**, *115* (3), 1324–1334. https://doi.org/10.1063/1.1379331.
(17) Saitow, M.; Neese, F. Accurate Spin-Densities Based on the Domain-Based Local Pair-Natural Orbital Coupled-Cluster Theory. *J. Chem. Phys.* **2018**, *149* (3), 034104. https://doi.org/10.1063/1.5027114.
(18) Gromov, O. I. Performance of the DLPNO-CCSD and Recent DFT Methods in the Calculation of Isotropic and Dipolar Contributions to 14N Hyperfine Coupling Constants of Nitroxide Radicals. *J. Mol. Model.* **2021**, *27* (6), 194. https://doi.org/10.1007/s00894-021-04807-z.
(19) Wind, R. A.; Lock, H.; Mehring, M. 13C Knight Shift Saturation and 1H Dynamic Nuclear Polarization in a Polycrystalline Sample of the Organic Conductor (Fluoranthenyl)2PF6. *Chem. Phys. Lett.* **1987**, *141* (4), 283–288. https://doi.org/10.1016/0009-2614(87)85024-8.
(20) Russ, J. L.; Gu, J.; Tsai, K.-H.; Glass, T.; Duchamp, J. C.; Dorn, H. C. Nitroxide/Substrate Weak Hydrogen Bonding: Attitude and Dynamics of Collisions in Solution. *J. Am. Chem.*

Soc. **2007**, *129* (22), 7018–7027. https://doi.org/10.1021/ja064632i.
(21) Kalout, H.; Boubegtiten-Fezoua, Z.; Maurel, F.; Hellwig, P.; Ferlay, S. An Accurate Vibrational Signature in Halogen Bonded Molecular Crystals. *Phys. Chem. Chem. Phys.* **2022**, *24* (24), 15103–15109. https://doi.org/10.1039/d2cp01336c.
(22) Vasylyeva, V.; Catalano, L.; Nervi, C.; Gobetto, R.; Metrangolo, P.; Resnati, G. Characteristic Redshift and Intensity Enhancement as Far-IR Fingerprints of the Halogen Bond Involving Aromatic Donors. *CrystEngComm* **2016**, *18* (13), 2247–2250. https://doi.org/10.1039/c5ce02385h.
(23) Person, W. B.; Humphrey, R. E.; Popov, A. I. Infrared Spectra of Charge Transfer Complexes. II. Iodine Cyanide Complexes. *J. Am. Chem. Soc.* **1959**, *81* (2), 273–277. https://doi.org/10.1021/ja01511a004.
